# Cell Fate Reprogramming by Control of Intracellular Network Dynamics

**DOI:** 10.1371/journal.pcbi.1004193

**Published:** 2015-04-07

**Authors:** Jorge G. T. Zañudo, Réka Albert

**Affiliations:** 1 Department of Physics, The Pennsylvania State University, University Park, Pennsylvania, United States of America; 2 Department of Biology, The Pennsylvania State University, University Park, Pennsylvania, United States of America; Ecole Normale Supérieure, FRANCE

## Abstract

Identifying control strategies for biological networks is paramount for practical applications that involve reprogramming a cell’s fate, such as disease therapeutics and stem cell reprogramming. Here we develop a novel network control framework that integrates the structural and functional information available for intracellular networks to predict control targets. Formulated in a logical dynamic scheme, our approach drives any initial state to the target state with 100% effectiveness and needs to be applied only transiently for the network to reach and stay in the desired state. We illustrate our method’s potential to find intervention targets for cancer treatment and cell differentiation by applying it to a leukemia signaling network and to the network controlling the differentiation of helper T cells. We find that the predicted control targets are effective in a broad dynamic framework. Moreover, several of the predicted interventions are supported by experiments.

## Author Summary

Practical applications in modern molecular and systems biology such as the search for new therapeutic targets for diseases and stem cell reprogramming have generated a great interest in controlling the internal dynamics of a cell. Here we present a network control approach that integrates the structural and functional information of the network. We show that stabilizing the expression or activity of a few select components can drive the cell towards a desired fate or away from an undesired fate. We demonstrate our method’s effectiveness by applying it to a type of blood cell cancer and to the differentiation of a type of immune cell. Overall, our approach provides new insights into how to control the dynamics of intracellular networks.

This is a *PLOS Computational Biology* Methods paper.

## Introduction

An important task of modern molecular and systems biology is to achieve an understanding of the dynamics of the network of macromolecular interactions that underlies the functioning of cells. Practical applications such as stem cell reprogramming [1–3] and the search for new therapeutic targets for diseases [4–6] have also motivated a great interest in the general task of cell fate reprogramming, i.e., controlling the internal state of a cell so that it is driven from an initial state to a final target state (see references [7–13]).

Theoretically derived control methods are based on simplified models of the interactions and/or the dynamics of cellular constituents such as proteins or mRNAs. Some of these models only include information on which cell components (e.g. molecules or proteins) interact among each other, i.e., the structure of the underlying interaction network. Other models, known as dynamic models, include the structure of the interaction network and also an equation for each component, which describes how the state of this component changes in time due to the influence of other cell components (e.g. how the concentration of a molecule changes in time due to the reactions the molecule participates in).

Although the topic of network controllability has a long history in control and systems theory (see, for example, [14–17]), most of this work is not directly applicable to large intracellular networks. There are several reasons for this: (i) combinatorial complexity and the size of the matrices involved makes control theory applicable to small networks only, (ii) linear functions are used for the regulatory functions and it is unclear how the switch-like behavior of many biochemical processes [18, 19] will affect these results, and (iii) the notion of controllability in control theory, i.e. control of the full set of states [14–16] or *complete controllability*, is different from that in the biological sense, which commonly encompasses only the *biologically admissible states*[8].

In recent work on network controllability [7, 9–13, 20–22] some of the limitations of standard control theory approaches are addressed. For example, Akutusu, Cheng, Tamura et al. [20–22] extend the framework of control theory to systems with Boolean (switch-like) dynamics and provide some formal results in this setting. In the work of Liu et al. [7] the size limitation of linear control theory is overcome by using a maximal matching approach to identify the minimal number of nodes needed to control a variety of real-world large scale networks. Specifically, for some gene regulatory networks, Liu et al. find that control of roughly 80% of the nodes is needed to fully control the dynamics of these networks [7]. In contrast, experimental work in stem cell reprogramming suggests that for biologically admissible states the number of nodes required for control is drastically lower (five or fewer genes [1–3, 8]). Fiedler, Mochizuki et al. [12, 13] use the concept of the feedback vertex set, a subset of nodes in a directed network whose removal leaves the graph without directed cycles (i.e. without feedback loops). They show that, for a broad class of regulatory functions, controlling any feedback vertex set is enough to guide the dynamics of the system to any target trajectory of the uncontrolled network [12, 13]. As one of their examples, the authors use a signal transduction network with 113 elements and show that the minimal feedback vertex set is composed of only 5 elements.

Since systems whose interaction networks and dynamics are known equally well are rare, current control strategies are based on either the network structure [7, 9, 10, 12, 13] or its dynamics (function) [11, 20–22]. Yet, as manipulating the activity of even a single intracellular component is a long, difficult, and expensive experimental task, it is crucial to reduce as much as possible the number of nodes that need to be controlled. We hypothesize that integrating network structure with qualitative information on the regulatory functions or on the target states of interest could yield control strategies with a small number of control targets. Qualitative information about the regulatory functions is commonly known (e.g. positive/negative regulation, cooperativity among regulators, etc.), and relative qualitative information on the desired/undesired states also exists (e.g. upregulation or downregulation of mRNA levels in a disease state with respect to a healthy state). Thus, we choose a logical dynamic framework as our modeling method [23]. This framework is well suited for modeling intracellular networks: discrete dynamic models have been shown to reproduce the qualitative dynamics of a multitude of cellular systems while requiring only the combinatorial activating or inhibiting nature of the interactions, and not the kinetic details [24–30].

Logical dynamic network models [31–38] consist of a set of binary variables {*σ*_*i*_}, *i* = 1,2,…,*N*, each of which denotes the state of a node (also referred to as node state). The state ON (or 1) commonly refers to above a certain threshold level, while the state OFF (or 0) refers to below the same threshold level. The vector formed by the state of all nodes (*σ*_1_,*σ*_2_,…,*σ*_*N*_) denotes the state of the system (or system/network state). To each node *v*_*i*_ one assigns a Boolean function *f*_*i*_ which contains the biological information on how node *v*_*i*_’s inputs influence *σ*_*i*_; these functions are used to evolve in time the state of each element. We use the general asynchronous updating scheme [33, 34, 36] (see Methods), a stochastic scheme which takes into consideration the variety of timescales present in intracellular processes and our incomplete knowledge of the rates of these processes.

In a logical (Boolean) model, every temporal trajectory must eventually reach a set of system states in which it settles down, known as an attractor. The attractors of intracellular networks have been found to be identifiable with different cell fates, cell behaviors, and stable patterns of cell activity [24–30, 39, 40]. In general, the task of finding Boolean network attractors is limited by combinatorial complexity; the size of the state space grows exponentially with the number of nodes *N*. To address this, we recently proposed an alternative approach to find the attractors of a Boolean network which allowed us to identify the attractors of networks for which a full search of the state space is not feasible [41]. This attractor-finding method is based on identifying certain function-dependent network components, referred to as *stable motifs*, that must stabilize in a fixed state. A stable motif is defined as a set of nodes and their corresponding states which are such that the nodes form a minimal strongly connected component (e.g. a feedback loop) and their states form a partial fixed point of the Boolean model. (A partial fixed point is a subset of nodes and a respective state for each of these nodes such that updating any node in the subset leaves its state unchanged, regardless of the state of the nodes outside the subset.) It is noteworthy that stable motifs are preserved for other updating schemes because of their dynamical property of being partial fixed points. For more details on the attractor-finding method and the identification of the stable motifs see S1 Text and ref. [41]; for a more formal and mathematical discussion see S2 Text section A or Appendix A of ref. [41].

Once a network’s stable motifs and their corresponding fixed states are identified, a network reduction technique [36, 42–44] is used for each stable motif by tracing the downstream effect of the stable motif on the rest of the network (see S1 Text). Repeating this procedure iteratively for each separate stable motif until no new stable motifs are found yields the attractors of the logical model. Formally, the result is a set of network states called quasi-attractors, which capture steady states exactly and are a compressed representation of complex attractors [41]. The network control method we propose here builds on the concept of stable motifs and its relation to (quasi-)attractors [41] and takes it much further by connecting stable motifs with a way to identify targets whose manipulation (upregulation or downregulation) ensures the convergence of the system to an attractor of interest. The use of quasi-attractors in our method does not compromise its general applicability, but it does require that certain networks with special types of complex attractors are treated with care when our method is applied. None of the networks we discuss in this work nor any intracellular network models we are aware of fall in this category; for more details see S1 Text, S2 Text, and ref [41].)

**Fig 1:**
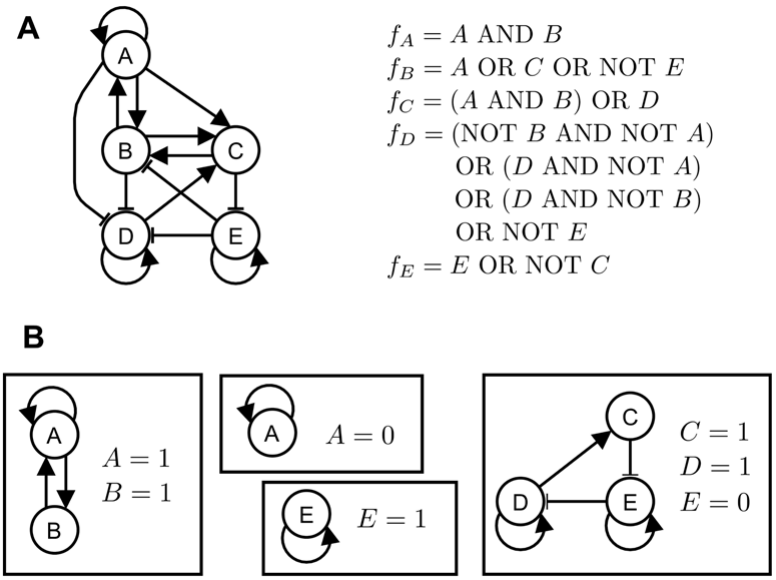
Stable motifs of a logical (Boolean) network. (a) An example of a logical network indicating the regulatory relationships and the logical update function of each node. (b) The four stable motifs of the logical network in (a) and their corresponding node states. These stable motifs are strongly connected components and partial fixed points of the logical network.

**Fig 2:**
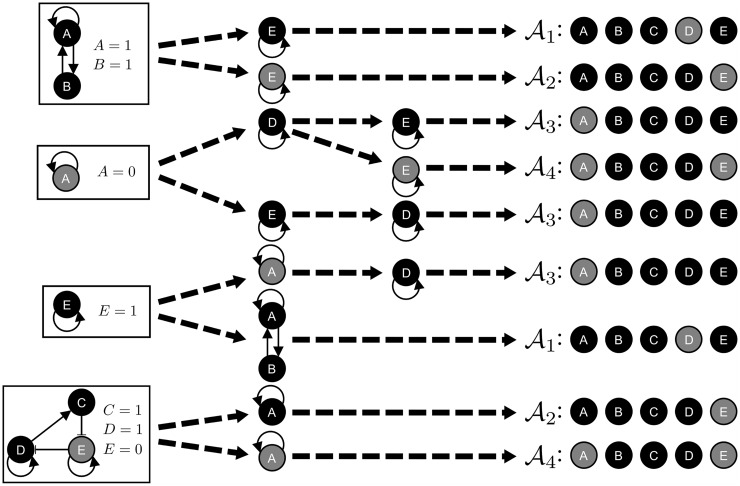
Stable motif succession diagram for the example in Fig 1. The stable motif succession diagram shows the stable motifs obtained successively during the attractor finding process and the attractors they finally lead to. A more detailed representation of the first steps of the attractor finding method is shown in S1 Fig. Nodes are colored based on their respective node states in the motifs or the attractors: gray for 0 and black for 1. The four stable motifs of the original logical network and their matching node states are shown in the leftmost part of the figure. The attractors obtained for each possible sequence of stable motifs are shown in the rightmost part of the figure. The result of applying network reduction using a stable motif is represented by each dashed arrow. If network reduction due to a stable motif leads to a simplified network with at least one stable motif, then the dashed arrows point from the stable motif being considered to the stable motifs of the simplified network. Otherwise, network reduction leads directly to an attractor and the dashed arrow points towards the attractor.

As an illustration, consider the logical network shown in Fig 1(a). This logical network has four stable motifs (Fig 1(b)): (i) {A = 1, B = 1}, (ii) {A = 0}, (iii) {E = 1}, and (iv) {C = 1, D = 1, E = 0}. Network reduction for each of these stable motif yields four reduced networks, each of which has its own stable motifs, all of which are shown in S1 Fig. For example, the reduced logical network obtained from the first stable motif consists of two nodes (D and E) and has two stable motifs: {E = 1} and {E = 0}. The stable motifs of the remaining three reduced logical networks are, respectively: {E = 1} and {D = 1}; {A = 1, B = 1} and {A = 0}; {A = 1} and {A = 0}. Repeating the same network reduction procedure with each of the new stable motifs leads to either a new reduced network or one of four attractors (𝒜_*i*_,*i* = 1,…,4). The stable motifs obtained from the original network and from each reduced network, and the attractors they lead to are shown in Fig 2. This diagram is a compressed representation of the successive steps of the attractor finding process, which include the original network, the stable motifs of the original network, the reduced networks obtained for each stable motif, the stable motifs of these reduced networks, and so on (see S1 Fig). We refer to such a diagram as a *stable motif succession diagram*, and we note that it is closely analogous to a cell fate decision diagram. We propose to use this stable motif succession diagram to guide the system to an attractor of interest.

## Results

### Stable motif control implies network control

The stable motifs’ states are partial fixed points of the logical model, and as such, they act as “points of no return” in the dynamics. Normally, the sequence of stable motifs is chosen autonomously by the system based on the initial conditions and timing. We propose to use our knowledge of the sequence of stable motifs to guide the system to an attractor of interest. We refer to this network control method as *stable motif control*.

The basis of the stable motif control approach is that a sequence of motifs from a stable motif succession diagram like Fig 2 uniquely determines an attractor, so controlling each motif in the sequence must prod the system towards this attractor. We give the proof of this statement in Lemma 4 and Proposition 6 of S2 Text section B. The number of nodes that need to be controlled can be minimized by removing motifs that do not need to be controlled and by finding a subset of nodes in a motif which can fix the whole motif’s state. A step by step description of the stable motif control algorithm is given in Methods. For more details on the motif-removal step involved in minimizing the number of control nodes, see S1 Text; for a justification of the steps involved in minimizing the number of control nodes, see S2 Text. S3 Text presents a discussion of the complexity of our methods and mitigation techniques for the most time consuming parts of our methods.

As an example, consider the network in Fig 1(a) and choose 𝒜_2_ in Fig 2 as our target attractor. There are two sequences of stable motifs that lead to 𝒜_2_: ({C = 1, D = 1, E = 0}, {A = 1}) and ({A = 1, B = 1}, {E = 0}). For motif {C = 1, D = 1, E = 0} in the first sequence, fixing E = 0 is enough to fix the whole motif’s state; for motif {A = 1} in the same sequence there is only one node, so the only choice is to fix A = 1. The control set obtained from the first sequence is then {E = 0, A = 1}. For the second sequence, a similar reasoning leads to the same control set, {E = 0, A = 1} (E = 0 from {E = 0}, and A = 1 from {A = 1, B = 1}). The result is a single set of network control interventions for attractor 𝒜_2_, *C*_𝒜_2__ = {{A = 1, E = 0}}. For a step by step description of the stable motif control algorithm applied to this example see S1 Text.

Using our approach with each of the remaining attractors we obtain the following network control interventions: *C*_𝒜_1__ = {{A = 1, E = 1}}, *C*_𝒜_2__ = {{A = 1, E = 0}}, *C*_𝒜_3__ = {{A = 0, E = 1}}, and *C*_𝒜_4__ = {{A = 0, E = 0}}. Inspecting these network control interventions we conclude that controlling nodes A and E is enough to guide the system to each of the four possible attractors, with the exact combination being given by the *C*_𝒜_*i*__’s.

In order to gauge the potential improvement in the control set’s size brought about by our method, we compare our network control set with the feedback vertex set, the subset of nodes whose removal leaves the network without directed cycles. This set was demonstrated to be an effective control target and set an upper limit in the size of the control set in references [12, 13]. Because removing the feedback vertex set from the network must destroy all cycles, including self-loops, there are two possible minimal feedback vertex sets, {A, B, D, E} and {A, C, D, E}. The number of nodes that need to be controlled in our method is half of the size of the feedback vertex set, a substantial improvement. It should be noted that our method does not guarantee that the resulting control sets are small nor that the control sets are the smallest possible, though our case studies suggest that the resulting control sets tend to be relatively small (between one and five nodes out of more than fifty, see Tables 1 and 2, and ref [45]).

**Table 1:** Intervention targets for each control strategy in the T-LGL leukemia network model.

**T-LGL leukemia stable motif control interventions (*C*_*TLGL*_)**
{S1P = ON}, {Ceramide = OFF, SPHK1 = ON}, {Ceramide = OFF, PDGFR = ON}
**Apoptosis stable motif control interventions (*C*_*Apoptosis*_)**
{S1P = OFF}, {PDGFR = OFF}, {SPHK1 = OFF}, {TBET = ON, Ceramide = ON, RAS = ON}
{TBET = ON, Ceramide = ON, GRB2 = ON}, {TBET = ON, Ceramide = ON, IL2RB = ON},
{TBET = ON, Ceramide = ON, IL2RBT = ON}, {TBET = ON, Ceramide = ON, ERK = ON},
{TBET = ON, Ceramide = ON, MEK = ON, PI3K = ON}
**T-LGL leukemia stable motif blocking interventions (*B*_*TLGL*_)**
{S1P = OFF}, {PDGFR = OFF}, {SPHK1 = OFF}, {Ceramide = ON}, {TBET = OFF}, {PI3K = OFF},
{RAS = OFF}, {GRB2 = OFF}, {MEK = OFF}, {ERK = OFF}, {IL2RBT = OFF}, {IL2RB = OFF}
**Apoptosis stable motif blocking interventions (*B*_*Apoptosis*_)**
{S1P = ON}, {PDGFR = ON}, {SPHK1 = ON}, {Ceramide = OFF}, {sFas = ON}, {Fas = OFF},
{TBET = OFF}, {PI3K = OFF}, {RAS = OFF}, {GRB2 = OFF}, {MEK = OFF}, {ERK = OFF},
{IL2RBT = OFF}, {IL2RB = OFF}

**Table 2:** Intervention targets for each control strategy in the helper T cell network.

**Th1 stable motif control interventions (*C*_*Th*1_)**
{TBET = ON}
**Th2 stable motif control interventions (*C*_*Th*2_)**
{GATA3 = ON}
**Th17 stable motif control interventions (*C*_*Th*17_)**
{GATA3 = OFF, FOXP3 = OFF, TBET = OFF, STAT3 = ON},
{GATA3 = OFF, FOXP3 = OFF, TBET = OFF, IL10 = ON},
{GATA3 = OFF, FOXP3 = OFF, TBET = OFF, IL10R = ON},
{GATA3 = OFF, FOXP3 = OFF, TBET = OFF, IL21 = ON},
{GATA3 = OFF, FOXP3 = OFF, TBET = OFF, IL21R = ON},
{GATA3 = OFF, FOXP3 = OFF, TBET = OFF, IL23R = ON, RORGT = ON}
**Treg stable motif control interventions (*C*_*Treg*_)**
{GATA3 = OFF, FOXP3 = ON, TBET = OFF}, {GATA3 = OFF, TBET = OFF, STAT3 = OFF},
{GATA3 = OFF, TBET = OFF, IL23R = OFF, IL10R = OFF, IL21R = OFF},
{GATA3 = OFF, TBET = OFF, IL23R = OFF, IL10 = OFF, IL21R = OFF},
{GATA3 = OFF, TBET = OFF, IL23R = OFF, IL10R = OFF, IL21 = OFF},
{GATA3 = OFF, TBET = OFF, IL23R = OFF, IL10 = OFF, IL21 = OFF}
**Th1 stable motif blocking interventions (*B*_*Th*1_)**
{GATA3 = ON}, {TBET = OFF}, {IL4 = ON}, {IL4R_2 = ON}, {STAT6 = ON}, {STAT1 = OFF},
{IFNG = OFF}, {IFNGR = OFF}, {IL23 = OFF}, {IL10 = ON, OFF}, {IL10R = ON, OFF},
{IL21 = ON, OFF}, {IL21R = ON, OFF}, {STAT3 = ON, OFF}, {IL23R = ON, OFF},
{RORGT = ON, OFF}, {FOXP3 = ON, OFF}
**Th2 stable motif blocking interventions (*B*_*Th*2_)**
{GATA3 = OFF}, {TBET = ON}, {STAT1 = ON}, {IFNG = ON}, {IFNGR = ON}, {IL23 = OFF},
{IL23R = OFF}, {STAT3 = OFF}, {IL10 = OFF}, {IL10R = OFF}, {RORGT = ON},
{FOXP3 = ON, OFF}
**Th17 stable motif blocking interventions (*B*_*Th*17_)**
{GATA3 = ON}, {TBET = ON}, {IL4 = ON}, {IL4R_2 = ON}, {STAT6 = ON}, {STAT1 = ON},
{IFNG = ON}, {IFNGR = ON}, {STAT3 = OFF}, {FOXP3 = ON}, {RORGT = OFF},
{IL21 = OFF}, {IL21R = OFF}, {IL23 = OFF}, {IL23R = OFF}, {IL10 = OFF}, {IL10R = OFF}
**Treg stable motif blocking interventions (*B*_*Treg*_)**
{GATA3 = ON}, {TBET = ON}, {IL4 = ON}, {IL4R_2 = ON}, {STAT6 = ON}, {STAT1 = ON},
{IFNG = ON}, {IFNGR = ON}, {STAT3 = ON, OFF}, {FOXP3 = OFF}, {RORGT = ON, OFF},
{IL21 = ON, OFF}, {IL21R = ON, OFF}, {IL23 = OFF}, {IL23R = ON, OFF}, {IL10 = ON, OFF},
{IL10R = ON, OFF}

### Blocking stable motifs may obstruct specific attractors

In many situations the main interest is to prevent the system from reaching an unwanted state (e.g. the proliferative cell state encountered in tumors). Based on the motif-sequence point of view provided by the stable motif succession diagram (Fig 2), we hypothesize that blocking the stable motifs that lead to an attractor will either prevent or make it less likely for the system to reach this attractor. We refer to this network control method as *stable motif blocking*. The algorithm for the method is given in Methods.

The interventions obtained from this method are negations of node states of the target attractor, and as such, have the property of eliminating the intended attractor. However, new attractors can arise that are similar to the destroyed attractor. In biological situations (like in our test cases) one commonly has certain molecular markers of cell fate which specify the attractor to a large degree but not at the level of every node. Thus the final state obtained after stable motif blocking may still be consistent with the biological specification of the undesired attractor, making the intervention unsuccessful. We also adopt a stricter definition for a successful intervention: if a long-term but not permanent intervention (i.e. a transient intervention) reduces the number of network states or trajectories that lead to the unwanted attractor, then the intervention is considered to be *long-term successful*. The best-case scenario would be that the manipulated network has only the desired attractors of the original network (i.e., any but the unwanted attractors), in which case the network will stay in these attractors even if the intervention is stopped.

Consider, for example, the network in Fig 1(a) and the attractor 𝒜_3_ in Fig 2. From the stable motif succession diagram (Fig 2), the stable motifs involved in the sequences that lead to 𝒜_3_ are {A = 0}, {D = 1}, and {E = 1}. Our approach proposes blocking these motifs to obstruct the system from reaching 𝒜_3_, that is, it provides ℬ_𝒜_3__ = {{A = 1}, {E = 0}, {D = 0}} or a combination of these node states as intervention candidates.

To verify the effectiveness of the interventions, we analyze the dynamics of the manipulated network with each individual intervention. The first intervention (A = 1) causes the system to have 𝒜_1_ and 𝒜_2_ as its only attractors, and thus, the network is driven towards these attractors and away from the unwanted attractor 𝒜_3_. Furthermore, the network stays in those attractors even after the intervention is stopped, as they are also attractors of the original network, so the intervention is long-term successful. Similarly, the second intervention (E = 0) causes the system to have 𝒜_2_ and 𝒜_4_ as its sole attractors, so it is also a long-term successful intervention. The third intervention (D = 0) only leaves attractor 𝒜_1_ intact, and also gives rise to two new attractors. To evaluate if this intervention is long-term successful we compare the probabilities that an arbitrary initial condition ends in 𝒜_3_ with and without the intervention. For the intervened case, we set D = 0 for a long time, then stop the intervention and wait for the network to reach an attractor. We find that the intervention makes it more likely for an arbitrary initial condition to reach 𝒜_3_, so this intervention is not long-term successful.

### Verification of the method’s effectiveness in test cases

The network control framework we propose is applicable to any cell fate reprogramming process for which a logical dynamical model can be constructed. This is a broad and increasing domain of application: refs. [24–28] are examples of recent logical models that had experimentally validated predictions, while other examples can be found in the review articles [29, 30].

To demonstrate the potential of our framework, we choose two types of cell fate reprogramming processes: disease therapeutics and cell differentiation. More specifically, we use our network control framework to predict network control interventions on previously developed logical dynamic models for a leukemia signaling network and for the network controlling the differentiation of helper T cells. We confirm the effectiveness of the predicted stable motif control interventions using dynamic simulations, an independent verification of the result we prove in S2 Text. For the case of stable motif blocking interventions, whose effectiveness is not guaranteed, we use dynamic simulations to test the effectiveness of the predicted interventions.

#### T cell large granular lymphocyte leukemia network

Cytotoxic T cells are a central part of the immune system’s response to infection. These T cells detect antigens in infected cells and, in response, induce the self-destruction of the infected cells. After fighting infection normal cytotoxic T cells undergo activation-induced cell death (apoptosis), but in T-cell large granular lymphocyte (T-LGL) leukemia cytotoxic T cells avoid cell death and survive, which eventually leads to diseases such as autoimmune disorders.

**Fig 3:**
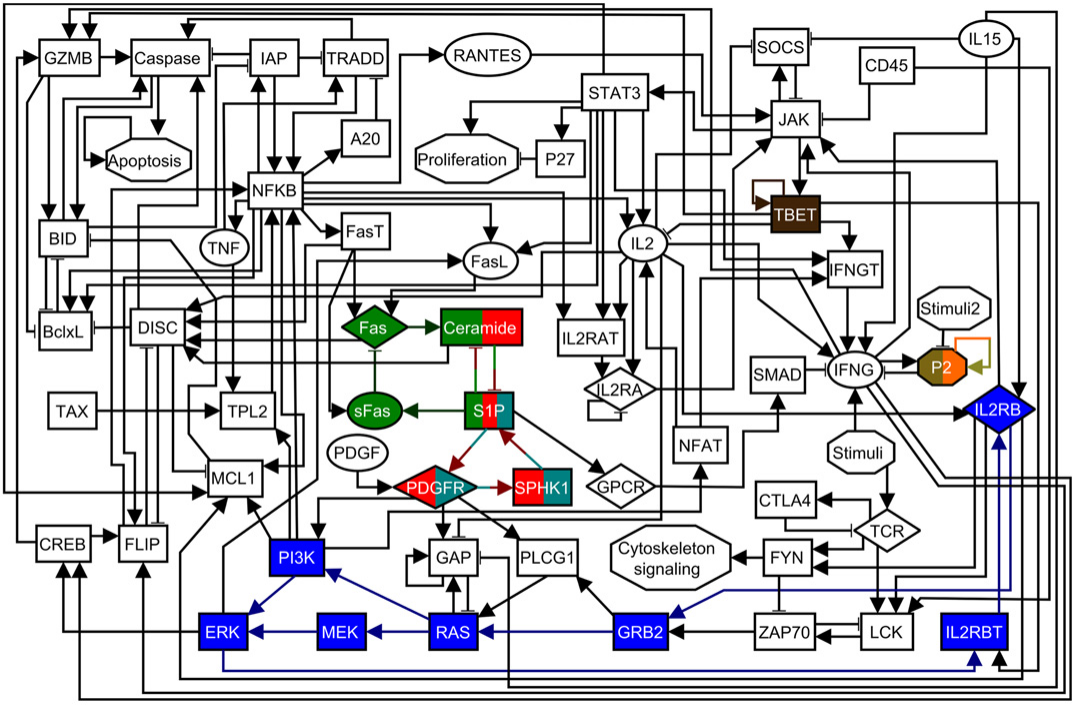
The T-LGL leukemia survival signaling network. The shape of the nodes indicates the cellular location or the type of nodes: rectangles indicate intracellular components, ellipses indicate extracellular components, diamonds indicate receptors, and hexagons represent conceptual nodes (Stimuli, Stimuli2, P2, Cytoskeleton signaling, Proliferation, and Apoptosis). Node colors are used to denote the different stable motifs of the network in the presence of the external signals Stimuli and IL15. Nodes and edges with multiple colors are part of several stable motifs. An arrowhead or a short perpendicular bar at the end of an edge indicates activation or inhibition, respectively. This figure and its caption are adapted from [46].

A Boolean network model of cytotoxic T cell signaling that reproduces the known experimental results of these T cells in the context of T-LGL leukemia was previously constructed by Zhang et al. [28]. This network model consists of 60 nodes and 142 regulatory edges, with the nodes representing genes, proteins, receptors, small molecules, external signals (e.g. Stimuli), or biological functions (e.g. Apoptosis). The T-LGL network is shown in Fig 3 and its logical functions are reproduced in S4 Text. Previous work by Zhang et al. [28] and Saadatpour et al. [46] has shown that in the sustained presence of the external signals IL15, PDGF, and Stimuli (antigen presentation) the system has two attractors: one that recapitulates the survival phenotype and node deregulations seen in T-LGL leukemia, and a second one that corresponds to self-programmed cell death (apoptosis) (see S4 Text for more details about attractor specification).

**Fig 4:**
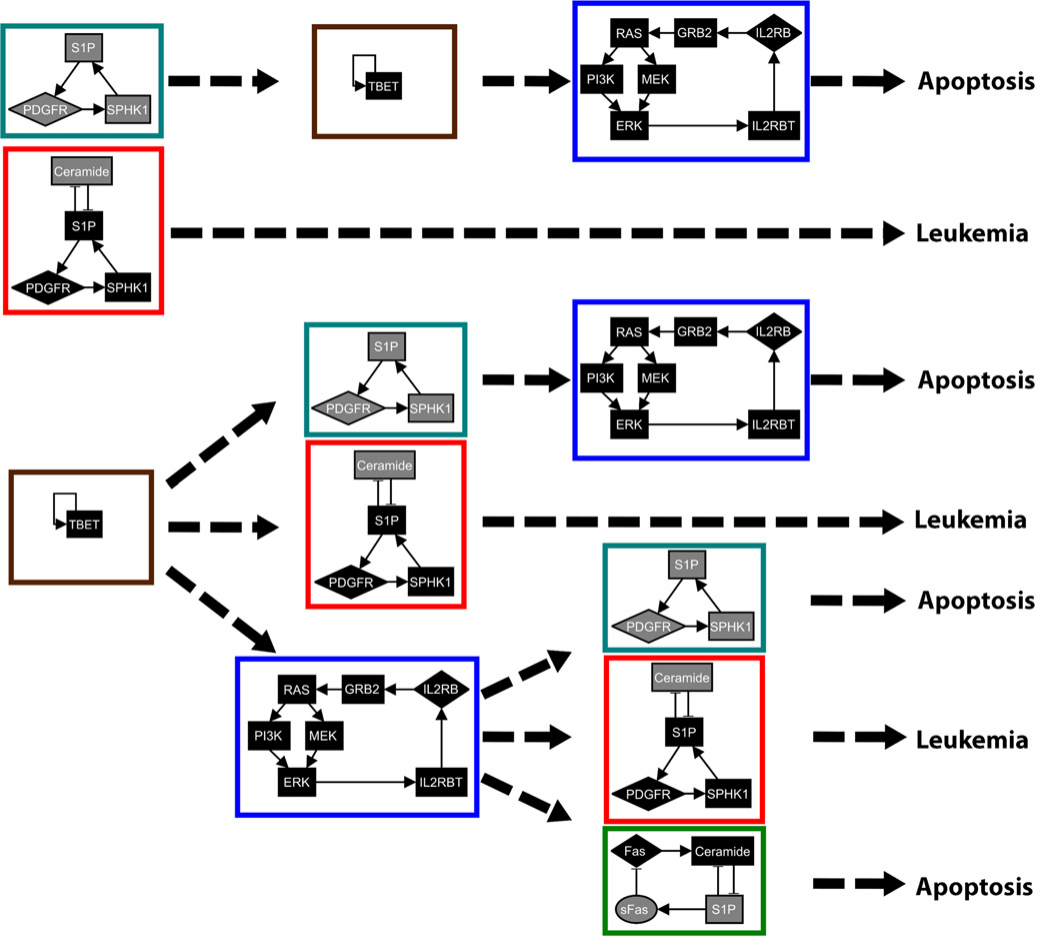
Stable motif succession diagram for the T-LGL leukemia network. The color of the nodes denotes their respective node states in the stable motifs: gray for 0 and black for 1. The colored rectangle surrounding each stable motif corresponds to the respective color of the motif in Fig 3. There are two possible attractors for the system: the normal state of self-programmed cell death (apotosis) and the diseased state (T-LGL leukemia). The attractors obtained for each possible sequence of stable motifs are shown in the rightmost part of the figure.

We first use our attractor-finding method on the T-LGL leukemia network in the presence of the external signals Stimuli and IL15 to obtain the stable motifs and the succession diagram. The result is 7 different stable motifs, each of which is shown in Fig 3 with a different node/edge color (nodes and edges with multiple colors are part of several stable motifs). The stable motif succession diagram for the T-LGL network is shown in Fig 4. For simplicity we do not include the motifs associated with the node P2 in the succession diagram, as these motifs require the other stable motifs to influence the resulting attractor in the succession diagram.

The succession diagram in Fig 4 suggests a simple picture for the cell fate determination process: the activation of any of the three S1P-related motifs is enough to drive the system to either apoptosis (either the teal or the green stable motif in Figs. 3 and 4) or T-LGL leukemia (the red stable motif in Figs. 3 and 4). This result agrees with previous studies of T-LGL leukemia, in which it was found that blocking S1P signaling induced apoptosis in leukemic T-LGL cells [28, 47], a result reproduced by the network model when the state of S1P was set to OFF [41, 46].

Next, we use the stable motif diagram in Fig 4 and our two control strategies to find intervention targets for the T-LGL leukemia network. The obtained intervention targets for each control strategy are shown in Table 1. Note that some intervention targets may be present in both control strategies (e.g. {S1P = OFF} is a target both for apoptosis control and T-LGL attractor blocking). For the case of stable motif blocking one may have the same intervention for blocking two different attractors (e.g. {TBET = OFF}), which means that this intervention could block either attractor.

To validate an intervention target, we compare the probabilities that an arbitrary initial condition ends in the target attractor with and without the intervention (see Methods). The results of the intervention target validation are summarized in S1 Table. For all the stable motif control interventions we obtain 100% effectiveness in reaching the desired state, both for the case in which the intervention is permanent and for the case in which it is not. This means that all stable motif control interventions are *long-term successful*, in agreement with our formal results in S2 Text. For example, when fixing S1P = OFF the apoptosis attractor is reached for all the initial conditions, indicating that the T-LGL attractor is unreachable. For the case of the stable motif blocking interventions we find that each of them but one (GRB2 = OFF) is successful in blocking its target attractor or one of its target attractors, though not always with 100% effectiveness. For example, for TBET = OFF the apoptosis attractor is reached from 10% of the initial conditions, which is a substantial reduction from the baseline of 62% in the case of no intervention, indicating that this interventions is effective as an apoptosis blocking strategy. We also find that most of the stable motif blocking interventions are effective when the intervention is permanent, but only a few of them are effective when the intervention is temporary.

Single interventions are the most commonly used therapeutic strategies for treating diseases. Thus, we evaluate the success of each single intervention from control sets with more than one node (see S1 Table). We find that one of the 12 single node interventions, Ceramide = ON, is 100% effective and long-term successful. Of the remaining 11 single node interventions only a few are successful (Ceramide = OFF, SPHK1 = ON, and PDGFR = ON) and/or long-term successful (SPHK1 = ON and PDGFR = ON) but none of them are 100% effective. This result illustrates the benefit of combinatorial interventions over single interventions.

#### Helper T cell differentiation network

Helper T cells are crucial in the regulation of the immune response in mammals. These T cells release specific cytokines that alter how the immune system responds to external agents, for example, by recruiting specific immune system cells to fight infection, promoting antibody production, or inhibiting the activation and proliferation of other cells. Various subtypes of helper T cells are known, such as Th1, Th2, Th17 and Treg, which are distinguished by a differential expression of specific transcription factors and cytokines.

**Fig 5:**
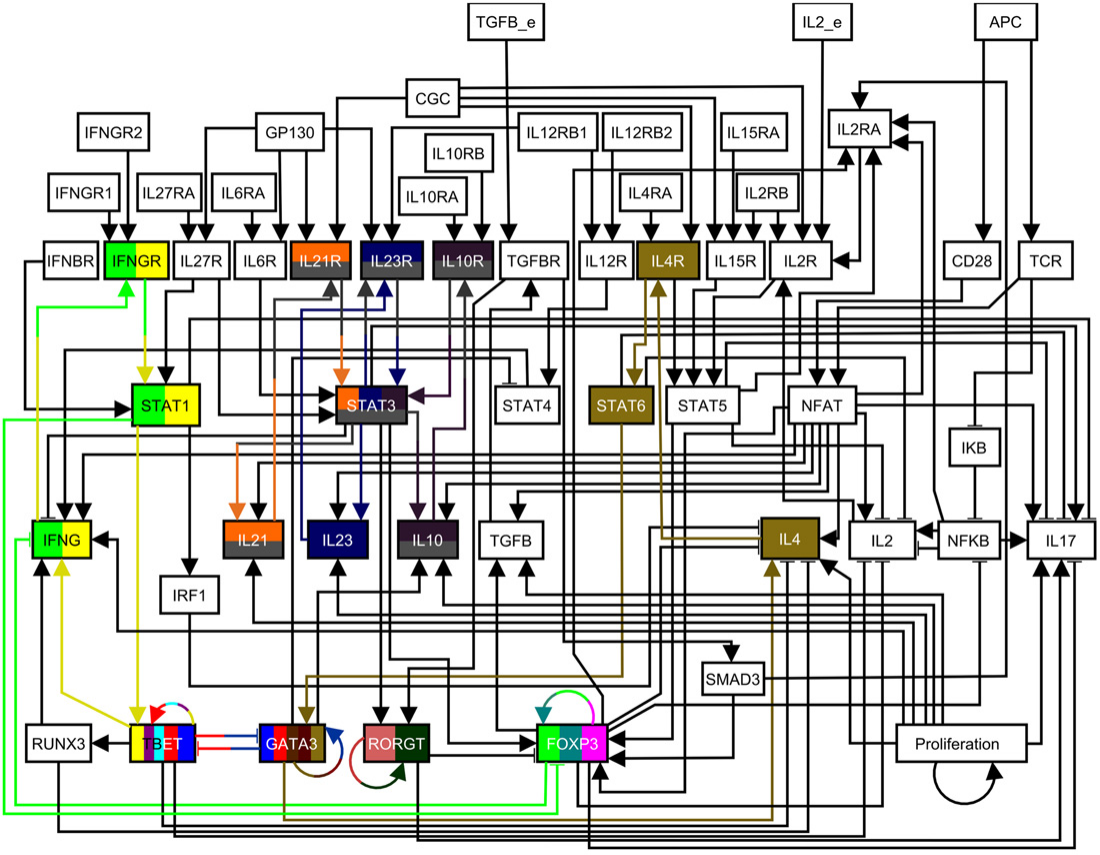
The helper T cell differentiation network. The nodes that encode the environmental conditions (APC = ON, TGFB_e = ON, IL2_e = ON) are located in the upper part of the network diagram. Node colors are used to denote the different stable motifs of the network in the used environmental conditions. Nodes and edges with multiple colors are part of several stable motifs. An arrowhead or a short perpendicular bar at the end of an edge indicates activation or inhibition, respectively. This figure is adapted from [48].

A logical network model of the regulatory and signaling pathways controlling helper T cell activation and differentiation was constructed by Naldi et al. [48]. This network model has several attractors, which correspond to the known canonical helper T cell subtypes, and also to some hybrid cell types (see [48] and S5 Text). The reachability of each attractor depends on the presence of several external environmental signals (either cytokines or antigen), which are represented as input nodes in the network. For our study we use one of the environmental conditions studied by Naldi et al. (TGFB_e = ON, IL2_e = ON, and APC = ON) [48] because it allows us to explore control targets for all T cell subtypes. The helper T cell differentiation network under the selected environmental conditions consists of 55 nodes and 121 edges and is shown in Fig 5. Its corresponding logical functions are reproduced in S5 Text.

**Fig 6:**
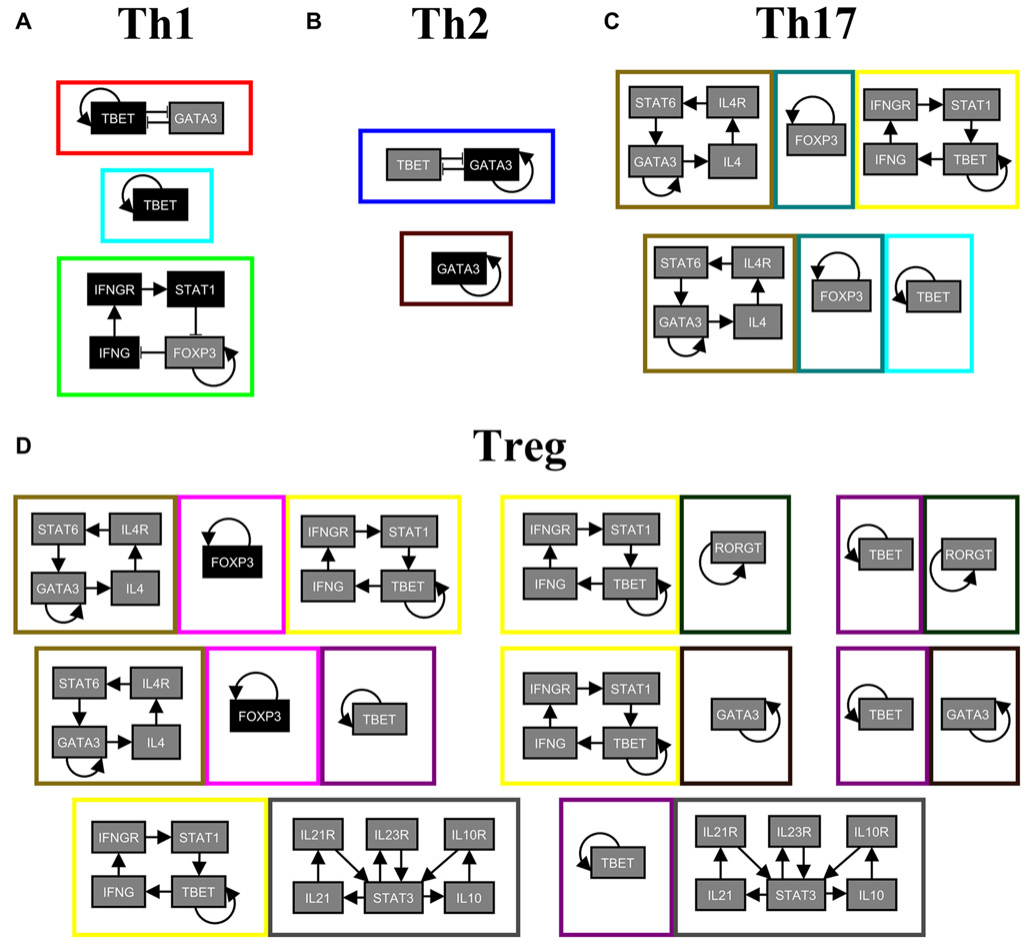
Minimal subsets of stable motifs associated to each helper T cell subtype. Each stable motif is enclosed by a colored rectangle, and motifs which are part of the same minimal subset have their enclosing rectangles touching each other. The node colors denotes their respective node states in the stable motifs: gray for 0 and black for 1. The color of the rectangle enclosing each stable motif corresponds to the respective color of that motif in Fig 5.

We obtain 17 stable motifs, each of which is shown in Fig 5 with a different node/edge color, and a stable motif succession diagram composed of 697 sequences. Despite the large size of the succession diagram, a closer look at it gives a simple interpretation: the stable motifs associated with each attractor regulate the characteristic transcription factor of each helper T cell subtype. To check this, we look at the minimal subsets of stable motifs that are sufficient for a sequence to lead to a single differentiated helper T cell subtype (see Fig 6 and S5 Text). We use the stable motif succession diagram and our stable motif control and stable motif blocking strategies to find intervention targets for each helper T cell subtype (see Table 2).

To validate the proposed intervention targets we use the same procedure as in the T-LGL leukemia network case (see Methods). We also look at the effect of single node interventions for control sets with more than one node. The results of the intervention targets for the stable motif control, stable motif blocking strategies, and single node interventions are summarized in S2 Table. We find that (i) there is a 100% effectiveness in reaching the desired state for all the stable motif control interventions, (ii) most of the stable motif blocking interventions are successful in blocking their target attractor or one of their target attractors, though not always with 100% effectiveness, and (iii) some single interventions are successful, but none of them are 100% effective.

### The control targets transcend the logical modeling framework

The network control approach we propose is formulated in a Boolean framework, which brings up the question of whether the control targets identified are dependent on the logical modeling scheme. To address this, we translate the studied Boolean network models into ordinary differential equation (ODE) models using the method described by Wittmann et al. [49]. In the ODE models the node state variables ${\tilde{\sigma }}_{i}$ can take values in the range [0, 1]; the differential equations of the translated model have the form ${\dot{\tilde{\sigma }}}_{i}=(1/\tau_{i})[{\tilde{f}}_{i}({\tilde{\sigma }}_{i_{1}},\ldots ,{\tilde{\sigma }}_{i_{k_{i}}})-{\tilde{\sigma }}_{i}]$, where ${\tilde{f}}_{i}$ is a smooth Hill-type function parameterized by Hill coefficients and threshold parameters, and *τ*_*i*_ is a time-scale parameter. The function ${\tilde{f}}_{i}$ is such that it matches the Boolean function *f*_*i*_ whenever its inputs ${\tilde{\sigma }}_{i_{1}},\ldots ,{\tilde{\sigma }}_{i_{k_{i}}}$ are either 0 or 1. Thus, the fixed point attractors of the Boolean model are preserved in the ODE model.

We test the effectiveness of the stable motif control interventions in the translated ODE models by comparing the probability for an uniformly chosen initial condition to reach the target attractor with and without the intervention (see S6 Text). We find that the stable motif control interventions are still 100% effective or very close for both permanent and transient interventions (S3 Table and S4 Table). We also find that the effectiveness of the interventions is mostly unchanged by varying the Hill coefficients (S5 Table), varying the the time-scale parameters *τ*_*i*_ and thresholds (S6 Table), or fixing the intervened node variables close to but not exactly at the intervention-prescribed values (S7 Table). We finally test single interventions and find that they still underperform combinatorial interventions (S3 Table and S4 Table).

To further validate the successful control targets we identified, we searched the literature for experimental support for these targets. We find that several of the single interventions predicted to be successful in inducing apoptosis of leukemic T cells or in inducing specific T cell types were found to be successful experimentally. The control targets for which experimental support was found, the attractors they lead to, and the references are shown in Table 3. Collectively, these results strongly suggest that the control targets identified by our approach transcend the logical framework.

**Table 3:** Experimental support for successful control targets in Tables 1 and 2.

Intervention	Target attractor	Reference
**T-LGL leukemia**		
{S1P = OFF}	Apoptosis	[47]
{SPHK1 = OFF}	Apoptosis	[28]
{PDGFR = OFF}	Apoptosis	[28, 59]
{Ceramide = ON}	Apoptosis	[60]
{RAS = OFF}	Apoptosis	[61]
{MEK = OFF}	Apoptosis	[61]
{ERK = OFF}	Apoptosis	[61]
{PI3K = OFF}	Apoptosis	[59, 62]
**Helper T cell differentiation**		
{TBET = ON}	Th1	[63, 64]
{GATA3 = ON}	Th2	[63, 65]
{IL21 = ON}	Th17	[66]
{IL21R = ON}	Th17	[66]
{IL23R = ON}	Th17	[66]
{FOXP3 = ON}	Treg	[67]

## Discussion

Identifying control targets for intracellular networks is of crucial importance for practical applications such as disease treatment and stem cell reprogramming. Despite recent advances in network controllability approaches, most of them rely solely on the topology [7, 9, 10, 12, 13] or the dynamics [11, 20–22] of the network. Thus, potentially important effects that depend on the interplay between structure (topology) and function (dynamics), such as combinatorial interactions, are not considered. In this work we proposed a network control approach that combines the structural and functional information of a discrete (logical) dynamic network model to identify control targets. The method builds on the concept of stable motif and its relation to finding attractors [41], and takes it much further by connecting stable motifs with a way to identify targets whose manipulation (upregulation or downregulation) ensures the convergence of the system to an attractor of interest. We illustrated our method’s potential to find intervention targets for cancer treatment and cell differentiation by applying it to network models of T-LGL leukemia and helper T cell differentiation.

The control interventions identified by our method have many desirable characteristics. For example, stable motif control interventions are guaranteed to drive an initial state to the target attractor state with 100% effectiveness, regardless of the initial state, a general result which we prove in S2 Text and corroborate in our test cases (see S1 Table and S2 Table). They are also long-term successful, meaning that the intervention only needs to be applied transiently for the network to reach and stay in the desired state, a general result which we also verify in our test cases (see S1 Table and S2 Table). We attribute these properties to the use of the natural (autonomous) dynamics of the network to control its dynamics.

Another noteworthy characteristic of our stable motif control method is the combinatorial nature of the multi-target interventions. As shown in S1 Table and S2 Table, only one single-node intervention (namely, Ceramide = ON in the T-LGL leukemia network) was able to match the 100% effectiveness of the multi-target interventions. This agrees with recent clinical studies on the advantages of combinatorial over single target interventions [50–52]. Finally, the stable motif control interventions for our case studies target only a few nodes (between one and five out of more than fifty), which matches what is expected from stem cell reprogramming experiments [1–3, 8].

The framework presented in this work is formulated and applied in the context of logical network modeling of cell fate reprogramming processes but its applicability is not restricted to it. Indeed, our control approach is applicable to any dynamic process that can be captured qualitatively by a Boolean dynamic network model such as ecological community dynamics [53], social dynamics [54, 55], or disease spreading [56, 57]. The validity of the control targets on the translated ODE models of our two case studies and the experimental support found for several of these targets demonstrates the broader, potentially model-independent reach of our method. Further work is needed to address exactly how to extend the concept of stable motif and our network control approach to continuous models; formalizing our framework to admit an arbitrary number of discrete states and other updating schemes may prove a valuable step in this direction.

Taken together, our results provide a novel framework for the control of the dynamics of intracellular networks that combines realistically obtainable structural and functional information of the network of interest. As such, we expect this framework to be significant to a variety of practical applications and to also provide a new avenue to better understand how the complex behaviors of cells in living organisms emerges from the underlying network of biochemical interactions.

## Methods

### Computational methods

The simulations of the logical model were done with the BooleanDynamicModeling Java library, while the attractor-finding method and the analysis of the stable motif succession diagrams were performed using the StableMotifs Java library, both of which are freely available on GitHub (on http://github.com/jgtz/BooleanDynamicModeling/ and http://github.com/jgtz/StableMotifs/, respectively). The source code of a Java project that allows the user to reproduce the stable motif succession diagrams and control sets for the test cases analyzed is also freely available on GitHub under the examples folder of the StableMotifs Java library. The generation of the ODE model from the logical model was done using the MATLAB implementation of the method of Wittman et al. [49, 58]; the numerical integration of the ODE models was performed using MATLAB’s ode45 function (see S6 Text for more details). The networks in all figures were created using the yEd graph editor (http://www.yworks.com/).

### General asynchronous updating scheme

In the general asynchronous scheme, the state of the nodes is updated at discrete time steps starting from an initial condition at *t* = 0. At every time step, one of the variables is chosen randomly (uniformly) and is updated using its respective function and the state of its regulators at the previous time step

$$\sigma_{j}\left(t+1\right)=f_{j}(\sigma_{j_{1}}\left(t\right),\sigma_{j_{2}}\left(t\right),\cdots ,\sigma_{j_{k_{j}}}\left(t\right)),$$ (1)

while the rest of the variables retain their state. In this way, every possible update order is allowed, and thus, all relative timescales of the processes involved are sampled.

### Stable motif control algorithm

For an attractor of interest 𝒜, the steps of the stable motif network control method are the following:

- *Step 1*: Identify the sequences of stable motifs that lead to 𝒜. These can be obtained from the stable motif succession diagram (see Fig 2) by choosing the attractor of interest in the right-most part and selecting all of the attractor’s predecessors in the succession diagram.
- *Step 2*: Shorten each sequence 𝒮 by identifying the minimum number of motifs in 𝒮 required for reaching 𝒜 and removing the remaining motifs from the sequence. This minimum number of motifs can be identified from the stable motif succession diagram (Fig 2); they are the motifs after which all consequent motif choices lead to the same attractor 𝒜.
- *Step 3*: For each stable motif’s state ℳ = (*σ*_*m*_1__,*σ*_*m*_2__,…,*σ*_*m*_*l*__), find the subsets of stable motif’s states *O* = {*M*_*i*_},*M*_*i*_ ⊆ ℳ that, when fixed in the logical model, are enough to force the state of every node in the motif into ℳ. At worst, there will only be one subset, which will equal the whole stable motif’s state ℳ. If any of these subsets is fully contained in another subset, remove the larger of the subsets. In each stable motif sequence 𝒮 = (ℳ_1_,…,ℳ_*L*_), substitute every stable motif ℳ_*j*_ with the subsets of the stable motif’s states obtained, that is, 𝒮 = (*O*_1_,…,*O*_*L*_).
- *Step 4*: For each sequence 𝒮 = (*O*_1_,…,*O*_*L*_) create a set of states 𝒞 by choosing one of the subsets of stable motif’s states *M*_*k*_*j*__ in each *O*_*j*_ and taking their union, that is, 𝒞 = *M*_*k*_1__∪⋯∪*M*_*k*_*L*__,*M*_*k*_*j*__ ∈ *O*_*j*_. The network control set for attractor 𝒜 is the set of node states *C*_𝒜_ = {𝒞_*i*_} obtained from all possible combinations of subsets of stable motif’s states *M*_*k*_*j*__’s for every sequence 𝒮. To avoid any redundancy, we additionally prune *C*_𝒜_ of duplicates and remove each set of node states 𝒞_*i*_ which is a superset of any of the other sets of node states 𝒞_*j*_ (i.e. 𝒞_*j*_ ⊂ 𝒞_*i*_).

For a pseudocode of each step of the stable motif control algorithm see S7 Text.

### Stable motif blocking algorithm

Given an attractor 𝒜 one is interested in obstructing, the steps to identify potential interventions are the following:

- *Step 1*: Identify the sequences of stable motifs that lead to 𝒜. This step is the same as the first step in the stable motif control algorithm, and can be obtained from the stable motif succession diagram (Fig 2).
- *Step 2*: Take each stable motif’s state ℳ_*i*_ in the sequences obtained in the previous step. Create a new set **M**_𝒜_ with all of these stable motif states, **M**_𝒜_ = {ℳ_*i*_}.
- *Step 3*: Take each node state *σ*_*j*_ ⊂ ℳ_*i*_ of the stable motif’s states ℳ_*i*_ in **M**_𝒜_. Create a new set ℬ_𝒜_ with the negation of each node state, ${ℬ}_{𝒜}=\left\{{\bar{\sigma }}_{j}\right\}$. The node states in ℬ_𝒜_ and any combination of them are identified as potential interventions to block attractor 𝒜.

For a pseudocode of each step of the stable motif blocking algorithm see S7 Text.

### Intervention target validation

To validate an intervention target, we fix the node states prescribed by the intervention, choose a random (uniformly chosen) initial condition, and evolve the system using the general asynchronous updating scheme for a sufficiently large number of time steps so that the system reaches an attractor. We find that, for our test cases, temporal evolution for 10,000 time steps ensures reaching an attractor from any initial condition considered with stable motif control intervention or without an intervention; to be safe, we choose to evolve for 50,000 time steps in all cases. We repeat this for a large number of initial conditions (100,000) and calculate the probability of reaching each attractor from an arbitrary (uniformly chosen) initial condition. We also look at the probability of reaching each attractor when the intervention is not permanent (i.e. it is transient), that is, we fix the prescribed node states for a large number of time steps, then stop fixing these states and wait for another large number of time steps for the system to reach an attractor. For our test cases, we find that using 10,000 time steps for each evolution stage (with and then without prescribed node states) is enough to preserve the first three digits of the estimated probabilities *p*_*Attr*_ of reaching the attractor of interest, consistent with what is expected from the standard deviation of the estimated probability *p*_*Attr*_. To be safe, we choose to evolve for 50,000 time steps for each evolution stage.

The number of initial conditions we use is chosen to give three significant figures in the estimated probabilities *p*_*Attr*_. For our test cases, we find that 100,000 initial conditions are enough to estimate the probabilities *p*_*Attr*_ of reaching the attractor of interest with an error (standard deviation of the estimated probability *p*_*Attr*_) of 3⋅10^−3^[*p*_*Attr*_(1−*p*_*Attr*_)]^1/2^. Equivalently, if *p*_*Attr*_ is expressed as a percentage (which we denote as %*p*_*Attr*_ for clarity), the error in it is estimated as 3⋅10^−3^[%*p*_*Attr*_(100%−%*p*_*Attr*_)]^1/2^% (e.g. 0.03% for a %*p*_*Attr*_ of 1%, and 0.15% for a %*p*_*Attr*_ of 50%). The number of time steps we use is enough to show no changes in *p*_*Attr*_ beyond what is expected from the standard deviation of the estimated probability *p*_*Attr*_, and is also found to be enough for the initial conditions to reach the attractors when no interventions are applied.

## Supporting Information

S1 Text Details and examples of the attractor finding method and stable motif control algorithm. (PDF)

S2 Text Mathematical foundations of the attractor-finding method and of the stable motif control approach. (PDF)

S3 Text Time complexity and mitigation techniques for the attractor-finding method and the stable motif control approach. (PDF)

S4 Text Logical rules and classification of attractors in the T-LGL leukemia network model. (PDF)

S5 Text Logical rules, classification of attractors, and analysis of the stable motif succession diagram in the helper T cell differentiation network model. (PDF)

S6 Text Translating the logical network models into ordinary differential equation models, and intervention target validation for the ordinary differential equation models. (PDF)

S7 Text Pseudocode for the stable motif control algorithm and the stable motif blocking algorithm. (PDF)

S8 Text List of references that appear in the supporting information files. (PDF)

S1 Fig Stable motifs and simplified logical networks for the logical network in Fig 1. Read from left to right, the figure shows the logical network in Fig 1, the stable motifs of this logical network, the simplified networks obtained from tracing the downstream effect of each of the original logical network’s stable motifs, and the stable motifs obtained from these simplified networks. Nodes are colored based on their respective node state: gray for 0, black for 1, and white for nodes whose state is not yet determined. Each large arrow has an associated stable motif sharing the arrow’s color. These large arrows stand for the use of a network reduction technique on the network they start from by tracing the downstream effect of their associated stable motifs on this network.
(PDF)

S2 Fig Example of the expanded network representation of selected nodes of the logical network in Fig 1. The logical function of each example node is shown above its expanded network representation. Nodes are colored white if they denote normal nodes or complementary node (complementary nodes have a bar above their name, while normal nodes do not), and colored black if they denote composite nodes. For more details see S1 Text and S2 Text. (a) Expanded network representation for normal node *C*, complementary node $\bar{C}$, and their inputs. (b) Expanded network representation for normal node *B*, complementary node $\bar{B}$, and their inputs.
(PDF)

S3 FigExample logical network displaying unstable oscillations.The figure shows (a) a two node Boolean network whose logical functions are given by an XOR function, (b) the network’s state transition graph, i.e., all combinations of network states and the allowed transitions between them under the general asynchronous updating scheme, and (c) the network’s stable motif succession diagram. This Boolean network is the simplest example (up to a relabeling of node states) of so-called unstable oscillations. Unstable oscillations refer to a subset of nodes whose node states oscillate in an attractor while their node states are fixed in a different attractor, even though both attractors are the same except for the state of this subset of nodes. In the example Boolean network shown in this figure, we have the states of nodes *A* and *B* oscillate between three network states in attractor 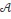 ^′^ = {(*A* = 1,*B* = 0), (*A* = 0,*B* = 0), (*A* = 0,*B* = 1)}, while they are fixed in attractor 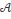 = {*A* = 1,*B* = 1}. Unstable oscillations are treated with special care when using our attractor-finding method, since ignoring them can lead to missing attractors displaying this behavior; for more details see S1 Text and S2 Text.(PDF)

S4 FigExample logical network displaying incomplete oscillations.The figure shows (a) a three node Boolean network that displays incomplete oscillations, (b) the sub-state-space of nodes *A* and *B* in the network’s state transition graph (i.e., all combinations of network states and the allowed transitions between them) under the general asynchronous updating scheme, and (c) the network’s stable motif succession diagram. Incomplete oscillations refer to a subset of nodes whose node states oscillate in an attractor but do not visit all possible states of their sub-state-space in the attractor. In the example Boolean network shown in this figure, we have the states of nodes *A* and *B* oscillate between three subnetwork states {(*A* = 1,*B* = 0), (*A* = 0,*B* = 0), (*A* = 0,*B* = 1)} in the attractors 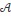 and 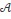 ^′^. Incomplete oscillations are treated with special care when using our attractor-finding method, since ignoring them can lead to missing attractors displaying this behavior; for more details see S1 Text and S2 Text.(PDF)

S5 Fig Expanded network representation of the stable motifs of the logical network in Fig 1, and the terms in the logical functions associated to each stable motif. Read from left to right, the figure shows the stable motifs of the logical network in Fig 1, the expanded network representation of the stable motifs (from which stable motifs are formally defined), and the terms of the logical function associated to each stable motif. For more details on the expanded network representation see S1 Text and S2 Text.
(PDF)

S1 Table Validation of the intervention targets in Table 1 and single interventions from control sets with more than one node in Table 1 for the T-LGL leukemia network model. The relative apoptosis % change is defined as (Apoptosis %−Normal apoptosis %)/(Normal apoptosis %), where Normal apoptosis % = 62.1% is the percentage of initial conditions that go to apoptosis when no intervention is applied. Interventions marked with † appear in more than one control strategy or target attractor in Table 1. The percentages are significant in the digits shown and have an estimated absolute error (standard deviation of the mean) of 3⋅10^−3^[%*p*_*Attr*_(100%−%*p*_*Attr*_)]^1/2^ %, where %*p*_*Attr*_ is the percentage shown (e.g. 0.03% for a %*p*_*Attr*_ of 1%, and 0.15% for a %*p*_*Attr*_ of 50%).
(PDF)

S2 Table Validation of the intervention targets in Table 2 and single interventions from control sets with more than one node in Table 2 for the helper T cell network. The relative attractor % change is defined as (attractor %−normal attractor %)/(normal attractor %), where the normal attractor % is the percentage of initial conditions that go to the attractor of interest when no intervention is applied. The normal attractor percentages are 48.6%, 47.5%, 1.3%, and 2.6% for the Th1, Th2, Th17, and Treg helper T cell subtypes, respectively. Interventions marked with † appear in more than one control strategy or target attractor in Table 2. The percentages are significant in the digits shown and have an estimated absolute error (standard deviation of the mean) of 3⋅10^−3^[%*p*_*Attr*_(100%−%*p*_*Attr*_)]^1/2^ %, where %*p*_*Attr*_ is the percentage shown (e.g. 0.03% for a %*p*_*Attr*_ of 1%, and 0.15% for a %*p*_*Attr*_ of 50%).
(PDF)

S3 Table Validation of the intervention targets in Table 1 for the T-LGL leukemia differential equation network model and single interventions from control sets with more than one node in Table 1 for the T-LGL leukemia differential equation network model. The relative apoptosis % change is defined as (Apoptosis %−Normal apoptosis %)/(Normal apoptosis %), where Normal apoptosis % = 54.7% is the percentage of initial conditions that go to apoptosis when no intervention is applied. Interventions marked with † appear in more than one control strategy or target attractor in Table 1. The percentages are significant in the digits shown and have an estimated absolute error (standard deviation of the mean) of 6⋅10^−3^[%*p*_*Attr*_(100%−%*p*_*Attr*_)]^1/2^ %, where %*p*_*Attr*_ is the percentage shown (e.g. 0.06% for a %*p*_*Attr*_ of 1%, and 0.3% for a %*p*_*Attr*_ of 50%).
(PDF)

S4 Table Validation of the stable motif control intervention targets in Table 2 for the helper T cell differential equation network model. The relative attractor % change is defined as (attractor %−normal attractor %)/(normal attractor %), where the normal attractor % is the percentage of initial conditions that go to the attractor of interest when no intervention is applied. The normal attractor percentages are 50.0%, 45.4%, 2.8%, and 1.8% for the Th1, Th2, Th17, and Treg helper T cell subtypes, respectively. Interventions marked with † appear in more than one control strategy or target attractor in Table 2. The percentages are significant in the digits shown and have an estimated absolute error (standard deviation of the mean) of 6⋅10^−3^[%*p*_*Attr*_(100%−%*p*_*Attr*_)]^1/2^ %, where %*p*_*Attr*_ is the percentage shown (e.g. 0.06% for a %*p*_*Attr*_ of 1%, and 0.3% for a %*p*_*Attr*_ of 50%).
(PDF)

S5 Table Validation of some stable motif control intervention targets in Table 1 for different Hill coefficients (*n*) in the T-LGL leukemia differential equation network model. The percentages are significant in the digits shown and have an estimated absolute error (standard deviation of the mean) of 6⋅10^−3^[%*p*_*Attr*_(100%−%*p*_*Attr*_)]^1/2^ %, where %*p*_*Attr*_ is the percentage shown (e.g. 0.06% for a %*p*_*Attr*_ of 1%, and 0.3% for a %*p*_*Attr*_ of 50%).
(PDF)

S6 Table Validation of some stable motif control intervention targets in Table 1 for different Hill coefficients (*n*) in the T-LGL leukemia differential equation network model with randomly chosen *τ*_*i*_ and *θ*_*i*_. The percentages are significant in the digits shown and have an estimated absolute error (standard deviation of the mean) of 5⋅10^−3^[%*p*_*Attr*_(100%−%*p*_*Attr*_)]^1/2^ %, where %*p*_*Attr*_ is the percentage shown (e.g. 0.05% for a %*p*_*Attr*_ of 1%, and 0.25% for a %*p*_*Attr*_ of 50%).
(PDF)

S7 Table Validation of some stable motif control intervention targets in Table 1 when fixing the intervened node variables close to the intervention-prescribed values in the T-LGL leukemia differential equation network model. If the intervention is 0 (1), the node variable is fixed at 0.1 (0.9), 0.8 (0.2), 0.7 (0.3), or 0.6 (0.4). The percentages are significant in the digits shown and have an estimated absolute error (standard deviation of the mean) of 6⋅10^−3^[%*p*_*Attr*_(100%−%*p*_*Attr*_)]^1/2^ %, where %*p*_*Attr*_ is the percentage shown (e.g. 0.06% for a %*p*_*Attr*_ of 1%, and 0.3% for a %*p*_*Attr*_ of 50%).
(PDF)

## References

[1] Takahashi K and Yamanaka S (2006) Induction of Pluripotent Stem Cells from Mouse Embryonic and Adult Fibroblast Cultures by Defined Factors. Cell 126 (4), 652–655. 10.1016/j.cell.2006.07.024 16904174

[2] Pera MF and Tam PPL (2010) Extrinsic regulation of pluripotent stem cells. Nature 465 (7299), 713–720. 10.1038/nature09228 20535200

[3] Young RA (2011) Control of the Embryonic Stem Cell State. Cell 144 (6), 940–954. 10.1016/j.cell.2011.01.032 21414485 PMC3099475

[4] Auffray C, Chen Z, and Hood L (2009) Systems medicine: the future of medical genomics and healthcare. Genome Med 1:2 10.1186/gm2 19348689 PMC2651587

[5] Barabási AL, Gulbahce N, and Loscalzo J (2011) Network medicine: a network-based approach to human disease. Nature Reviews Genetics 12, 56–68. 10.1038/nrg2918 21164525 PMC3140052

[6] Wolkenhauer O, Auffray C, Jaster R, Steinhoff G, and Dammann O (2013) The road from systems biology to systems medicine. Pediatric Research 73 (4-2), 502–507. 10.1038/pr.2013.4 23314297

[7] Liu Y, Slotine J, and Barabási AL (2011) Controllability of complex networks. Nature 473, 167–173. 10.1038/nature10011 21562557

[8] Müller FJ and Schuppert A (2011) Few inputs can reprogram biological networks. Nature 478, E4 10.1038/nature10543 22012402

[9] Liu Y, Slotine J, and Barabási AL (2013) Observability of complex systems. Proc Natl Acad Sci USA 110 (7), 2460–2465. 10.1073/pnas.1215508110 23359701 PMC3574950

[10] Cowan NJ, Chastain EJ, Vilhena DA, Freudenberg JS, and Bergstrom CT (2012) Nodal Dynamics, Not Degree Distributions, Determine the Structural Controllability of Complex Networks. PLoS ONE 7(6), e38398 10.1371/journal.pone.0038398 22761682 PMC3382243

[11] Cornelius SP, Kath WL, and Motter AE (2013) Realistic control of network dynamics. Nature Communications 4, 1942 10.1038/ncomms2939 23803966 PMC3955710

[12] Fiedler B, Mochizuki A, Kurosawa G, Saito D (2013) Dynamics and control at feedback vertex sets I: Informative and determining nodes in regulatory networks. J. Dyn. Differential Equations 2. 10.1016/j.jtbi.2013.06.009 23774067

[13] Mochizuki A, Fiedler B, Kurosawa G, Saito D (2013) Dynamics and control at feedback vertex sets. II: A faithful monitor to determine the diversity of molecular activities in regulatory networks. J. Theor. Biol. 335, 130–146. 10.1016/j.jtbi.2013.06.009 23774067

[14] Kalman RE (1963) Mathematical description of linear dynamical systems. J. Soc. Indust. Appl. Math. Ser. A 1, 152–192. 10.1137/0301010

[15] Luenberger DG (1979) Introduction to Dynamic Systems: Theory, Models, and Applications. Wiley.

[16] Slotine JJ, Li W (1991) Applied Nonlinear Control. Prentice-Hall.

[17] Lin CT (1974) Structural controllability. IEEE Trans. Automat. Contr. 19, 201–208. 10.1109/TAC.1974.1100557

[18] Tyson JJ, Chen KC, and Novak B (2001) Network dynamics and cell physiology. Nature Rev. Mol. Cell Biol. 2 (12), 908–916. 10.1038/35103078 11733770

[19] Tyson JJ, Chen KC, and Novak B (2003) Sniffers, buzzers, toggles and blinkers: dynamics of regulatory and signaling pathways in the cell. Curr. Op. Cell Biol. 15, 221–231. 10.1016/S0955-0674(03)00017-6 12648679

[20] Akutsu T, Hayashida M, Ching WK, and Ng MK (2007) Control of Boolean networks: Hardness results and algorithms for tree structured networks. J. Theor. Biol. 244 (4), 670–679. 10.1016/j.jtbi.2006.09.023 17069859

[21] Cheng D and Qi H (2009) Controllability and observability of Boolean control networks. Automatica 45 (7), 1659–1667. 10.1016/j.automatica.2009.03.006

[22] Akutsu T, Yang Z, Hayashida M, and Tamura T (2012) Integer Programming-Based Approach to Attractor Detection and Control of Boolean Networks. IEICE TRANS. INF. & SYST. E95-D (12), 2960–2970. 10.1587/transinf.E95.D.2960

[23] Bornholdt S (2005) Systems biology: Less is more in modeling large genetic networks. Science 310 (5747), 449–451. 10.1126/science.1119959 16239464

[24] Miskov-Zivanov N, Turner MS, Kane LP, Morel PA and Faeder JR (2013) The Duration of T Cell Stimulation Is a Critical Determinant of Cell Fate and Plasticity. Sci. Signal. 6, ra97 10.1126/scisignal.2004217 24194584 PMC4074924

[25] Benitez M, Espinosa-Soto C, Padilla-Longoria P and Alvarez-Buylla ER (2008) Interlinked nonlinear subnetworks underlie the formation of robust cellular patterns in Arabidopsis epidermis: a dynamic spatial model. BMC Systems Biology 2:98 10.1186/1752-0509-2-98 19014692 PMC2600786

[26] Saez-Rodriguez J, Alexopoulos LG, Zhang M, Morris MK, Lauffenburger DA, and Sorger PK (2011). Comparing signaling networks between normal and transformed hepatocytes using discrete logical models. Cancer Res. 71, 5400–11. 10.1158/0008-5472.CAN-10-4453 21742771 PMC3207250

[27] Orlando DA, Lin CY, Bernard A, Wang JY, Socolar JES, Iversen ES, et al (2008). Global control of cell-cycle transcription by coupled CDK and network oscillators. Nature 453, 944–947. 10.1038/nature06955 18463633 PMC2736871

[28] Zhang R, Shah MV, Yang J, Nyland SB, Liu X, Yun JK, et al (2008) Network Model of Survival Signaling in LGL Leukemia. Proc Natl Acad Sci USA 105, 16308–16313. 10.1073/pnas.0806447105 18852469 PMC2571012

[29] Morris MK, Saez-Rodriguez J, Sorger PK, and Lauffenburger DA (2010). Logic-based models for the analysis of cell signaling networks. Biochemistry 49, 3216–24. 10.1021/bi902202q 20225868 PMC2853906

[30] Wang RS, Saadatpour A, and Albert R (2012). Boolean modeling in systems biology: an overview of methodology and applications. Physical Biology 9, 055001 10.1088/1478-3975/9/5/055001 23011283

[31] Kauffman SA (1969) Metabolic stability and epigenesis in randomly constructed genetic nets. J. Theor. Biol. 22, 437–467. 10.1016/0022-5193(69)90015-0 5803332

[32] Glass L and Kauffman SA (1973) The logical analysis of continous, nonlinear biochemical control networks. J. Theor. Biol. 39, 103–129. 10.1016/0022-5193(73)90208-7 4741704

[33] Glass L (1975) Classification of biological networks by their qualitative dynamics. J. Theor. Biol. 54 (1), 85–107. 10.1016/S0022-5193(75)80056-7 1202295

[34] Thomas R, Thieffry D, and Kaufman M (1995) Dynamical behaviour of biological regulatory networks-I. Biological role of feedback loops and practical use of the concept of the loop-characteristic state. Bull. Math. Biol. 57 (2), 247–276. 10.1007/BF02460618 7703920

[35] Chaves M, Sontag ED, and Albert R (2006) Methods of robustness analysis for Boolean models of gene control networks. Syst. Biol. (Stevenage) 153 (4), 154–167 10.1049/ip-syb:20050079 16986617

[36] Saadatpour A, Albert I, and Albert R (2010) Attractor analysis of asynchronous Boolean models of signal transduction networks. J. Theor. Biol. 266, 641–656. 10.1016/j.jtbi.2010.07.022 20659480

[37] Sevim V, Gong X, and Socolar JE (2010) Reliability of Transcriptional Cycles and the Yeast Cell-Cycle Oscillator. PLoS Comput. Biol. 6 (7), e1000842 10.1371/journal.pcbi.1000842 20628620 PMC2900291

[38] Murrugarra D, Veliz-Cuba A, Aguilar B, Arat S, and Laubenbacher R (2012) Modeling stochasticity and variability in gene regulatory networks. EURASIP Journal on Bioinformatics and Systems Biology 2012: 5 10.1186/1687-4153-2012-5 22673395 PMC3419641

[39] Huang S and Ingber DE (2000). Shape-dependent control of cell growth, differentiation, and apoptosis: switching between attractors in cell regulatory networks. Exp Cell Res. 261(1), 91–103. 10.1006/excr.2000.5044 11082279

[40] Huang S, Ernberg I, Kauffman S (2009). Cancer attractors: a systems view of tumors from a gene network dynamics and developmental perspective. Semin Cell Dev Biol. 7, 869–76. 10.1016/j.semcdb.2009.07.003 PMC2754594 19595782

[41] Zañudo JGT and Albert R (2013) An effective network reduction approach to find the dynamical repertoire of discrete dynamic networks. Chaos 23 (2), 025111 Focus Issue: Quantitative Approaches to Genetic Networks 10.1063/1.4809777 23822509

[42] Bilke S and Sjunnesson F (2001) Stability of the Kauffman model. Phys. Rev. E 65, 016129. 10.1103/PhysRevE.65.016129 11800758

[43] Naldi A, Remy É Thieffry D, and Chaouiya C (2011) Dynamically consistent reduction of logical regulatory graphs. Theor Comput Sci 412, 2207–2218. 10.1016/j.tcs.2010.10.021

[44] Veliz-Cuba A (2011) Reduction of Boolean network models. J. Theor. Biol. 289, 167–172. 10.1016/j.jtbi.2011.08.042 21907211

[45] Steinway SN, Zañudo JGT, Ding W, Rountree CB, Feith DJ, Loughran TP, et al (2014) Network Modeling of TGFβ Signaling in Hepatocellular Carcinoma Epithelial-to-Mesenchymal Transition RevealsJoint Sonic Hedgehog and Wnt Pathway Activation. Cancer Research 74 (21), 5963–77. 10.1158/0008-5472.CAN-14-0225 25189528 PMC4851164

[46] Saadatpour A, Wang RS, Liao A, Liu X, Loughran TP, Albert I, et al (2011) Dynamical and Structural Analysis of a T Cell Survival Network Identifies Novel Candidate Therapeutic Targets for Large Granular Lymphocyte Leukemia. PLoS Computational Biology 7(11): e1002267 10.1371/journal.pcbi.1002267 22102804 PMC3213185

[47] Shah MV, Zhang R, Irby R, Kothapalli R, Liu X, Arrington T, et al (2008) Molecular profiling of LGL leukemia reveals role of sphingolipid signaling in survival of cytotoxic lymphocytes. Blood 112, 770–781. 10.1182/blood-2007-11-121871 18477771 PMC2481553

[48] Naldi A, Carneiro J, Chaouiya C, and Thieffry D (2010) Diversity and Plasticity of Th Cell Types Predicted from Regulatory Network Modelling. PLoS Computational Biology 6(9): e1000912 10.1371/journal.pcbi.1000912 20824124 PMC2932677

[49] Wittmann DM, Krumsiek J, Saez-Rodriguez J, Lauffenburger DA, Klamt S, and Theis FJ (2009) Transforming Boolean models to continuous models: Methodology and application to T-cell receptor signaling. BMC Systems Biology 3, 98 10.1186/1752-0509-3-98 19785753 PMC2764636

[50] Kharas MG, Janes MR, Scarfone VM, Lilly MB, Knight ZA, Shokat KM, et al (2008) Ablation of PI3K blocks BCR-ABL leukemogenesis in mice, and a dual PI3K/mTOR inhibitor prevents expansion of human BCR-ABL+ leukemia cells. J Clin Invest. 118(9), 3038–50. 10.1172/JCI33337 18704194 PMC2515380

[51] Bozic I, Reiter JG, Allen B, Antal T, Chatterjee K, Shah P, et al (2013) Evolutionary dynamics of cancer in response to targeted combination therapy. Elife 2:e00747 10.7554/eLife.00747 23805382 PMC3691570

[52] Al-Lazikani B, Banerji U, and Workman P (2012) Combinatorial drug therapy for cancer in the post-genomic era. Nature biotechnology 30, 679–92. 10.1038/nbt.2284 22781697

[53] Campbell C, Yang S, Albert R, and Shea K (2011) A network model for plant-pollinator community assembly. Proc Natl Acad Sci USA 108 (1), 197–202. 10.1073/pnas.1008204108 21173234 PMC3017189

[54] Castellano C, Fortunato S, and Loreto V (2009) Statistical physics of social dynamics. Rev. Mod. Phys. 81, 591 10.1103/RevModPhys.81.591

[55] Fernández-Gracia J, Suchecki K, Ramasco JJ, San Miguel M, and Eguíluz VM (2014) Is the Voter Model a Model for Voters? Phys. Rev. Lett. 112, 158701 10.1103/PhysRevLett.112.158701 24785078

[56] Pastor-Satorras R and Vespignani A (2001) Epidemic Spreading in Scale-Free Networks Phys. Rev. Lett. 86, 3200 10.1103/PhysRevLett.86.3200 11290142

[57] Víctor M. Eguíluz and Klemm Konstantin (2002) Epidemic Threshold in Structured Scale-Free Networks Phys. Rev. Lett. 89, 108701 10.1103/PhysRevLett.89.108701 12225235

[58] Krumsiek J, Pölsterl S, Wittmann DM, and Theis FJ (2010) Odefy-from discrete to continuous models. BMC Bioinformatics 11, 233 10.1186/1471-2105-11-233 20459647 PMC2873544

[59] Yang J, Liu X, Nyland SB, Zhang R, Ryland LK, Broeg K, et al (2010) Platelet-derived growth factor mediates survival of leukemic large granular lymphocytes via an autocrine regulatory pathway. Blood 115, 51–60. 10.1182/blood-2009-06-223719 19880494 PMC2803691

[60] Lamy T, Liu JH, Landowski TH, Dalton WS, and Loughran TP Jr (1998) Dysregulation of CD95/CD95 ligand-apoptotic pathway in CD3+ large granular lymphocyte leukemia. Blood 92, 4771–4777. 9845544

[61] Epling-Burnette PK, Bai F, Wei S, Chaurasia P, Painter JS, Olashaw N, et al (2004) ERK couples chronic survival of NK cells to constitutively activated Ras in lymphoproliferative disease of granular lymphocytes (LDGL). Oncogene 23, 9220–9229. 15516985 10.1038/sj.onc.1208122

[62] Schade AE, Powers JJ, Wlodarski MW, and Maciejewski JP (2006) Phosphatidylinositol-3-phosphate kinase pathway activation protects leukemic large granular lymphocytes from undergoing homeostatic apoptosis. Blood 107, 4834–4840. 10.1182/blood-2005-08-3076 16484592 PMC1895814

[63] Glimcher LH and Murphy KM (2000) Lineage commitment in the immune system: the T helper lymphocyte grows up. Genes Dev 14, 1693–711. 10898785

[64] Szabo SJ, Kim ST, Costa GL, Zhang X, Fathman CG, and Glimcher LH (2000) A novel transcription factor, T-bet, directs Th1 lineage commitment. Cell 100 (6), 655–69. 10.1016/S0092-8674(00)80702-3 10761931

[65] Zheng W and Flavell RA (1997) The transcription factor GATA-3 is necessary and sufficient for Th2 cytokine gene expression in CD4 T cells. Cell 89 (4), 587–96. 10.1016/S0092-8674(00)80240-8 9160750

[66] Zhou L, Ivanov II, Spolski R, Min R, Shenderov K, Egawa T, Levy DE, Leonard WJ, and Littman DR (2007) IL-6 programs T(H)-17 cell differentiation by promoting sequential engagement of the IL-21 and IL-23 pathways. Nat Immunol. 8 (9), 967–74. 10.1038/ni1488 17581537

[67] Hori S, Nomura T, Sakaguchi S (2003) Control of regulatory T cell development by the transcription factor Foxp3. Science 299, 1057–61. 10.1126/science.1079490 12522256

