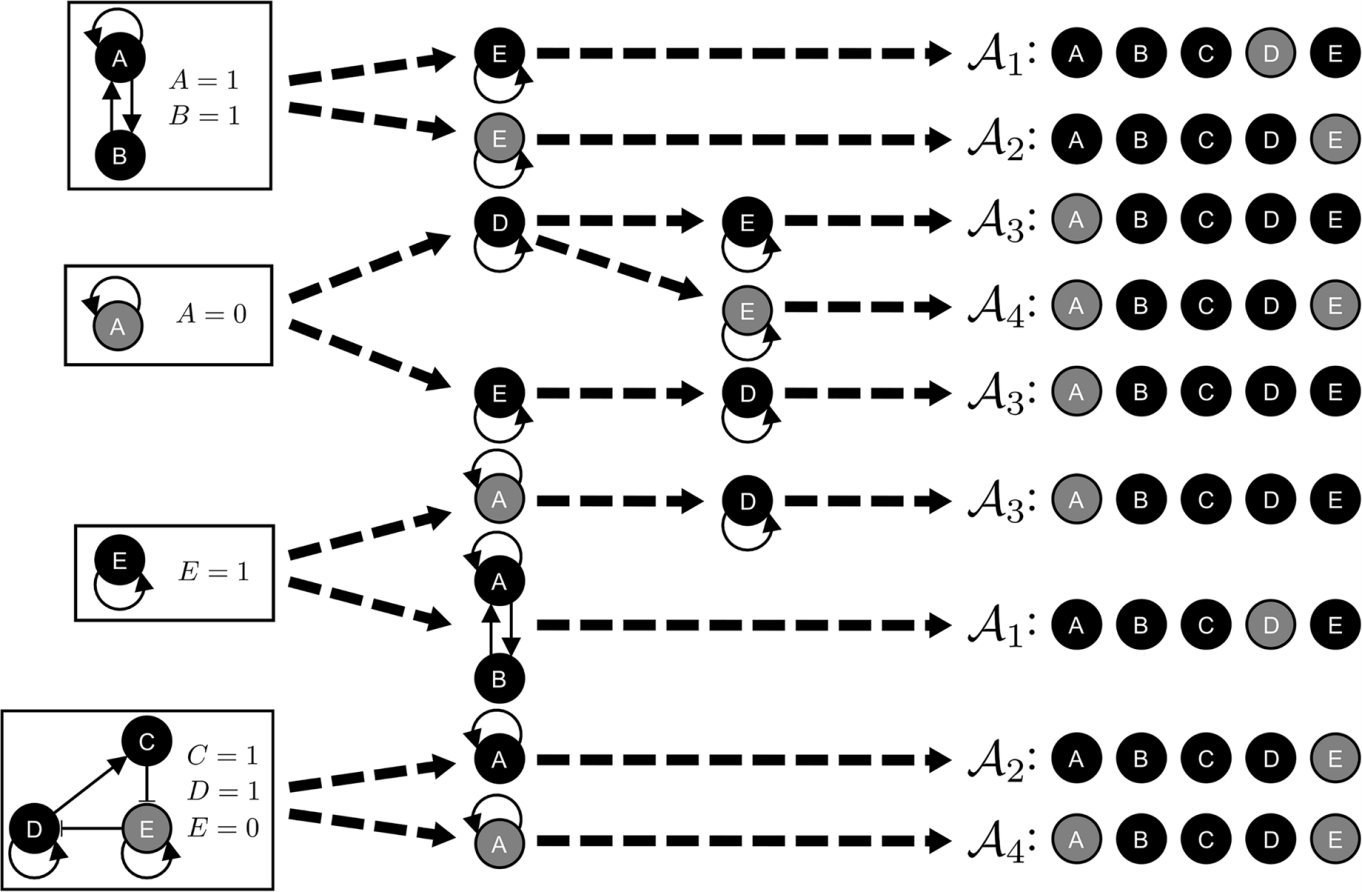# Correction: Cell Fate Reprogramming by Control of Intracellular Network Dynamics

**DOI:** 10.1371/journal.pcbi.1004672

**Published:** 2015-12-23

**Authors:** The PLOS Computational Biology Staff

An error was introduced during the production process. The shading in Fig 2 is incorrect. The publisher apologizes for the error. Please view the correct version here:

**Fig 2:**