# Supplementary material for: Cell Fate Reprogramming by Control of Intracellular Network Dynamics
Source: PLoS Comput Biol. 2015 Apr 7;11(4):e1004193. doi: 10.1371/journal.pcbi.1004193 (PMC4388852; doi:10.1371/journal.pcbi.1004193)
Supplement: S1 Text — (PDF) [file pcbi.1004193.s001.pdf]

Supporting Information

S1 TEXT. DETAILS AND EXAMPLES OF THE ATTRACTOR FINDING METHOD AND STABLE MOTIF CONTROL ALGORITHM

A. Attractor-finding method

The task of finding attractors for a Boolean network is limited by the exponential growth of the state space with the number of number of nodes N. As a consequence, a full search of the state space to find the attractors is viable only for small networks ($N \lesssim 20$). To overcome this problem when dealing with intracellular networks (or, more generally, with sparse networks), we recently proposed an alternative approach to find the attractors of a Boolean network model [41]. This approach successfully found the attractors of a previously developed biological network model composed of 60 nodes [28, 41], and of an ensemble of random Boolean networks composed of up to hundreds of nodes [41]. It has also been proven to find both fixed point and complex attractors. More formally, the result of the attractor-finding method are the so-called quasi-attractors, each of which has a corresponding system attractor (see S2 Text section A or ref. [41] for details). A quasi-attractor is a set of network states in which each node state is either fixed (0 or 1) or is not specified, in which case it is expected to oscillate. The difference between an attractor and a quasi-attractor is that an attractor includes the nodes that oscillate and the precise network states they can take, while the quasi-attractor does not specify the precise network states that the oscillating nodes take. For a more detailed explanation and the step-by-step algorithm of the attractor-finding method see S2 Text section A or ref. [41].

B. Stable motif identification

Stable motifs are function-dependent network components (subnetworks) in a Boolean model that must stabilize in a fixed state. These network components and their respective fixed states are identified with a certain type of strongly connected component (or SCC, a subgraph in a directed network for which all node pairs are connected by paths in both directions) in an expanded representation of the Boolean network [41, S1]. The expanded network representation explicitly incorporates the combinatorial nature and the sign of the interactions. This is achieved by introducing complementary nodes for every node, which are used to indicate negative regulation in a Boolean function (NOT relationship), as well as introducing a composite node to denote a conditional dependence (AND relationship) among two or more inputs in a Boolean function. A detailed explanation of the expanded network representation can be found in S2 Text section A.1 and ref. [41].

As an example, consider node C in the example network in Fig. 1. The expanded network representation of C and its complementary node $\overline{C}$ is shown in S2(a) Figure. The function $f_C = (A \text{ AND } B) \text{ OR } D$ contains an AND relationship between the state of node A and the state of node B, so a composite node AB is added when expanding the network. Node A and B are connected by directed edges to the composite node AB, and an edge from AB to C is also present. Since the state of node D is OR-separated from the (A AND B) term, an edge from D to A is part of the expanded network. A complementary node $\overline{C}$ is also added in the expanded network, with an associated Boolean function $f_{\overline{C}} = \overline{f}_C = (\text{NOT } A \text{ AND NOT } D) \text{ OR } (\text{NOT } B \text{ AND NOT } D)$. The expanded network will contain the composite nodes $\overline{AD}$ and $\overline{BD}$, directed edges from $\overline{A}$ and $\overline{D}$ to $\overline{AD}$, directed edges from $\overline{B}$ and $\overline{D}$ to $\overline{BD}$, and directed edges from $\overline{AD}$ and $\overline{BD}$ to $\overline{C}$. As another example, the expanded network representation of B and $\overline{B}$ is shown in S2(b) Figure.

In the expanded network representation, stable motifs correspond to minimal strongly connected components that satisfy two properties: (1) the strongly connected component does not contain both a node and its complementary node, and (2) if the strongly connected component contains a composite node, all of its input nodes must also be part of the strongly connected component. A more detailed explanation of the method for identifying stable motifs is given in S2 Text and ref. [41]. The main point is that a stable motif can be identified with a set of nodes that form a minimal strongly connected component, and that a stable motif's corresponding states are such that they form a partial fixed point of the Boolean model (for a Boolean model with node variables $\{\sigma_i\}$ and associated functions $\{f_i\}$, a partial fixed point is a set of node states $P = \{\sigma_{p_1} = s_{p_1}, \sigma_{p_2} = s_{p_2}, \ldots, \sigma_{p_l} = s_{p_l}\}$ such that if Σ_P is any network state in which $\sigma_{p_k} = s_{p_k} \forall p_k \in \{p_1, p_2, \ldots, p_l\}$, then $f_{p_j}(\Sigma_P) = s_{p_j}$.).

As an example, consider the logical network in Fig. 1(a) in the main text, and its associated stable motifs in Fig. 1(b). The expanded network representation of these stable motifs is shown in the leftmost column of S5 Figure and their corresponding node states are shown in the middle column of S5 Figure.

C. Network reduction

Network reduction techniques [36, 42–44] are used to simplify a network when a node's state is known to be fixed, for example, in the case of a sustained signal. The downstream effect of this fixed state is evaluated by setting the fixed node state of interest in the Boolean function of its target nodes. As a consequence, a target node's modified Boolean function may only have one possible outcome, which means the target node's state is fixed. The whole procedure is repeated iteratively until no new fixed node states are obtained. These fixed-state nodes and their edges can be eliminated from the network.

In our work, this reduction method is used to evaluate the effect of each separate stable motif on the rest of the network [41]. This is done by applying network reduction separately for each stable motif of the network, using the stable motif's corresponding states as the initial fixed node states. The result is a set of simplified Boolean networks, each of which corresponds to a separate stable motif, and a set of node states for each simplified network, with the latter being the node states that stay fixed due to their respective stable motifs.

D. Dependence of stable motifs and attractors on the logical functions

An important question related to the attractor-finding method (and, thus, to the stable motif control algorithm) is how stable motifs and attractors depend on the logical functions of the logical network in consideration. The attractor-finding method takes as an input a given logical network, which includes both the topology of the network and the associated logic functions. Given that any topological or functional change in the logical network gives rise to a different logical network (potentially similar or potentially very different, depending on the extent of the change), the attractor-finding method needs to be applied again to the modified logical network to fully assess if the change impacts the stable motifs and/or the attractor landscape.

Even though the task of assessing the change that an arbitrary change in a logical functions brings about on a stable motif and/or the attractors is a complicated problem, it is possible to identify sufficient conditions for a target stable motif and/or attractor to be conserved after a change in the functions or topology of the logical network. For the case of a stable motif, this is done by identifying the terms of the logical functions associated with the stable motif; these terms are part of the formal definition of stable motifs in the expanded network representation of the network.

As an example, consider the logical network in Fig. 1(a) in the main text, and its associated stable motifs in Fig. 1(b). These stable motifs are shown in their expanded network representation in S5 Figure, together with the associated terms of the logical function. The sufficient condition for the preservation of a stable motif is that the terms associated with it stay the same. For the case of an attractor, one needs to identify the terms related to the stable motifs in each sequence associated with the attractor, and also consider the terms in the logical functions responsible for the node states that get fixed during the network reduction portion of the attractor-finding method. The whole process can become quite convoluted and is beyond the scope of this work.

E. Quasi-attractors, oscillations, and the stable motif control algorithm

The stable motif control algorithm uses as a starting point the stable motif succession diagram obtained from the attractor-finding method in ref. [41]. As discussed in section A, the output of the attractor-finding method is, formally, not the system's attractors but its quasi-attractors, each of which is a network state which captures a steady state exactly and is a compressed representation of a complex (oscillating) attractor. A consequence of the relation between quasi-attractors and attractors is that certain networks with special types of complex attractors need to be treated with care when our method is applied. These special types of attractors were called unstable oscillations and incomplete oscillations in ref. [41].

Unstable and incomplete oscillations denote the dynamical behavior of the node state of a group of nodes that form a special type of SCC in the expanded network representation described in section A. In unstable oscillations the node state of the nodes forming the SCC oscillate in an attractor, yet are fixed in another attractor that differs only in the state of these nodes (and, potentially, on the state of nodes affected by the state of nodes in the SCC). In incomplete oscillations the node state of the nodes forming the SCC oscillate in an attractor, but do not visit all possible states of their sub-state-space in the attractor. Incomplete oscillations are the reason why undetermined states in a quasi-attractor do not necessarily oscillate.

These special types of attractors pose a challenge to the attractor-finding method, in the sense that one needs to go beyond identifying stable motifs to also identify potential unstable oscillations and incomplete oscillations. Our method can identify when a given network has the potential to have this special type of complex attractor by an extra

step of analysis involving what we called oscillating components, and may in some cases involve an exploration of the sub-state-space associated with the potentially-oscillating network components. For more details, see S2 Text section A or ref. [41].

In some cases, the sub-state-space associated with the potentially-oscillating network components is too large to fully enumerate. In these cases, the stable motif succession diagram we can obtain without exploring this sub-state-space has an outgoing arrow which may not exist in the full stable motif succession diagram, after which there may be an attractor not found in the rest of the motif succession diagram. As a consequence, we only have partial knowledge of the full stable motif succession diagram. As we discuss in S3 Text section B.3, partial knowledge of the stable motif succession diagram does not compromise the effectiveness of the stable motif control algorithm for the attractors in the part of the motif succession diagram we have knowledge of, but it does require us to use a modified stable motif control algorithm in which step 2 of the original algorithm is skipped.

As an example of unstable oscillations, consider the Boolean network shown in S3 Figure, which is the simplest example (up to a relabeling of node states) of unstable oscillations. The network and logical functions are given in S3(a) Figure, the state space of the system under asynchronous updating is given in S3(b) Figure, and the stable motif succession diagram is given in S3(c) Figure. Note that the states of nodes A and B oscillate between three network states in an attractor $(\{(A = 1, B = 0), (A = 0, B = 0), (A = 0, B = 1)\})$, while they are fixed in another attractor $(\{A = 1, B = 1\})$. Applying the attractor-finding method to this network, we find a stable motif $\{A = 1, B = 1\}$ and find that the set of nodes $\{A, B\}$ satisfy the necessary conditions to display unstable oscillations. Since A and B satisfy the necessary conditions to display unstable oscillations, one needs to search the state space spanned by this set of nodes, which in this case corresponds to the whole state space. Doing so, one finds that there is an unstable oscillation between the network states $\{A = 0, B = 1\}$, $\{A = 0, B = 0\}$, and $\{A = 1, B = 0\}$. The motif succession diagram in this case has the stable motif $\{A = 1, B = 1\}$ and the oscillating motif $(\{A = 0, B = 1\}$, $\{A = 0, B = 0\}$, $\{A = 1, B = 0\})$, as shown in in S3(c) Figure.

As an example of incomplete oscillations, consider the Boolean network shown in S4 Figure. The network and logical functions are given in S4(a) Figure, the state space of the system under asynchronous updating is given in S4(b) Figure, and the stable motif succession diagram is given in S4(c) Figure. Note that the states of nodes A and B oscillate between three subnetwork states in the attractors $(\{(A = 1, B = 0), (A = 0, B = 0), (A = 0, B = 1)\})$, and thus, A and B do not visit all possible states of their sub-state-space in each attractor. The result of applying the attractor-finding method to this network is a stable motif $\{C = 0\}$ and that the set of nodes $\{A, B\}$ satisfies the conditions to display incomplete oscillations. Since A and B satisfy the necessary conditions to display incomplete oscillations, one needs to search the state space spanned by A and B. Doing so, one finds that there is an incomplete oscillation between the states $(\{A = 0, B = 1\}$, $\{A = 0, B = 0\}$, $\{A = 1, B = 0\})$. The stable motif succession diagram for this Boolean network has the stable motif $\{C = 0\}$ and the oscillating motif $(\{A = 0, B = 1\}$, $\{A = 0, B = 0\}$, $\{A = 1, B = 0\})$.

F. Rationale and example of step 2 of the stable motif control algorithm

The aim of step 2 of the stable motif control algorithm (see Methods) is to simplify the sequences of stable motifs so that the number of nodes that need to be controlled is minimized. This is done by identifying motifs after which all consequent motifs lead to the same attractor and then removing these consequent motifs from the sequence. To illustrate this, consider the stable motif succession diagram shown in Fig. S1.1. Since every possible motif after motif 1 leads to attractor 1, fixing the node states associated to motif 1 is enough to prod the system towards attractor 1. Step 2 makes sure that motifs 2 - 4 are removed from the sequences of stable motifs associated to attractor 1, since they are not necessary for the system to reach attractor 1.

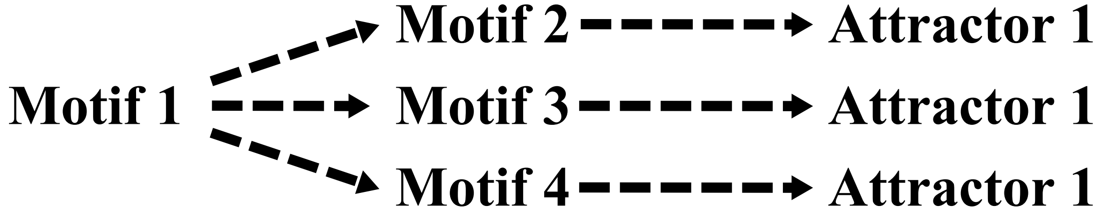

Fig. S1.1. Example stable motifs succession diagram illustrating the simplification brought about by step 2 of the stable motif control algorithm. Step 2 removes motifs 2 - 4 from the sequences of stable motifs associated to attractor 1.

G. Step by step description of the stable motif control algorithm applied to the network in Fig. 1(a)

Consider the network in Fig. 1(a) and choose $\mathcal{A}_2$ in Fig. 2 as our target attractor. Following step 1 and using the stable motif succession diagram (Fig. 2), we obtain two sequences of stable motifs that lead to $\mathcal{A}_2$: $\mathcal{S}_1$ = ({ A=1, B=1 } , { E=0 }) and $\mathcal{S}_2$ = ({ C=1, D=1, E=0 } , { A=1 }). For these sequences, step 2 provides no simplification. Following step 3, the four stable motifs involved give only one subset of motif states per motif. For the first sequence, these subsets of states are M_1 = { A=1, B=1 } for $\mathcal{M}_1$ = { A=1, B=1 } and M_2 = { E=0 } for $\mathcal{M}_2$ = { E=0 }. For the second sequence, the states are M_3 = { E=0 } for $\mathcal{M}_3$ = { C=1, D=1, E=0 } and M_4 = { A=1 } for $\mathcal{M}_4$ = { A=1 }. The result of step 3 are the sequences $\mathcal{S}_1 = (O_1, O_2)$, where O_1 = {A=1} and O_2 = {E=0}, and $\mathcal{S}_2 = (O_3, O_4)$, where O_3 = {E=0} and O_4 = {A=1}. Since each O_i contains a single state, step 4 gives one set of states for each sequence: $\mathcal{C}_1$ = { A=1, E=0 } for $\mathcal{S}_1$ and $\mathcal{C}_2$ = { E=0, A=1 } for $\mathcal{S}_2$. Since both states are the same, the network control target for attractor $\mathcal{A}_2$ contains a single set of states, $C_{\mathcal{A}_2}$ = { { A=1, E=0 } }.
