# Supplementary material for: Cell Fate Reprogramming by Control of Intracellular Network Dynamics
Source: PLoS Comput Biol. 2015 Apr 7;11(4):e1004193. doi: 10.1371/journal.pcbi.1004193 (PMC4388852; doi:10.1371/journal.pcbi.1004193)
Supplement: S2 Text — (PDF) [file pcbi.1004193.s002.pdf]

Supporting Information

S2 TEXT. MATHEMATICAL FOUNDATIONS OF THE ATTRACTOR-FINDING METHOD AND OF THE STABLE MOTIF CONTROL APPROACH

In this part we describe the methods used in our work in a formal way. Part of the text in section A is adapted from our previous work (ref. [41]). For the propositions, lemmas, and theorems in section A, which we proved in our previous work [41], we restrict ourselves to reproducing their statements and explaining their meaning, and refer the reader to our previous work [41] for the proof.

In the following we use $V = (v_1, v_2, \ldots, v_N)$ to represent the N nodes of the Boolean network, $\sigma_i, i = 1, 2, \ldots, N$ to represent the state of node v_i, $\Sigma = (\sigma_1, \sigma_2, \ldots, \sigma_N)$ to represent the states of all nodes (also called a network state), $f_i, i = 1, 2, \ldots, N$ to represent the Boolean function of node v_i, and $F = (f_1, f_2, \ldots, f_N)$ to represent all the Boolean functions. We use $f(\Sigma)$ to denote a Boolean function f evaluated at a network state Σ, and $f|_P$ to denote a Boolean function where only the state of a subset of nodes $P = \{\sigma_{p_1}, \sigma_{p_2}, \ldots, \sigma_{p_l}\}$ is evaluated. We commonly use b_i to indicate that a specific value for node state σ_i is chosen, that is, $\sigma_i = b_i$.

We assume, for convenience, that the Boolean functions $f_i, i = 1, 2, \ldots, N$ satisfy these properties:

1. The f_i's do not take constant values (i.e. $f_i \neq 0$ and $f_i \neq 1$).
2. If f_i depends on the state of node v_j, σ_j, then there must be at least one pair of network states $\Sigma^{(1)}$ and $\Sigma^{(2)}$ with $\sigma_j^{(1)} \neq \sigma_j^{(2)}$, and $\sigma_k^{(1)} = \sigma_k^{(2)}$ for all $k \neq j$, such that $f_i(\Sigma^{(1)}) \neq f_i(\Sigma^{(2)})$.
3. The f_i's are written in a disjunctive normal form:
$$\begin{aligned} f_i = &(s_1 \text{ AND } s_2 \text{ AND } \cdots \text{ AND } s_k) \text{ OR } (s_{k+1} \text{ AND } s_{k+2} \text{ AND } \cdots s_l) \\ &\text{OR } \cdots \text{ OR } (s_m \text{ AND } s_{m+1} \text{ AND } \cdots \text{ AND } s_n)\,, \end{aligned}$$
where the s_j's are either the states of one of the input nodes of f_i, or one of these states' negations.
4. If for M, denoting a state of a subset of the inputs of f_i, one has $f_i|_M = 1$ (regardless of the states of the remaining inputs), then the disjunctive form of f_i must have at least one of its conjunctive clauses equal to 1 when evaluated at the state M of this subset of nodes.

The first property makes sure we have no source nodes. For our purposes this can be assumed without loss of generality, because even if that is not the case, we can use the reduction method of Saadatpour et al. [36, 46] and remove all source nodes while preserving all attractors [S2]. The second property can also be assumed without any loss of generality; it is just a way of stating that we consider f_i to depend on σ_j only if it explicitly depends on σ_j for at least a pair of network states. The third and fourth property are also general, since one can construct the respective disjunctive normal form from the truth table of the Boolean function.

The dynamics of a Boolean network (V, Σ, F) is determined using a stochastic updating scheme known as the general asynchronous scheme [33, 34, 36]. In the general asynchronous scheme, the state of the nodes is updated at discrete time steps starting from an initial condition. At every time step, one of the variables (σ_j) is chosen randomly (uniformly) and is updated using its respective function and the state of its regulators at the previous time step

$$\sigma_j(t+1) = f_j\left(\sigma_{j_1}(t), \sigma_{j_2}(t), \ldots, \sigma_{j_{k_j}}(t)\right),$$

while the rest of the variables retain their state.

A. Expanded network/network reduction attractor-finding method of ref. [41]

1. *The expanded network representation*

In order to identify the stable motifs of a Boolean network, we use a representation that incorporates explicitly the update functions f_i. Previous work [41, S1] has shown that a useful representation for this purpose is the so-called expanded network representation.

The creation of the expanded network consists of two basic operations. First, we introduce a complementary node $\overline{v}_i$ for every node v_i in the network and assign to each $\overline{v}_i$ an update function $\overline{f}_i$ which is the Boolean negation of v_i's

update function f_i. The update functions $\overline{f}_i$ are assumed to satisfy the same four properties as the update functions f_i without loss of generality. Second, to incorporate the combinatorial nature of the update functions, we introduce a composite node $v^{(comp)}$ for each set of synergistic interactions (that is, for each conjunctive clause) in the Boolean functions f_i, with a Boolean function $f^{(comp)}$ given by its respective conjunctive clause. In the following we commonly refer to nodes that are not complementary nor composite as normal nodes.

The expanded network $G_{exp} = (V_{exp}, E_{exp}, F_{exp})$ consists of a directed graph (V_{exp}, E_{exp}) and a set of Boolean functions F_{exp}, where V_{exp} is the union of all normal nodes V, all complementary nodes $\overline{V}$, and all composite nodes $V^{(comp)}$, E_{exp} is to be defined soon, and F_{exp} is the union of the Boolean functions of all normal nodes F, the Boolean functions of all complementary nodes $\overline{F}$, and the Boolean functions of all composite nodes $F^{(comp)}$. The connectivity E_{exp} of the expanded network is defined by the type of node considered. If $v \in V_{exp}$ is a composite node, then its associated Boolean function has the form $f = s_1$ AND s_2 AND $\cdots$, and v has an input for each s_j; if s_j is a node state (the negation of a node state), the input is a normal node (complementary node). If $v \in V_{exp}$ is not a composite node, then its Boolean function has the form $f = S_1$ OR S_2 OR $\cdots$, and v has an input for each S_j; the input is a composite node if S_j is a conjunctive clause, and a normal node (complementary node) if it is a node state (the negation of a node state).

2. *Identifying stable motifs from the expanded network*

We define a stable motif M in the expanded network as any of the smallest strongly connected components (SCCs) in the expanded network representation which satisfy these two properties:

1. If M contains a normal node v_i (complementary node $\overline{v}_i$) then M does not contain its corresponding complementary node $\overline{v}_i$ (normal node v_i).

2. If M contains a composite node $v^{(comp)}$, then all input nodes of $v^{(comp)}$ are elements of M.

The first condition makes sure that there is no contradiction between the SCCs found and a state in the original Boolean network, wherein every node can either take the value 0 or 1. The second condition is a consequence of the synergistic nature of composite nodes, which means that a composite node and all of its inputs form an irreducible unit. (By smallest SCC, we mean any SCC that does not contain another SCC with the specified properties, but that, otherwise, is arbitrary in size.)

The composition of the stable motif M directly determines the states M_{state} of a set of nodes M_{nodes} in the original Boolean network: for every normal node v_i of the stable motif the corresponding node v_i of the Boolean network adopts the state 1 ($\sigma_i = 1$), and for every complementary nodes $\overline{v}_i$ in the stable motif the corresponding node v_i of the Boolean network adopts the state 0 ($\sigma_i = 0$). This set of nodes M_{nodes} of the Boolean network and their corresponding states M_{state} is what we defined as a stable motif in the main text, and they are such that the nodes form a minimal strongly connected component and their states form a partial fixed point of the Boolean model.

3. *Network reduction*

Once the stable motifs of the network are identified, the next step is to determine the influence of these nodes on the rest of the network. More specifically, for each stable motif found, we want to find the nodes in the network whose state is fixed by the influence of this stable component. We adapt the method previously developed by Saadatpour et al. [36, 46] to simplify the network, which has been shown to preserve both the fixed points and the complex attractors of the system [S2]. It consists of two steps:

1. Identify a set of nodes $\{v_{p_1}, v_{p_2}, \ldots, v_{p_l}\}$ whose state is fixed during the dynamics, which we refer to as source nodes; for the attractor-finding method these initially correspond to the nodes in the stable motif being considered.

2. Modify the Boolean functions of the nodes downstream of the source nodes by setting the state of the source nodes to their fixed values $P = \{\sigma_{p_1} = b_{p_1}, \sigma_{p_2} = b_{p_2}, \ldots, \sigma_{p_l} = b_{p_l}\}$, that is, the modified function is given by $f|_P$. If a downstream node's modified function can only have one possible outcome, then this node can be used as a source node itself.

For each separate stable motif found in the expanded network, these two steps are repeated recursively until neither of them can be applied anymore.

4. The attractor-finding method algorithm and quasi-attractors

After network reduction, we obtain a set of states for each stable component, each of which corresponds to the states of the nodes in the stable motif and the states of other nodes which are fixed as a consequence of the stable motif. For each of these sets of states, there is also a reduced network that contains the nodes whose state we still do not know. On each of these reduced networks the whole method is applied again and iteratively until there are no more nodes with unknown states or no new stable motifs are found. This version of the attractor-finding method algorithm does not consider oscillatory behavior such as the one shown in S3 Figure; we come back to these cases in S2 Text subsections A.5 and A.6.

The attractor-finding method algorithm is summarized below.

1. Take the original Boolean network as the starting set of Boolean networks.

2. Create the expanded network representation for each of the Boolean networks.

3. Search the expanded network for stable motifs.

4. For every separate stable motif create a copy of the current network. On each of the networks created use the states of the corresponding stable motif as inputs and apply the two steps of the network reduction recursively until neither of them can be applied anymore.

5. Repeat 2-4 iteratively until there are no more nodes with unknown states or no new stable motifs are found.

For the case where there are no more nodes with unknown states, a fixed point attractor of the system is obtained directly from the state of the nodes of the fixed-state components. For the cases in which there are no new stable motifs in the final reduced networks, the state of the nodes making up said networks is still unknown. Since our method is based on identifying nodes that are fixed in a specific steady state, the expectation is that these leftover nodes oscillate in an attractor of the system, while in that same attractor the rest of the nodes take the steady state value found during the simplification process that leads to the reduced network in consideration. We refer to the final output of our method, consisting of a set of fixed-state nodes (and their states) and a (potentially empty) set of nodes with undetermined states as a *quasi-attractor*.

Quasi-attractors are closely related to the attractors of a network, both fixed points and complex attractors. For example, if the set of undetermined states in a quasi-attractor is empty, then the states of the fixed-state nodes correspond to the node states in a fixed point attractor, thus, this quasi-attractor is in fact a fixed point. More generally, for every attractor of the system there exists a quasi-attractor associated to it; this quasi-attractor is such that every node whose state is fixed in the quasi-attractor also has its state fixed in the same state in the attractor it is associated to. The proof of this is statement is given in Theorem 1 in ref. [41], which is reproduced in S2 Text subsection A.7.

5. Oscillating components and oscillations

The expanded network representation can be used to identify nodes that form an SCC in the original network, whose node states are not fixed in a complex attractor (i.e. their state oscillates). We refer to these nodes as *oscillating motifs* or *oscillating components*. To find the oscillating components O using the expanded network representation, we search for the largest SCCs that satisfy these properties:

1. If O contains a normal node v_i then O also contains its corresponding complementary node $\overline{v}_i$, and vice versa.

2. If O contains a composite node $v^{(comp)}$, then all input nodes of $v^{(comp)}$ are elements of O.

The first of these conditions makes sure that all nodes oscillate, by having both states of every node as part of the SCC. The second condition is a consequence of a composite node and all of its inputs forming an irreducible unit. In this case we look for the largest SCCs because we want to find all the nodes that feed back to each other in the oscillation.

These properties are necessary but not sufficient conditions for a group of node states to oscillate. We have found that there is a third condition that, if also satisfied, is sufficient (though not necessary) for a group of node states to oscillate, which is that (3) the oscillating component cannot contain stable motifs composed only of normal and complementary nodes. This extra condition is related to the possibility of the coexistence of a steady state and a complex attractor in the sub-state-space. The simplest example that shows this kind of behavior, which we denote

unstable oscillation, is shown in S3 Figure. In general, during the reduction process, we need to find the components that could display unstable oscillations (that is, that satisfy (1) and (2), but not (3)) to make sure that we preserve all attractors. As a consequence, we obtain a group of quasi-attractors that may not have a corresponding attractor; we refer to these quasi-attractors as marked quasi-attractors in the step-by-step algorithm in S2 Text subsection A.6.

Another type of dynamical behavior of the oscillating components that needs to be considered is when the nodes of the oscillating components do not visit all possible states of their sub-state-space in an attractor, which we refer to as an *incomplete oscillation.* As shown in Lemma 3 in ref. [41] (reproduced in subsection A.7), nodes whose state is undetermined in a quasi-attractor are downstream of the nodes whose state oscillates in the attractor corresponding to the considered quasi-attractor. Incomplete oscillations are important because a node that is downstream of an oscillating component that displays incomplete oscillations may reach a steady state as a consequence of the nodes of the component only visiting part of their sub-state-space. Incomplete oscillations are the reason why undetermined states in a quasi-attractor do not necessarily oscillate.

6. The full algorithm of the expanded network/network reduction attractor-finding method

In the following we describe the full algorithm of the attractor-finding method. Unlike the algorithm introduced in S2 Text subsection A.4, the following algorithm considers the so-called unstable oscillations, such as the one shown in S3 Figure. During the description of the algorithm we refer the reader to the subsections in S2 Text where each of these steps are described in more detail.

1. For every combination of the states of the source nodes (nodes with no upstream components) apply the two steps of network reduction method described in S2 Text subsection A.3 recursively until neither of them can be applied anymore.

2. Create the expanded network representation for each of the resulting networks (S2 Text subsection A.1).

3. Search the expanded network for stable motifs (S2 Text subsection A.2) and oscillating components (S2 Text subsection A 5).

4. For every separate stable motif create a copy of the current network. On each of the networks created use the states of the corresponding stable motif as inputs and apply the two steps of the network reduction described in S2 Text subsection A.3 recursively until neither of them can be applied anymore.

5. For every oscillating component of more than two nodes (i.e., every oscillating component that could display incomplete oscillations) create a copy of the current network. On each of the networks created, the nodes in the corresponding oscillating component and the nodes downstream of this component are marked. The marked nodes cannot be reduced at any later step of the algorithm (i.e, they have their state undetermined in the quasi-attractors that are derived from these networks).

6. For the oscillating components of two nodes (i.e, only one normal node and its corresponding complementary node), check if any node downstream of these oscillating motifs participates in a stable motif with no composite nodes. If any of them do, go to step 7; otherwise, check if there are any stable motifs that are downstream of these oscillating components (these stable motifs would necessarily have a composite node). If there are not, go to step 7; if there are, check if any of them is downstream of a stable motif that is itself not downstream of any of these oscillating components. If this is the case, go to step 7; if this is not the case, then create one copy of the current network and mark the nodes in the oscillating motifs considered in this step and the nodes downstream of them. The marked nodes cannot be reduced at any later step of the algorithm (i.e, they have their state undetermined in the quasi-attractors that are derived from these networks).

7. Repeat 2-6 for each of the networks iteratively until no more stable motifs are found. The result, a set of fixed state nodes and their node state, and a set of nodes with undetermined states with their reduced Boolean functions, is the set of quasi-attractors (S2 Text subsection A.4).

8. Prune the set of quasi-attractors of duplicates (two quasi-attractors are the same if they have the same set of fixed state nodes and the same node state for these fixed-state nodes; if two quasi-attractors are the same, except that one of them has some nodes marked while the other one does not, remove the one that has the marked nodes).

Some of the resulting quasi-attractors have marked nodes while others do not. For every unmarked quasi-attractor there necessarily is a corresponding attractor in the Boolean network. For a marked quasi-attractor there may not be a corresponding attractor in the Boolean network; only by knowing the specific states visited during oscillations by the undetermined nodes in the quasi-attractor's reduced network can one confirm whether there is a corresponding attractor (this is a consequence of incomplete oscillations and unstable oscillations, see S2 Text subsection A.5).

7. *Conservation of attractors by the expanded network/network reduction attractor-finding method*

The first proposition states that the stable motifs found from the expanded network are such that the corresponding states of these motifs are partial fixed points of the Boolean rules of the nodes involved.

Proposition 1. *Let* $M = (V_{m_1}, V_{m_2}, \ldots, V_{m_l}, V_{m_{l+1}}, V_{m_{l+2}}, \ldots, V_{m_L})$ *be a stable motif in the expanded network representation, where* $V_{m_1}, V_{m_2}, \ldots, V_{m_l}$ *can either be a normal node or a complementary node, and where* $V_{m_{l+1}}, V_{m_{l+2}}, \ldots, V_{m_L}$ *are composite nodes. We denote* $M_{state} = (\sigma_{m_1} = b_{m_1}, \sigma_{m_2} = b_{m_2}, \ldots, \sigma_{m_l} = b_{m_l})$*, with* $b_{m_j} \in \{0, 1\}$ *as the corresponding state of* M *in the network state* Σ*:* $b_{m_j} = 1$ *if it is a normal node, and* $b_{m_j} = 0$ *if it is a complementary node. Then, for any normal node* v_{m_j} *or complementary node* $\overline{v}_{m_j}$ *in* M *and for any network state* Σ_M *such that* $\sigma_{m_k} = b_{m_k} \forall m_k \in \{m_1, m_2, \ldots, m_l\}$*, we have* $f_{m_j}(\Sigma_M) = b_{m_j}$*.*

The reverse of this proposition is also true, that is, if for a given set of node states updating any of the states in the set gives back the same state, regardless of the state of any node outside of the set, then this set of states correspond to a set of stable motifs in the expanded network representation:

Proposition 2. *Let* $M_{state} = (\sigma_{m_1} = b_{m_1}, \sigma_{m_2} = b_{m_2}, \ \ldots, \sigma_{m_l} = b_{m_l})$ *be the state of a set of nodes such that if* Σ_M *is any network state in which* $\sigma_{m_k} = b_{m_k} \forall m_k \in \{m_1, m_2, \ldots, m_l\}$*, then* $f_{m_j}(\Sigma_M) = b_{m_j}$*. Then (i) there is a set of stable motifs* $\{M_n\}$ *in the expanded network representation such that each of the* M_n*'s contain only normal nodes or complementary nodes of the nodes whose state is specified in* M_{state} *(normal nodes if* $b_{m_k} = 1$*, and complementary nodes if* $b_{m_k} = 0$*) and in which all other nodes in the* M_n*'s (if any) are composite nodes made up of the normal nodes or complementary nodes in the corresponding* M_n*, and (ii) the nodes whose state is specified in* M_{state} *but that are not included in the set of stable motifs* $\{M_n\}$ *are downstream of the nodes in at least one of the stable motifs.*

For the next propositions we need certain properties of the attractors of the general asynchronous updating scheme, in which the state of one randomly (uniformly) chosen node is updated at every discrete time step (see Methods). For any attractor $\mathcal{A}$, we can divide the N nodes into two classes: those that take the same value in all network states of $\mathcal{A}$ (i.e, either 0 or 1), and those that take more than one value in the different network states of $\mathcal{A}$ (i.e, both 0 and 1). We refer to the former as *stabilized or fixed-state nodes*, and to the latter as *oscillating nodes*. The following propositions state that fixed-state nodes can have inputs from fixed-state nodes or oscillating nodes (Proposition 3), while oscillating nodes must have at least one oscillating node as an input (Proposition 4).

Proposition 3. *Let* $\mathcal{A}$ *be an attractor of the Boolean network* (V, Σ, F) *under the general asynchronous updating scheme, and let* $\mathcal{S}$ *and* $\mathcal{O}$ *be the set of the fixed-state and oscillating nodes in the attractor, respectively. If* $v_s \in \mathcal{S}$*, and* b_s *is the fixed-state state of node* v_s*, then one of the following two cases holds: (i) one of the conjunctive clauses of* f_s *(if* $b_s = 1$*) or* $\overline{f}_s$ *(if* $b_s = 0$*) depends only on the specific state of the nodes of* $\mathcal{S}$ *in* $\mathcal{A}$*. If (i) is not true, then (ii) for both* f_s *and* $\overline{f}_s$ *at least one conjunctive clause depends on the state of one or more nodes in* $\mathcal{O}$ *and, if the clause depends on any more states, they have to be the state of the nodes of* $\mathcal{S}$ *in* $\mathcal{A}$*.*

Proposition 4. *Let* $\mathcal{A}$ *be an attractor of the Boolean network* (V, Σ, F) *under the general asynchronous updating scheme, and let* $\mathcal{S}$ *and* $\mathcal{O}$ *be the set of the fixed-state and oscillating nodes, respectively. If* $v_o \in \mathcal{O}$ *then (i) neither* f_o *nor* $\overline{f}_o$ *can have any conjunctive clauses that depend only on the state of the nodes of* $\mathcal{S}$ *in* $\mathcal{A}$ *(i.e, on* σ_s *if* $b_s = 1$*, or* $\overline{\sigma}_s$ *if* $b_s = 0$*), and (ii) both* f_o *and* $\overline{f}_o$ *must have at least one conjunctive clause that depends on the state of one or more nodes in* $\mathcal{O}$ *and, if this same clause depends on any other states, they must be the states of nodes of* $\mathcal{S}$ *in* $\mathcal{A}$*.*

We now reproduce the three lemmas that allow us to show that the reduction method conserves all attractors. In Lemma 1 we construct the set of nodes, for an arbitrary attractor, whose state are identified by our attractor-finding method, $\mathcal{S}_{red} \subset \mathcal{S}$. We also show that there is at least one stable motif composed of the corresponding states in the attractor of the nodes of $\mathcal{S}_{red}$ (as long as $\mathcal{S}_{red}$ is not empty). In Lemma 2 we show that the network reduction of these stable motifs can only fix the state of nodes in $\mathcal{S}_{red}$. In Lemma 3 we show that when no stable motifs are found, which is the exit condition in the attractor-finding algorithm (step 7, S2 Text subsection A.6)), the fixed-state nodes in an attractor $\mathcal{A}$ must be downstream of an oscillating motif.

For completeness, we reproduce how $\mathcal{S}_{red} \subset \mathcal{S}$ is constructed for a Boolean network attractor $\mathcal{A}$. Without loss of generality we can do a change of variables so that $\sigma_s = 1$ if $v_s \in \mathcal{S}$. By Proposition 3, we can divide $\mathcal{S}$ into the nodes

that have at least one conjunctive clause in their rule that depends only on the specific state of nodes of $\mathcal{S}$ in $\mathcal{A}$, and their complement in $\mathcal{S}$. We refer to the former as $\mathcal{S}_0$ and to the latter as $\mathcal{S}_{osc}$. Let $\mathcal{S}_1 \subset \mathcal{S}_0$ be the nodes that have at least one conjunctive clause in their rules that depends only on the specific state of the nodes of $\mathcal{S}_0$ in $\mathcal{A}$ (i.e, on σ_s, because of the change of variables). Let $\mathcal{S}_2 \subset \mathcal{S}_1$ be the nodes that have at least one conjunctive clause in their rules that depends only on the specific state of nodes of $\mathcal{S}_1$ in $\mathcal{A}$ (note they could depend on the states of nodes in $\mathcal{S}_0 - \mathcal{S}_1$). We do this iteratively until $\mathcal{S}_{i_{max}+1} = \mathcal{S}_{i_{max}}$ and denote $\mathcal{S}_{red} = \mathcal{S}_{i_{max}} \subset \mathcal{S}_0$. Since $\mathcal{S}_{red}$ was constructed by first removing the nodes that required nodes in $\mathcal{O}$ to have their states fixed, and then removing the ones that depended on the previously reduced nodes, and so on, then $\mathcal{S}_{red}$ corresponds to the set of nodes in $\mathcal{S}$ that do not depend in any way on nodes of $\mathcal{O}$ to have their node state fixed in their state on $\mathcal{A}$.

Lemma 1. *Let $\mathcal{A}$ be an attractor of the Boolean network (V, Σ, F) under the general asynchronous updating scheme, and let $\mathcal{S}$ and $\mathcal{O}$ be the set of the fixed-state and oscillating nodes of $\mathcal{A}$, respectively. There exists a set of nodes $\mathcal{S}_{red} \subset \mathcal{S}$ such that in the expanded network representation of (V, Σ, F) there is at least one stable motif composed only of the corresponding states of the nodes of $\mathcal{S}_{red}$ in $\mathcal{A}$, or composite nodes composed of such nodes.*

Lemma 2. *Let $\mathcal{S}_{red} \subset \mathcal{S}$ be the constructed set of nodes in Lemma 1. Then (i) $\mathcal{S}_{red}$ is such that the network reduction of any stable motif composed only of the corresponding states of $\mathcal{S}_{red}$ in $\mathcal{A}$ (or composite nodes composed of such nodes) can only fix the state of nodes in $\mathcal{S}_{red}$, and (ii) if any of the states of the nodes in $\mathcal{S}_{red}$ is fixed by network reduction, then it has to be on their corresponding state in $\mathcal{A}$; if they do not have their state fixed, then either their rule (if their fixed state in $\mathcal{A}$ is 1) or the negation of their rule (if their fixed state is 0) in the reduced network have a conjunctive clause that only depends on the specific state of the nodes of $\mathcal{S}_{red}$ in $\mathcal{A}$ (i.e, on σ_s if $b_s = 1$, or $\overline{\sigma}_s$ if $b_s = 0$) that did not have their states fixed during network reduction.*

Lemma 3. *Let $\mathcal{A}$ be an attractor of the Boolean network (V, Σ, F) under the general asynchronous updating scheme, and let $\mathcal{S}$ and $\mathcal{O}$ be the set of the fixed-state and oscillating nodes, respectively. Let $\mathcal{S}_{red} \subset \mathcal{S}$ be the constructed set of nodes in Lemma 1 and assume that $\mathcal{S}_{red}$ is empty and that $\mathcal{O}$ is a non empty set. Then the expanded network representation of (V, Σ, F) must be such that the normal nodes and complementary nodes of the elements in $\mathcal{O}$, and the nodes corresponding to the state of the nodes of $\mathcal{S}$ in $\mathcal{A}$ must both be downstream of an oscillating motif that contains at least one of the nodes in $\mathcal{O}$.*

The following theorem is the main result of this section, and it combines the results of Lemma 1, 2, and 3. It shows that for every attractor (under general asynchronous updating, see Methods) in the network, our attractor-finding method finds a corresponding quasi-attractor in which the state of the nodes in $\mathcal{S}_{red}$ is the same as in the attractor, and in which the rest of the nodes are either be part of an oscillating motif or downstream of it.

Theorem 1. *Let $\mathcal{A}$ be an attractor of the Boolean network (V, Σ, F) under the general asynchronous updating scheme, and let $\mathcal{S}$ and $\mathcal{O}$ be the set of the fixed-state and oscillating nodes, respectively. Let $\mathcal{S}_{red} \subset \mathcal{S}$ be the set of nodes constructed in Lemma 1. Then, there exists a set of stable motifs such that, by applying network reduction, all the nodes in $\mathcal{S}_{red}$ get fixed in their steady state in $\mathcal{A}$, while the rest of the nodes in V are part of the final reduced network. This resulting final reduced network is such that, in its expanded network representation, all the nodes are either be part of an oscillating motif containing at least one of the nodes in $\mathcal{O}$, or be downstream of an oscillating motif.*

B. The stable motif control method

1. *A sequence of stable motifs uniquely determines an equivalence class of attractors*

The main step in proving that our stable motif control method works is to show that a sequence of stable motifs obtained from the attractor-finding method uniquely determines an attractor. Since the attractor-finding method yields quasi-attractors, we need to be more precise with what "uniquely determines an attractor" refers to in this context. Let (V, Σ, F) be a Boolean network, and let $\mathbb{A} = \{\mathcal{A}_i\}, i = 1, 2, \ldots, n_{\mathbb{A}}$ be the set of general asynchronous attractors of (V, Σ, F). We define $\mathbb{A}^{(red)} = \{\mathcal{A}_j^{(red)}\}, j = 1, 2, \ldots, n_{\mathbb{A}^{(red)}}$ as the partition of the attractors $\mathbb{A}$ generated by the equivalence relation $\sim$, where $\mathcal{A}_k \sim \mathcal{A}_l$ if the $\mathcal{S}_{red}$ for $\mathcal{A}_k$ (as defined in S2 Text subsection A.7) is the same as the $\mathcal{S}_{red}$ for $\mathcal{A}_l$, and the state of each node $v \in \mathcal{S}_{red}$ is the same in both $\mathcal{A}_k$ and $\mathcal{A}_l$. Consequently, each $\mathcal{A}_i \in \mathbb{A}$ is an element of only one $\mathcal{A}_j^{(red)} \in \mathbb{A}^{(red)}$ (since it is a partition generated by an equivalence relation), and $\forall \mathcal{A}_k, \mathcal{A}_l \in \mathcal{A}_j^{(red)}$, we have $\mathcal{A}_k \sim \mathcal{A}_l$.

Using the above we can now be more precise: For a Boolean network (V, Σ, F) under general asynchronous updating, a sequence of stable motifs obtained from the attractor-finding method uniquely determines an equivalence class of attractors $\mathcal{A}^{(red)}$, each of which is the set of all attractors of (V, Σ, F) that share the same $\mathcal{S}_{red}$ and the state of each

node in $\mathcal{S}_{red}$ ($\mathcal{S}_{red}$ corresponds to the set of fixed-state nodes in an attractor $\mathcal{A}$ that do not depend in any way on the state of nodes whose state oscillates in $\mathcal{A}$ to have their node state fixed in their state on $\mathcal{A}$). We prove this below.

Lemma 4. *Let $\mathcal{B} = (V, \Sigma, F)$ be a Boolean network, let $\mathbb{A}^{(red)}$ be the set of equivalence classes of attractors defined above, and let S_{red} and $S_{red,\Sigma}$ denote, respectively, the set of nodes and node states which define an equivalence class of attractors $\mathcal{A}^{(red)} \in \mathbb{A}^{(red)}$. Let $\mathcal{S}_{seq} = (\mathcal{M}_1, \ldots, \mathcal{M}_L)$ be a sequence of stable motifs of the Boolean network obtained by the attractor-finding method (section A), let Q be its associated quasi-attractor, and let $\mathcal{S}_Q$ and Q_Σ be the set of fixed-state nodes in Q and the state of the fixed-state nodes in Q, respectively. Then $\mathcal{S}_Q$ and Q_Σ are such that $\mathcal{S}_Q = \mathcal{S}_{red}$ and $Q_\Sigma = S_{red,\Sigma}$ for only one $\mathcal{A}^{(red)} \in \mathbb{A}^{(red)}$.*

Proof. Let $\mathcal{B}_i, i = 1, 2, \ldots, L$ be the reduced Boolean network obtained from the Boolean network $\mathcal{B}$ after applying network reduction up to and including the stable motif $\mathcal{M}_i$ in the sequence $\mathcal{S}_{seq}$, and define $\mathcal{B}_0 \equiv \mathcal{B}$. By construction, one of the stable motifs of the Boolean network $\mathcal{B}_i$ is $\mathcal{M}_{i+1}$. Let $\mathcal{R}_i, i = 1, 2, \ldots, L$ be the node state of the nodes in the Boolean network $\mathcal{B}_{i-1}$ whose node state becomes fixed after applying network reduction with motif $\mathcal{M}_i$. By the definition of quasi-attractor Q, $\mathcal{S}_Q$ is given by the set of nodes whose state is specified in $\mathcal{M}_i$ or $\mathcal{R}_i$, and Q_Σ is given by the nodes states specified in $\mathcal{M}_i$ or $\mathcal{R}_i$, that is,

$$Q_\Sigma \equiv \bigcup_{j=1}^{L} \mathcal{M}_j \cup \mathcal{R}_j = \left\{ \sigma_{q_1} = b_{q_1}, \sigma_{q_2} = b_{q_2}, \ldots, \sigma_{q_{n_Q}} = b_{q_{n_Q}} \right\},$$
$$\mathcal{S}_Q = \left\{ v_{q_1}, v_{q_2}, \ldots, v_{q_{n_Q}} \right\}.$$

Let $\mathcal{A}^{(red)} \in \mathbb{A}^{(red)}$, and let $\mathcal{S}_{red}$ and $\mathcal{S}_{red,\Sigma}$ be the set of fixed-state nodes and the state of the fixed-states nodes, respectively, which define the equivalence class $\mathcal{A}^{(red)}$. Let $\mathbb{A}_Q^{(red)} \subset \mathbb{A}^{(red)}$ be all the equivalence classes of attractors $\mathcal{A}^{(red)} \in \mathbb{A}^{(red)}$ for which their defining $\mathcal{S}_{red}$ and $\mathcal{S}_{red,\Sigma}$ satisfy $\mathcal{S}_Q \subseteq \mathcal{S}_{red}$ and $Q_\Sigma \subseteq \mathcal{S}_{red,\Sigma}$. Note that $\mathbb{A}_Q^{(red)}$ cannot be an empty set; this is a consequence of stable motifs being partial fixed points of the dynamics (Proposition 1), and the finite size of the state space spanned by all Σ which satisfy $\sigma_{q_i} = b_{q_i} \forall v_{q_i} \in \mathcal{S}$.

Let $\mathcal{A}'^{(red)} \in \mathbb{A}_Q^{(red)}$ and let $\mathcal{S}'_{red} \neq \mathcal{S}_Q$ and $\mathcal{S}'_{red,\Sigma}$ its defining set of fixed-state nodes and node states. We now show, by contradiction, that $\mathcal{S}'_{red} \equiv \mathcal{S}_Q$. Lemma 2 (with $\mathcal{S}'_{red}$) guarantees that network reduction of the stable motifs in $\mathcal{S}_{seq}$ can only fix the state of nodes in $\mathcal{S}_Q$ on their corresponding state in Q_Σ, since $\mathcal{S}_Q \subseteq \mathcal{S}'_{red}$ and $Q_\Sigma \subseteq \mathcal{S}'_{red,\Sigma}$. Lemma 2 also guarantees that each node $v_i \in \mathcal{S}'_{red} - \mathcal{S}_Q$ has a conjunctive clause in its Boolean function in $\mathcal{B}_L$ if $\sigma_i = 1$ in $\mathcal{S}'_{red,\Sigma}$, or in the negation of their Boolean function if $\sigma_i = 0$ in $\mathcal{S}'_{red,\Sigma}$, that only depends on the specific state of the nodes of $\mathcal{S}'_{red} - \mathcal{S}_Q$ in $\mathcal{S}'_{red,\Sigma}$.

From the above, Lemma 2 implies that each node $v_i \in \mathcal{S}'_{red} - \mathcal{S}_Q$ has an associated normal node (if $\sigma_i = 1$ in $\mathcal{S}'_{red,\Sigma}$) or complementary node (if $\sigma_i = 0$ in $\mathcal{S}'_{red,\Sigma}$) in the expanded network representation of $\mathcal{B}_L$ with, at least, one expanded network input node v_j, where v_j is either (a) the associated normal node of a node in $\mathcal{S}'_{red} - \mathcal{S}_Q$ whose node state is $\sigma_j = 1$ in $\mathcal{S}'_{red,\Sigma}$, (b) the associated complementary node of a node in $\mathcal{S}'_{red} - \mathcal{S}_Q$ whose node state is $\sigma_j = 0$ in $\mathcal{S}'_{red,\Sigma}$, or (c) a composite node with only nodes in (a) and/or (b) as input nodes. Consequently , the expanded network representation of $\mathcal{B}_L$ must have a stable motif composed only of expanded network nodes v_j satisfying either (a), (b) and (c). But this is not possible, since $\mathcal{B}_L$ has no stable motifs (if it had, then it would be part of the sequence $\mathcal{S}_{seq}$). By contradiction, we must have $\mathcal{S}'_{red} \equiv \mathcal{S}_Q$.

From the previous paragraph we have $\mathcal{S}'_{red} \equiv \mathcal{S}_Q$. This implies that $\mathbb{A}_Q^{(red)} \subset \mathbb{A}^{(red)}$ is composed of a single equivalence class of attractors $\mathcal{A}^{(red)}$, which has $\mathcal{S}_{red} = \mathcal{S}_Q$ and $\mathcal{S}_{red,\Sigma} = Q_\Sigma$, thus concluding our proof. □

2. *Fixing the node states specified by a sequence of stable motifs*

Lemma 4 shows that a sequence of stable motifs uniquely determines an equivalence class of attractors, but this does not directly show the result of fixing the node states specified by a sequence of stable motifs. For this, we first need to define what we mean with a Boolean network in which a set of node states is fixed. For a Boolean network $\mathcal{B} = (V, \Sigma, F)$ under general asynchronous updating and a set of node states $P = \{\sigma_{p_1} = b_{p_1}, \sigma_{p_2} = b_{p_2}, \ldots, \sigma_{p_l} = b_{p_l}\}$, we denote $\mathcal{B}_P = (V, \Sigma, F')$, with $f'_i \in F'$ such that $f'_i = f_i|_P$ if $i \notin \{p_1, \ldots, p_l\}$ or $f'_i = b_i$ if $i \in \{p_1, \ldots, p_l\}$, as the Boolean network in which P is fixed.

Note that, formally, $\sigma_{p_i} \neq b_{p_i}$ for any $\sigma_{p_i} \in P$ is an allowed state of Σ in the Boolean network $\mathcal{B}_P$. However, no attractors in $\mathcal{B}_P$ have $\sigma_{p_i} \neq b_{p_i}$ for any $\sigma_{p_i} \in P$ since $f'_i = b_i, \forall i \in \{p_1, \ldots, p_l\}$. Furthermore, if we restrict ourselves to the substate space of $\mathcal{B}_P = (V, \Sigma, F')$ with $\sigma_i = b_i, \forall \sigma_i \in P$, it can be shown that this substate space

is equivalent to having taken $\mathcal{B}$, restricting it to $\sigma_i = b_i, \forall \sigma_i \in P$, and removing the transitions from network states with $\sigma_i = b_i, \forall \sigma_i \in P$ to network states with $\sigma_i \neq b_i$ for at least one $\sigma_i \in P$. We choose to work with $\mathcal{B}_P$ instead of a restricted $\mathcal{B}$ because of its similarity with the network reduction process of the attractor-finding method.

We now show that for a sequence of stable motifs S_{seq} with associated quasi-attractor Q and fixed-node states Q_Σ, the Boolean network in which Q_Σ is fixed has the same attractors as the equivalence class of attractors specified by S_{seq}. For this, we use a more general statement for which the above is a special case.

Proposition 5. *Let $\mathcal{S}_{seq} = (\mathcal{M}_1, \mathcal{M}_2, \ldots, \mathcal{M}_L)$ be a sequence of stable motifs of $\mathcal{B} = (V, \Sigma, F)$ obtained by the attractor-finding method, and let $\mathcal{B}_\lambda, \lambda \in \{1, 2, \ldots, L\}$ be the reduced Boolean network obtained from $\mathcal{B}$ after applying network reduction up to and including the stable motif $\mathcal{M}_\lambda$ in the sequence $\mathcal{S}_{seq}$. Let $Q_{\Sigma,\lambda}$ be the following set of node states*

$$Q_{\Sigma,\lambda} = \bigcup_{j=1}^{\lambda} \mathcal{M}_j \cup \mathcal{R}_j,$$

where $\mathcal{R}_i, i = 1, 2, \ldots, L$ is defined in the proof of Lemma 4. Then, the attractors in the reduced network $\mathcal{B}_\lambda$ are the same as the attractors of the Boolean network $\mathcal{B}_{Q_{\Sigma,\lambda}} = (V, \Sigma, F')$ when comparing only the state of nodes present in both $\mathcal{B}_\lambda$ and $\mathcal{B}_{Q_{\Sigma,\lambda}}$, and the nodes v_i present only in $\mathcal{B}_{Q_{\Sigma,\lambda}}$ are such that their state in all attractors is given by $\sigma_i = b_i, \sigma_i \in Q_{\Sigma,\lambda}$.

Let us sketch the proof for this proposition. By construction, the Boolean function f_i' of node v_i in $\mathcal{B}_{Q_{\Sigma,\lambda}}$ is the same as the Boolean function of node v_i of $\mathcal{B}_\lambda$ if v_i is present in both $\mathcal{B}_{Q_{\Sigma,\lambda}}$ and $\mathcal{B}_\lambda$; this is because the Boolean functions of a reduced network are given by

$$f_i|_{\mathcal{M}_1 \cup \mathcal{R}_1}|_{\mathcal{M}_2 \cup \mathcal{R}_2}| \ldots |_{\mathcal{M}_\lambda \cup \mathcal{R}_\lambda} = f_i|_{Q_{\Sigma,\lambda}} \equiv f_i'.$$

The nodes present in $\mathcal{B}_{Q_{\Sigma,\lambda}}$ but not in $\mathcal{B}_\lambda$ are the nodes whose state is specified in $Q_{\Sigma,\lambda}$. The Boolean function of each of these nodes is $f_i' = b_i$, where b_i is specified in $Q_{\Sigma,\lambda}$. Since this implies that there is always a transition from any network state with at least one $\sigma_i \neq b_i, \sigma_i \in Q_{\Sigma,\lambda}$ to a network state with $\sigma_i = b_i, \sigma_i \in Q_{\Sigma,\lambda}$, but not the other way around, an attractor of $\mathcal{B}_{Q_{\Sigma,\lambda}}$ must have $\sigma_i = b_i, \forall \sigma_i \in Q_{\Sigma,\lambda}$. Since the functions of all nodes present in both $\mathcal{B}_{Q_{\Sigma,\lambda}}$ and $\mathcal{B}_\lambda$ are the same, and the attractors of $\mathcal{B}_{Q_{\Sigma,\lambda}}$ must have $\sigma_i = b_i, \forall \sigma_i \in Q_{\Sigma,\lambda}$, then the attractors in the reduced network $\mathcal{B}_\lambda$ must be the same as the attractors of the Boolean network $\mathcal{B}_{Q_{\Sigma,\lambda}}$ when comparing only the state of nodes present in both $\mathcal{B}_\lambda$ and $\mathcal{B}_{Q_{\Sigma,\lambda}}$.

Using Lemma 4, Proposition 5, and the fact that $Q_{\Sigma,\lambda=L} \equiv Q_\Sigma$ (as defined in Proposition 5 and Lemma 4, respectively), we can prove that the attractors of the Boolean network $\mathcal{B}_{Q_\Sigma}$ are the same as the attractors in the equivalence class defined by quasi-attractor Q.

Proposition 6. *Let $\mathcal{S}_{seq} = (\mathcal{M}_1, \ldots, \mathcal{M}_L)$ be a sequence of stable motifs of $\mathcal{B} = (V, \Sigma, F)$ obtained by the attractor-finding method, let Q be its associated quasi-attractor, and let $\mathcal{S}_Q$ and Q_Σ be the set of fixed-state nodes in Q and the state of the fixed-state nodes in Q, respectively. Let $\mathcal{A}^{(red)}$ be the equivalence class of attractors with $\mathcal{S}_{red} = \mathcal{S}_Q$ and $\mathcal{S}_{red,\Sigma} = Q_\Sigma$ given by Lemma 4. Then, the attractors of the Boolean network $\mathcal{B}_{Q_\Sigma} = (V, \Sigma, F')$ are the same as the attractors in $\mathcal{A}^{(red)}$.*

Before proceeding, we want to emphasize the role of Lemma 4, Proposition 5, and Proposition 6 in proving that the stable motif control algorithm works. Lemma 4 is the main result of section B, and shows that a sequence of stable motifs $\mathcal{S}_{red}$ uniquely determines an equivalence class of attractors $\mathcal{A}^{(red)}$. Proposition 6 shows that the Boolean network obtained by fixing the node states specified by $\mathcal{S}_{red}$ has the attractors in $\mathcal{A}^{(red)}$ as its only attractors, and is a direct consequence of Lemma 4 and the attractor-finding method (section A). Lemma 4 and Proposition 6 guarantee the effectiveness of the stable motif control algorithm: each sequence of stable motifs $\mathcal{S}_{red}$ obtained from the attractor-finding method singles out one equivalent class of attractors $\mathcal{A}^{(red)}$ (Lemma 4), and Boolean network obtained by fixing the node states specified by $\mathcal{S}_{red}$ has the attractors in $\mathcal{A}^{(red)}$ as its only attractors (Proposition 6).

Proposition 6 shows that the attractors of the reduced Boolean networks obtained using a shortened subsequence of $\mathcal{S}_{red}$ are equivalent to the attractors of the Boolean network obtained by fixing the node states specified by this shortened subsequence. This allows us to consider only the attractors of reduced Boolean networks when showing that a sequence simplified by steps 2 and 3 of the stable motif control algorithm (which we reproduce below) has the same effect as the full sequence $\mathcal{S}_{red}$.

3. *Simplifying the sequences of stable motifs*

For completeness, we reproduce the stable motif control algorithm (see Methods and S7 Text for more details):

- *Step 1*: Identify the sequences of stable motifs that lead to $\mathcal{A}$. These can be obtained from the stable motif succession diagram (see Fig. 2) by choosing the attractor of interest in the right-most part and selecting all of the attractor's predecessors in the succession diagram.

- *Step 2*: Shorten each sequence $\mathcal{S} \in Sequences$ by identifying the minimum number of motifs in $\mathcal{S}$ required for reaching $\mathcal{A}$ and removing the remaining motifs from the sequence. This minimum number of motifs can be identified from the stable motif succession diagram (Fig. 2); they are the motifs after which all consequent motif choices lead to the same attractor $\mathcal{A}$.

- *Step 3*: For each stable motif state $\mathcal{M} = (\sigma_{m_1} = b_{m_1}, \sigma_{m_2} = b_{m_2}, \ldots, \sigma_{m_l} = b_{m_l})$ corresponding to node v, find the subsets of stable motif's states $O = \{M_i\}, M_i \subseteq \mathcal{M}$ that, when fixed in the logical model, are enough to force the state of the whole motif into $\mathcal{M}$. At worst, there will only be one subset, which will equal the whole stable motif's state $\mathcal{M}$. If any of these subsets is fully contained in another subset, remove the larger of the subsets. In each stable motif sequence $\mathcal{S} = (\mathcal{M}_1, \ldots, \mathcal{M}_L)$, substitute every stable motif $\mathcal{M}_j$ with the subsets of the stable motif states obtained, that is, $\mathcal{S} = (O_1, \ldots, O_L)$.

- *Step 4*: For each sequence $\mathcal{S} = (O_1, \ldots, O_L)$ create a set of states $\mathcal{C}$ by choosing one of the subsets of stable motif's states M_{k_j} in each O_j and taking their union, that is, $\mathcal{C} = M_{k_1} \cup \cdots \cup M_{k_L}, M_{k_j} \in O_j$. The network control set for attractor $\mathcal{A}$ is the set of node states $C_{\mathcal{A}} = \{\mathcal{C}_i\}$ obtained from all possible combinations of subsets of stable motif's states M_{k_j}'s for every sequence $\mathcal{S}$. To avoid any redundancy, we additionally prune $C_{\mathcal{A}}$ of duplicates and remove each set of node states $\mathcal{C}_i$ which is a superset of any of the other sets of node states $\mathcal{C}_j$ (i.e. $\mathcal{C}_j \subset \mathcal{C}_i$).

To justify that step 2 of the stable motif control algorithm yields a simplified sequence that leads to the same equivalence class of attractors as the original sequence, it suffices to show that a Boolean network with a stable motif succession diagram in which each sequence of stable motifs leads to the same equivalence class of attractors $\mathcal{A}^{(red)}$ has $\mathcal{A}^{(red)}$ as its only equivalence class of attractors.

Proposition 7. *Let $\mathcal{B} = (V, \Sigma, F)$ be a Boolean network in which all sequences of stable motifs obtained from the attractor-finding method lead to the same equivalence class of attractors $\mathcal{A}^{(red)}$. Then, $\mathcal{A}^{(red)}$ is the only equivalence class of attractors in $\mathcal{B}$.*

Proof. By Theorem 1, every attractor $\mathcal{A}$ has a sequence of stable motifs for which the attractor-finding method fixes all the nodes in the $\mathcal{S}_{red}$ obtained from $\mathcal{A}$ to their fixed state in $\mathcal{A}$. Let $\mathcal{A}'$ be an attractor not in the equivalence class $\mathcal{A}^{(red)}$. Then, Theorem 1 guarantees that there must be a sequence of stable motifs that lead to the equivalence class specified by $\mathcal{A}'$. But, this is a contradiction, since all sequences of stable motifs lead to the equivalence class of attractors $\mathcal{A}^{(red)}$. Hence, $\mathcal{A}^{(red)}$ is the only equivalence class of attractors in $\mathcal{B}$. □

To justify step 3 of the stable motif control algorithm we need to show that, given a Boolean network with a stable motif $\mathcal{M}$, the Boolean network obtained by fixing the state of the nodes specified in the stable motif $\mathcal{M}$ has the same attractors as the the Boolean network obtained by fixing the state of the nodes given by the subsets of $\mathcal{M}$ specified by step 3.

Proposition 8. *Let $\mathcal{B} = (V, \Sigma, F)$ be a Boolean network, let $\mathcal{M}$ be a stable motif of $\mathcal{B}$, and let $\mathcal{B}_{\mathcal{M}}$ be the Boolean network $\mathcal{B}$ with the node states specified in $\mathcal{M}$ fixed. Let $\mathcal{M}' \subset \mathcal{M}$ be a set of node states such that the network reduction of $\mathcal{B}$ using $\mathcal{M}'$ and network reduction of network reduction of $\mathcal{B}$ using $\mathcal{M}$ yield the same reduced network $\mathcal{B}_{red}$, and let $\mathcal{B}_{\mathcal{M}'}$ be the Boolean network $\mathcal{B}$ with the node states specified in $\mathcal{M}'$ fixed. Then, the attractors in $\mathcal{B}_{\mathcal{M}}$ are the same as the attractors of the Boolean network $\mathcal{B}_{\mathcal{M}'}$.*

Let us sketch the proof of this proposition. Since $\mathcal{M}' \subset \mathcal{M}$ yields the same reduced network as $\mathcal{M}$ then $f_i|_{\mathcal{M}'} = b_i$ for a set of σ_i's, which we denote $\mathcal{M}'_1$, such that $\sigma_i \in \mathcal{M} - \mathcal{M}'$. If $\mathcal{M} - \mathcal{M}' - \mathcal{M}'_1$ is not empty, we can follow the same reasoning, and find $f_i|_{\mathcal{M}'}|_{\mathcal{M}'_1} = b_i$ for a set of σ_i's, which we denote $\mathcal{M}'_2$, such that $\sigma_i \in \mathcal{M} - \mathcal{M}' - \mathcal{M}'_1$. If $\mathcal{M} - \mathcal{M}' - \mathcal{M}'_1 - \mathcal{M}'_2$ is not empty, and we follow the same reasoning, we can do this iteratively until $\mathcal{M} - \mathcal{M}' - \mathcal{M}'_1 - \ldots - \mathcal{M}'_m$ is empty. Defining $\mathcal{M}'_0 = \mathcal{M}'$, the result is a group of $\mathcal{M}'_i, i = 0, 1, \ldots, m$, which are such that

$$\mathcal{M} - \mathcal{M}'_0 - \mathcal{M}'_1 - \ldots - \mathcal{M}'_j = \mathcal{M}''_j \neq \varnothing,\ j = 0, 1, \ldots, m-1$$
$$\mathcal{M} - \mathcal{M}'_0 - \mathcal{M}'_1 - \ldots - \mathcal{M}'_m = \mathcal{M}''_m = \varnothing,$$
$$f_i|_{\mathcal{M}'_0}|_{\mathcal{M}'_1}|\ldots|_{\mathcal{M}'_k} = b_i,\ \sigma_i \in \mathcal{M}'_{k+1},\ k = 0, 1, \ldots, m-1.$$

The Boolean network $\mathcal{B}_{\mathcal{M}'}$ has Boolean functions given by $f'_i = f_i|_{\mathcal{M}'}, \forall \sigma_i \notin \mathcal{M}'$ and $f'_i = b_i, \forall \sigma_i \in \mathcal{M}'$. This implies that there is always transition from any network state with at least one $\sigma_j \neq b_j, \sigma_j \in \mathcal{M}'$ to a network state with

$\sigma_j = b_j, \sigma_j \in \mathcal{M}'$, but not the other way around. This implies that the attractors in $\mathcal{B}_{\mathcal{M}'}$ must have $\sigma_j = b_j, \forall \sigma_j \in \mathcal{M}'$. Similarly, since $f_i' = f_i|_{\mathcal{M}'} = b_i, \forall \sigma_i \in \mathcal{M}_1'$, then the attractors in $\mathcal{B}_{\mathcal{M}'}$ must have $\sigma_j = b_j, \forall \sigma_j \in \mathcal{M}_1'$.

Since we are interested in the attractors, we can restrict ourselves to network states with $\sigma_j = b_j, \forall \sigma_j \in \mathcal{M}' \cup \mathcal{M}_1'$. The Boolean functions evaluated at network states with $\sigma_j = b_j \in \mathcal{M}' \cup \mathcal{M}_1'$ are equivalent to $f_i|_{\mathcal{M}_0'}|_{\mathcal{M}_1'}$, which implies $f_i|_{\mathcal{M}_0'}|_{\mathcal{M}_1'} = b_i, \forall \sigma_i \in \mathcal{M}_2'$ and that the attractors in $\mathcal{B}_{\mathcal{M}'}$ must have $\sigma_j = b_j, \forall \sigma_j \in \mathcal{M}_2'$. Doing this iteratively, we get that the attractors in $\mathcal{B}_{\mathcal{M}'}$ must have $\sigma_j = b_j, \forall \sigma_j \in \mathcal{M}_0' \cup \mathcal{M}_1' \cup \ldots \cup \mathcal{M}_m' \equiv \mathcal{M}$, and that the Boolean functions evaluated at the network states where the attractors can be ($\sigma_j = b_j, \forall \sigma_j \in \mathcal{M}$) are given by $f_i' = f_i|_{\mathcal{M}'}, \forall \sigma_i \notin \mathcal{M}$ and $f_i' = b_i, \forall \sigma_i \in \mathcal{M}$. Since these Boolean functions at the network states where the attractor of $\mathcal{B}_{\mathcal{M}'}$ can be are equivalent to the Boolean functions of $\mathcal{B}_{\mathcal{M}}$, then the attractors of both must be the same.
