# Supplementary material for: Cell Fate Reprogramming by Control of Intracellular Network Dynamics
Source: PLoS Comput Biol. 2015 Apr 7;11(4):e1004193. doi: 10.1371/journal.pcbi.1004193 (PMC4388852; doi:10.1371/journal.pcbi.1004193)
Supplement: S3 Text — (PDF) [file pcbi.1004193.s003.pdf]

Supporting Information

S3 TEXT. TIME COMPLEXITY AND MITIGATION TECHNIQUES FOR THE ATTRACTOR-FINDING METHOD AND THE STABLE MOTIF CONTROL APPROACH

In this part we discuss the time complexity of our methods, the worst case scenarios, and mitigation techniques for when our method takes a prohibitively long amount of time. This discussion is based on our experience with Boolean models of intracellular networks [S3] and random Boolean networks [31, S4]. For a detailed description of each step in the attractor-finding method see S2 Text and ref. [41]. For an algorithmic description of each step in the stable motif control approach see S7 Text .

In the following we use $V = (v_1, v_2, \ldots, v_N)$ to represent the N nodes of the Boolean network, $\sigma_i, i = 1, 2, \ldots, N$ to represent the state of node v_i, $\Sigma = (\sigma_1, \sigma_2, \ldots, \sigma_N)$ to represent the states of all nodes (also called a network state), $f_i, i = 1, 2, \ldots, N$ to represent the Boolean function of node v_i, and $F = (f_1, f_2, \ldots, f_N)$ to represent all the Boolean functions. We use $f(\Sigma)$ to denote a Boolean function f evaluated at a network state Σ, and $f|_P$ to denote a Boolean function where only the state of a subset of nodes $P = \{\sigma_{p_1}, \sigma_{p_2}, \ldots, \sigma_{p_l}\}$ is evaluated. We commonly use b_i to indicate that a specific value for node state σ_i is chosen, that is, $\sigma_i = b_i$.

A. Expanded network/network reduction attractor-finding method of ref. [41]

In this section we restrict ourselves to the steps of the attractor-finding method which we have found to be the most time consuming, namely, the creation of the expanded network representation and the identification of stable motifs from the expanded network representation.

1. *The expanded network representation*

The expanded network representation requires each Boolean function f to be written in a disjunctive normal form:

$$\begin{aligned} f = &(s_1 \text{ AND } s_2 \text{ AND } \cdots \text{ AND } s_k) \text{ OR } (s_{k+1} \text{ AND } s_{k+2} \text{ AND } \cdots s_l) \\ &\text{OR } \cdots \text{ OR } (s_m \text{ AND } s_{m+1} \text{ AND } \cdots \text{ AND } s_n)\,, \end{aligned}$$

where the s_j's are either the states of one of the input nodes of f, or one of these states' negations. Additionally, we require that if for M, denoting a state of a subset of the inputs of f, one has $f|_M = 1$ (regardless of the states of the remaining inputs), then the disjunctive form of f_i must have at least one of its conjunctive clauses equal to 1 when evaluated at the state M of this subset of nodes. In logic minimization terms, this is equivalent to requiring that f is written as the sum of all of its prime implicants [S5, S6, S7, S8].

The number of prime implicants of a Boolean function f of K inputs is known to be at most $O(3^K/\sqrt{K})$ [S9]. For a Boolean function that can be expressed as a disjunctive normal form of m conjunctive clauses, the number of prime implicants is bounded by $2^m - 1$ [S10]. The Boolean functions in the models we use have a relatively low number of inputs ($K \leq 10$), thus the simplest algorithm (the QuineMcCluskey algorithm [S5, S6, S7]) is sufficient to find the disjunctive normal form. If one requires Boolean functions with a larger number of inputs, more sophisticated algorithms should be used (e.g. [S8, S11] and references within).

2. *Identifying stable motifs from the expanded network*

A stable motif M in the expanded network is any of the smallest strongly connected components (SCCs) in the expanded network representation which satisfy these two properties:

1. If M contains a normal node v_i (complementary node $\overline{v}_i$) then M does not contain its corresponding complementary node $\overline{v}_i$ (normal node v_i).

2. If M contains a composite node $v^{(comp)}$, then all input nodes of $v^{(comp)}$ are elements of M.

The identification of stable motifs tends to be the most time-consuming part of our method. Specifically, it is the identification of stable motifs of the full network model that we have found to be the most time-consuming.

We are interested in identifying the smallest strongly connected components subject to two restrictions. Since most algorithms to identify stable connected components restrict themselves to the largest strongly connected components, we need to devise a way to enumerate all strongly connected components. To do this, we use the fact that any strongly connected component is composed of cycles. We first identify all directed cycles in the expanded network that do not contain both a normal node and its complementary node, which guarantees that property 1 of stable motifs is satisfied. For each of these cycles, we check whether it satisfies property 2; if it does, then the cycle is a stable motif.

For the cycles that do not satisfy property 2, we form all unions of cycles which share composite nodes, while discarding unions that do not satisfy property 1, until they satisfy property 2 for the composite node being considered. The result is a set of SCCs that satisfy property 1 and property 2 for at least one composite node inside the SCC. For each of these SCCs, we check whether it satisfies property 2 for all composite nodes; if it does, then this SCC is a stable motif candidate. For the SCCs that do not satisfy property 2 for all composite nodes, we repeat the same step as before, that is, we form all unions of SCCs which share composite nodes, while discarding the unions that do not satisfy property 1, until they satisfy property 2 for the composite node being considered. We do this iteratively, until only SCCs that satisfy property 1 and property 2 for all composite nodes are left. Finally, we take all SCCs obtained during the process that satisfy property 1 and 2, and leave only SCCs which do not have other SCCs as a subset, which yields the smallest SCCs that satisfy property 1 and 2, i.e., the stable motifs.

In the following we discuss the complexity of each step of the stable motif identification algorithm.

3. *Complexity of enumerating all cycles in the expanded network*

To identify stable motifs, we first search the expanded network $G_{exp} = (V_{exp}, E_{exp}, F_{exp})$ for all directed cycles that do not contain both a normal node and its complementary node using a modified version of Johnson's cycle algorithm [S12]. The original Johnson's cycle algorithm for a graph $G = (V, E)$ has a time complexity $O\left((|V| + |E|)(C + 1)\right)$, where $|V|$ is the number of nodes, $|E|$ is the number of edges, and C is the total number of directed cycles in G. In our modified version of Johnson's algorithm, a normal/complementary node can only be added to the stack from which cycles are obtained if its respective complementary/normal node is not already part of the stack. Consequently, this modified version of Johnson's cycle algorithm has a similar time complexity as the original algorithm, specifically, $O\left((|V_{exp}| + |E_{exp}|)(c + 1 + |V_{exp}| + |E_{exp}|)\right)$, where $|V_{exp}|$ is the number of nodes in G_{exp}, $|E_{exp}|$ is the number of edges in G_{exp}, and c is the number of directed cycles in G_{exp} that satisfy property 1 of stable motifs. The number of directed cycles that satisfy property 1, c, is typically much smaller than total the number of directed cycles, C. The limiting factor of the algorithm is c, which is typically smaller than the limiting factor of a brute-force search to attractor-finding, which is limited by the size of the network state space 2^N. For our test cases, the resulting number of cycles is $c_{TLGL} = 18,241$ and $c_{Th} = 63$ for the T-LGL leukemia network model and the helper T cell network model, respectively.

To illustrate how the number of directed cycles C varies for different networks, we consider the worst-case scenario and the typical behavior of C in terms of directed Erdős-Rényi graphs. Let $G = (V, E)$ be a directed network with $|V| = n$. The worst case scenario for the number of cycles is a fully connected network, for which C grows as $O((n-1)!)$. For a directed Erdős-Rényi graph $G(|V|, p = K/n)$, where K is the average degree of G, the probability to find a cycle of length L is $(K/n)^L$, while the number of such cycles is $n!/[2L(n-L)!]$. The average number cycles of length L is then

$$\overline{C}_L = \frac{1}{2L}\frac{n!}{(n-L)!}\left(\frac{K}{n}\right)^L, \tag{1}$$

which scales as $O(K^L)$ for small L and large n, and as $O(K^n/\sqrt{n})$ for large L and n. We note that even though the average number of large cycles for an Erdős-Rényi graph grows exponentially with n, this average is dominated by networks with large $L \sim n$, which have a low probability of appearing ($\sim (K/n)^n$) but have a large number of ways to have such cycles ($\sim (n-1)!/2$).

As the last paragraph illustrates, in some cases the number of directed cycles c of the expanded network is too large to enumerate. For these cases, we propose using a cutoff L_{max} in the modified Johnson's cycle algorithm for the maximum number of nodes in the stack from which cycles are obtained. The result of this cutoff is that only cycles with $L < L_{max}$ are output by the cycle algorithm. Unfortunately, this can result in overlooking cycles that are required to find all stable motifs, something which one should take into consideration. We should emphasize that even if a cutoff is used, we can still identify control interventions using a modified version of the stable motif control algorithm as long as the attractor of interest appears in the stable motif succession diagram obtained with the cutoff (see S3 Text section B.3).

4. Complexity of forming strongly connected components by the union of cycles

For each of the directed cycles in the expanded network that satisfy property 1, we check whether it satisfies property 2, and if it does, then the cycle is a stable motif. If it doesn't, we check that for each composite node $v^{(comp)}$ in the cycle, the complement of the inputs of $v^{(comp)}$ are not part of the cycle. The cycles that do not satisfy this condition are discarded, since they cannot form part of an SCC satisfying both property 1 and property 2. For the resulting set of cycles, we check whether for each composite node $v^{(comp)}$ the union of all cycles that have $v^{(comp)}$ as an element contains all inputs of $v^{(comp)}$. For the composite nodes for which this is not true, we discard all cycles containing such composite nodes, since SCCs containing such cycles cannot satisfy property 2. The result is a set of directed cycles in the expanded network that satisfy property 1 and that have the potential of being able to satisfy property 2. We call this set of directed cycles S_{simp}, and denote $c_{simp} = |S_{simp}|$ as the number of such cycles. For our test cases we have $c_{simp,TLGL} = 670$ and $c_{simp,Th} = 63$ for the T-LGL leukemia network model and the helper T cell network model, respectively.

At worse, all combinations of the directed cycles in S_{simp} can form an SCC that satisfies property 1 and 2, which makes the worst-case scenario $O(2^{C_{simp}})$. In practice, we find that the restriction of satisfying both property 1 and property 2 makes the number of possible combinations much smaller.

To illustrate this, let us describe in detail the process we use to find the stable motifs from S_{simp}. Given a composite node $v^{(comp)}$, we form all unions of cycles in S_{simp} which have the first input of $v^{(comp)}$ but do not have the second input of $v^{(comp)}$, and the cycles which have the second input of $v^{(comp)}$ but do not have the first input of $v^{(comp)}$, while discarding unions that do not satisfy property 1. The result is a group of SCCs that have both the first and second input of $v^{(comp)}$. We then form all unions of cycles/SCCs that have both the first and second input of $v^{(comp)}$ and the cycles which have the third input of $v^{(comp)}$ but do not have the first and second input of $v^{(comp)}$, while discarding the unions that do not satisfy property 1. We do this iteratively for all the inputs of $v^{(comp)}$. The result is $S_{simp,1}$, a set of SCCs that satisfy property 1 and property 2 for at least one composite node inside the SCC.

For each SCC in $S_{simp,1}$, we check whether it satisfies property 2 for all composite nodes; if it does, then this SCC is a stable motif candidate. If it does not, we check that for each composite node $v^{(comp)}$ in the SCC, the complement of the inputs of $v^{(comp)}$ are not part of the SCC. The SCCs that do not satisfy this condition are discarded from $S_{simp,1}$, since they cannot form part of an SCC satisfying both property 1 and property 2. For the resulting $S_{simp,1}$, we check whether for each composite node $v^{(comp)}$ the union of all SCCs that have $v^{(comp)}$ as an element contains all inputs of $v^{(comp)}$. For the composite nodes for which this is not true, we discard all SCCs in $S_{simp,1}$ containing such composite nodes, since SCCs containing such SCCs cannot satisfy property 2.

For the SCCs in $S_{simp,1}$, we repeat the same steps as before, that is, we form all unions of SCCs which share composite nodes, while discarding unions that do not satisfy property 1, until they satisfy property 2 for the composite node being considered. The result is a set of SCCs which we call $S_{simp,2}$, which we prune of SCCs that cannot satisfy both property 1 and property 2. We do this iteratively, until only SCCs that satisfy property 1 and property 2 for all composite nodes are left. Finally, we take all SCCs obtained during the process that satisfy property 1 and 2, and leave only SCCs which do not have other SCCs as a subset, which yields the smallest SCCs that satisfy property 1 and 2, i.e., the stable motifs.

As the worst case scenario illustrates, the number of combinations that can potentially become stable motifs can be too large to enumerate. For these cases, we propose using a cutoff L_{max} for the max number of nodes allowed in an SCC when taking the unions of SCCs/cycles. Unfortunately, like in the case of the cutoff for cycles, this can result in overlooking SCCs that are stable motifs or required to find all stable motifs, something which one should take into consideration. We should emphasize that even if a cutoff is used, we can still identify control interventions using a modified version of the stable motif control algorithm as long as the attractor of interest appears in the stable motif succession diagram obtained with the cutoff (see S3 Text section B.3).

B. The stable motif control method

A stable motif succession diagram can be represented as a directed graph $G_{diag} = (V_{diag}, E_{diag})$ together with a dictionary L. The nodes $V_{diag} = (v_{diag,1}, v_{diag,2}, \ldots, v_{diag,n})$ denote either stable motifs $\mathcal{M}_i$ (if the node has at least one outgoing edge) or attractors $\mathcal{A}_i$ (if the node has no outgoing edges). The dictionary L stores the type of object (stable motif or attractor) of each node in V_{diag} denotes. Each edge in E_{diag} connects a stable motif with the stable motifs or attractor that can be obtained from the reduced network associated to it; if network reduction leads to a simplified network with at least one stable motif, then the edges point from the stable motif being considered to the stable motifs of the simplified network, otherwise, the edge points towards an attractor. It should be noted that the same stable motif/attractor may be assigned to more than one node in V_{diag}.

For completeness, we reproduce the stable motif control algorithm (see Methods and S7 Text for more details):

- *Step 1*: Identify the sequences of stable motifs that lead to $\mathcal{A}$. These can be obtained from the stable motif succession diagram (see Fig. 2) by choosing the attractor of interest in the right-most part and selecting all of the attractor's predecessors in the succession diagram.

- *Step 2*: Shorten each sequence $\mathcal{S} \in Sequences$ by identifying the minimum number of motifs in $\mathcal{S}$ required for reaching $\mathcal{A}$ and removing the remaining motifs from the sequence. This minimum number of motifs can be identified from the stable motif succession diagram (Fig. 2); they are the motifs after which all consequent motif choices lead to the same attractor $\mathcal{A}$.

- *Step 3*: For each stable motif state $\mathcal{M} = (\sigma_{m_1} = b_{m_1}, \sigma_{m_2} = b_{m_2}, \ldots, \sigma_{m_l} = b_{m_l})$ corresponding to node v, find the subsets of stable motif's states $O = \{M_i\}, M_i \subseteq \mathcal{M}$ that, when fixed in the logical model, are enough to force the state of the whole motif into $\mathcal{M}$. At worst, there will only be one subset, which will equal the whole stable motif's state $\mathcal{M}$. If any of these subsets is fully contained in another subset, remove the larger of the subsets. In each stable motif sequence $\mathcal{S} = (\mathcal{M}_1, \ldots, \mathcal{M}_L)$, substitute every stable motif $\mathcal{M}_j$ with the subsets of the stable motif states obtained, that is, $\mathcal{S} = (O_1, \ldots, O_L)$.

- *Step 4*: For each sequence $\mathcal{S} = (O_1, \ldots, O_L)$ create a set of states $\mathcal{C}$ by choosing one of the subsets of stable motif's states M_{k_j} in each O_j and taking their union, that is, $\mathcal{C} = M_{k_1} \cup \cdots \cup M_{k_L}, M_{k_j} \in O_j$. The network control set for attractor $\mathcal{A}$ is the set of node states $C_{\mathcal{A}} = \{\mathcal{C}_i\}$ obtained from all possible combinations of subsets of stable motif's states M_{k_j}'s for every sequence $\mathcal{S}$. To avoid any redundancy, we additionally prune $C_{\mathcal{A}}$ of duplicates and remove each set of node states $\mathcal{C}_i$ which is a superset of any of the other sets of node states $\mathcal{C}_j$ (i.e. $\mathcal{C}_j \subset \mathcal{C}_i$).

In this section we restrict ourselves to the steps of the stable motif control method which we have found to be the most time consuming, namely, identifying the sequences of stable motifs that lead to an attractor (step 1) and finding the subsets of stable motif's states that fix the state of the whole stable motif (step 3). We also consider the identification of stable motif control interventions for the case when only part of the stable motif succession diagram is known.

1. *Complexity of identifying the sequences of stable motifs that lead to an attractor*

For a stable motif succession diagram G_{diag} with n_{sm} stable motifs, the worst case scenario in terms of the number sequences n_{seq} is when all permutations of the n_{sm} stable motifs form a sequence. In this case, the number of sequences n_{seq} is $O(n_{sm}!)$. In practice, the number of motif we find tends to be small and/or many of the motifs are not independent from each other, in which case the effective n_{sm} is much smaller. This allows us to manage even the worst case scenario as long as n_{sm} or the effective n_{sm} is smaller than ten. For example, $n_{sm,TLGL} = 7$ and $n_{seq,TLGL} = 144$ for the T-LGL leukemia network model, and $n_{sm,Th} = 17$ and $n_{seq,Th} = 697$ for the helper T cell network model. Note that the succession diagram of the T-LGL leukemia model shown in Fig. 4 does not include the part of the diagram associated with node P2, as motifs with P2 do not give rise to new motifs and do not influence the resulting attractor of any sequences in the succession diagram.

For the case of a network in which the number of sequences becomes computationally intractable, we suggest the following technique to simplify the stable motif succession diagram as the attractor-finding method is being applied. This technique takes into account which stable motifs are independent of each other and should significantly cut down the combinatorial explosion caused by allowing all possible permutations of independent motifs. Let $SM1 = \{\mathcal{M}_{SM1,1}, \mathcal{M}_{SM1,2}, \ldots, \mathcal{M}_{SM1,n_{SM1}}\}$ be all the stable motifs of a Boolean network, which could be the full Boolean model or one of the reduced networks obtained during the attractor-finding method (see S1 Text or S2 Text for details). Let $SM2 \subset SM1$ be the motifs $\mathcal{M} \in SM1$ such that

(a) $\mathcal{M}$ is still a stable motif after the first branching (i.e. if $\mathcal{M}, \mathcal{M}' \in SM2$ and $\mathcal{M} \neq \mathcal{M}'$, then $\mathcal{M} \to \mathcal{M}'$), and

(b) all successor motifs of $\mathcal{M}$ are still there after the second branching (i.e. if $\mathcal{M} \to \mathcal{M}'$, $\mathcal{M} \in SM2, \mathcal{M}' \notin SM2$, then $\mathcal{M} \to \mathcal{M}'' \to \mathcal{M}'$, $\forall \mathcal{M}'' \in SM2$),

then we can simplify the succession diagram by compressing the separate motifs of $SM2$ into a single group of motifs.

2. Complexity of finding the subsets of stable motif's states that fix the state of the whole stable motif

For a stable motif $\mathcal{M}$ composed of m nodes and their state, step 3 of the stable motif control algorithm has the objective to find the subsets of stable motif's states $M \subseteq \mathcal{M}$ such that, when fixed, are enough to force the state of the whole motif into $\mathcal{M}$, restricted to the condition that the resulting subsets are not fully contained in another subset of the result. At worst, there is only one such subset, namely, the whole stable motif state $\mathcal{M}$.

In algorithm 5 in S7 Text we give a procedure to find this subsets of motif's states $M \subseteq \mathcal{M}$. In principle, almost every possible combination of the m node states in $\mathcal{M}$ could be obtained during the process we proposed, making the worst-case scenario have a complexity of $O(2^m)$. For our test cases, even the worst case scenario wasn't a problem, since the maximum motif size was seven for both the T-LGL leukemia network model and the helper T cell network model.

For the cases where the number of node states m in M is too large, we propose using a modified version of algorithm 5 where a cutoff $L_{max,1}$ is introduced for the maximum subset size considered (i.e., changing "**for** *subsetSize* $\leftarrow 1$ to length of list $\mathcal{M} - 1$" to "**for** *subsetSize* $\leftarrow 1$ to $L_{max,1}$"). Additionally, we propose searching for subsets while starting from a subset size of $m - 1$ and ending at a subset size of $L_{max,2} > L_{max,1}$ (i.e., repeat the instructions in the loop "**for** *subsetSize* $\leftarrow 1$ to length of list $\mathcal{M} - 1$" just after the it ends, but starting the loop with "**for** *subsetSize* $\leftarrow \mathcal{M} - 1$ to length of list $L_{max,2}$"). Unlike the other cases where a cutoff is introduced, the cutoffs $L_{max,1}$ and $L_{max,2}$ do not require a modification to the control method to guarantee the method's effectiveness (see S3 Text section B.3), their only effect is that the control interventions obtained with these cutoffs may involve more nodes or have more redundancy than the interventions obtained without the cutoffs.

3. Identifying stable motif control interventions with partial knowledge of the stable motif succession diagram

In S2 Text sections A.3 and A.4 we considered the scenario in which the number of cycles and/or SCCs becomes computationally intractable, which prompted us to introduce a cutoff in the maximum length of cycles allowed and/or the maximum SCC size allowed. In both of these cases, the introduction of this cutoff may cause the overlooking of some stable motifs. If this is the case, the result of the attractor-finding method is not the full stable motif succession diagram, but only a part of it. Here we look at how to identify stable motif control interventions when only a part of the stable motif succession diagram is known.

Our results in S2 Text section B show that a sequence of stable motifs in the stable motif succession diagram uniquely determines an attractor, and that fixing the subset of the states determining a motif specified in step 3 has the same effect as fixing all node states in the stable motif (see S2 Text for more details). This implies that step 1, 3 and 4 are still applicable even if only a part of the stable motif succession diagram is known. On the other hand, step 2 requires knowing whether all consequent motif choices after a certain motif lead to the same attractor and, thus, needs the knowledge of all the stable motif decision diagram. The result is that the modified stable motif control algorithm is the same as the original algorithm except for step 2, which is skipped.
