# Supplementary material for: Cell Fate Reprogramming by Control of Intracellular Network Dynamics
Source: PLoS Comput Biol. 2015 Apr 7;11(4):e1004193. doi: 10.1371/journal.pcbi.1004193 (PMC4388852; doi:10.1371/journal.pcbi.1004193)
Supplement: S4 Text — (PDF) [file pcbi.1004193.s004.pdf]

Supporting Information

S4 TEXT. LOGICAL RULES AND CLASSIFICATION OF ATTRACTORS IN THE T-LGL LEUKEMIA NETWORK MODEL

A. Logical rules of the T-LGL leukemia network model

These rules dictate the dynamics of the T-LGL leukemia survival signaling network depicted in Fig. 3. For simplicity, the node states are represented by the node names. The Boolean rules were constructed based on experimental results of the corresponding intracellular components in normal and leukemic cytotoxic T cells, in such a way that that the model reproduces the known experimental behavior. The interested reader is referred to [28] for the detailed explanation of the rules. For transparency of interpretation we slightly diverge from [28] by not allowing a single transient activation of the Apoptosis node to drive cell death. For this reason these rules do not include the "AND NOT Apoptosis" clause on each node, and the auto-activation of Apoptosis that [28] has. This slight change, also used in [46], does not change the results qualitatively.

f_{CTLA4} = TCR
f_{TCR} = Stimuli AND NOT CTLA4
f_{PDGFR} = S1P OR PDGF
f_{FYN} = TCR OR IL2RB
$f_{Cytoskeleton\ signaling}$ = FYN
f_{LCK} = CD45 OR ((TCR OR IL2RB) AND NOT ZAP70)
f_{ZAP70} = LCK AND NOT FYN
f_{GRB2} = IL2RB OR ZAP70
f_{PLCG1} = GRB2 OR PDGFR
f_{RAS} = (GRB2 OR PLCG1) AND NOT GAP
f_{GAP} = (RAS OR (PDGFR AND GAP)) AND NOT (IL15 OR IL2)
f_{MEK} = RAS
f_{ERK} = MEK AND PI3K
f_{PI3K} = PDGFR OR RAS
f_{NFKB} = (TPL2 OR PI3K) OR (FLIP AND TRADD AND IAP)
f_{NFAT} = PI3K
f_{RANTES} = NFKB
f_{IL2} = (NFKB OR STAT3 OR NFAT) AND NOT TBET
f_{IL2RBT} = ERK AND TBET
f_{IL2RB} = IL2RBT AND (IL2 OR IL15)
f_{IL2RAT} = IL2 AND (STAT3 OR NFKB)
f_{IL2RA} = IL2 AND IL2RAT AND NOT IL2RA
f_{JAK} = (IL2RA OR IL2RB OR RANTES OR IFNG) AND NOT (SOCS OR CD45)
f_{SOCS} = JAK AND NOT (IL2 OR IL15)
f_{STAT3} = JAK
f_{P27} = STAT3
$f_{Proliferation}$ = STAT3 AND NOT P27
f_{TBET} = JAK OR TBET
f_{CREB} = ERK AND IFNG
f_{IFNGT} = TBET OR STAT3 OR NFAT
f_{IFNG} = ((IL2 OR IL15 OR Stimuli) AND IFNGT) AND NOT (SMAD OR P2)
f_{P2} = (IFNG OR P2) AND NOT Stimuli2
f_{GZMB} = (CREB AND IFNG) OR TBET
f_{TPL2} = TAX OR (PI3K AND TNF)
f_{TNF} = NFKB
f_{TRADD} = TNF AND NOT (IAP OR A20)
f_{FasL} = STAT3 OR NFKB OR NFAT OR ERK
f_{FasT} = NFKB
f_{Fas} = FasT AND FasL AND NOT sFas
f_{sFas} = FasT AND S1P AND NOT Apoptosis

$f_{Ceramide}$ = Fas AND NOT S1P
f_{DISC} = FasT AND ((Fas AND IL2) OR Ceramide OR (Fas AND NOT FLIP))
$f_{Caspase}$ = (((TRADD OR GZMB) AND BID) AND NOT IAP) OR DISC
f_{FLIP} = (NFKB OR (CREB AND IFNG)) AND NOT DISC
f_{A20} = NFKB
f_{BID} = (Caspase OR GZMB) AND NOT (BclxL OR MCL1)
f_{IAP} = NFKB AND NOT BID
f_{BclxL} = (NFKB OR STAT3) AND NOT (BID OR GZMB OR DISC)
f_{MCL1} = (IL2RB AND STAT3 AND NFKB AND PI3K) AND NOT DISC
$f_{Apoptosis}$ = Caspase
f_{GPCR} = S1P
f_{SMAD} = GPCR
f_{SPHK1} = PDGFR
f_{S1P} = SPHK1 AND NOT Ceramide

B. Classification of attractors in the T-LGL leukemia network model

To classify the attractors in the T-LGL leukemia network we use the state of the node Apoptosis; ON for apoptosis and OFF for T-LGL leukemia. This is the same criterion used by Saadatpour et al. [46]. This criterion groups several attractors into the T-LGL leukemia attractor class and several others into the apoptosis attractor class. Thus, stable motif blocking is not successful by default.

The attractor states classified as T-LGL leukemia attractors differ from one another in the activity of some nodes (e.g. IL2RB, IL2RBT, IL2, and IL2RA), but most of them are characterized by the inhibition of Fas-induced apoptosis pathway elements (e.g. Caspase=OFF, DISC=OFF, TRADD=OFF, Fas=OFF, FasL=ON, FasT=ON and Ceramide=OFF), and the activation of transcription factors (e.g. NFKB=ON, TPL2=ON and IFNGT=ON), receptors (e.g. PDGFR=ON and GPCR=ON), or kinases (e.g. S1P=ON, SPHK1=ON, and PI3K=ON). The attractor states classified as Apoptosis attractors are characterized by the activation of Caspase (Caspase=ON) by Fas-induced apoptosis pathway elements such as DISC=ON, Ceramide=ON, Fas=ON, IAP=OFF, GZMB=ON, and BID=ON.
