# Supplementary material for: Cell Fate Reprogramming by Control of Intracellular Network Dynamics
Source: PLoS Comput Biol. 2015 Apr 7;11(4):e1004193. doi: 10.1371/journal.pcbi.1004193 (PMC4388852; doi:10.1371/journal.pcbi.1004193)
Supplement: S5 Text — (PDF) [file pcbi.1004193.s005.pdf]

Supporting Information

S5 TEXT. LOGICAL RULES, CLASSIFICATION OF ATTRACTORS, AND ANALYSIS OF THE STABLE MOTIF DECISION DIAGRAM IN THE HELPER T CELL DIFFERENTIATION NETWORK MODEL

A. Logical rules of the helper T cell differentiation network model developed by Naldi et al. [48]

For our study we use one of environmental conditions studied by Naldi et al. [48], namely, the presence of antigen presenting cells (APC=ON), external TGFβ (TGFB_e=ON), external IL2 (IL2_e=ON), and the absence of other external cytokines (IFNB_e=OFF, IFNG_e=OFF, IL4_e=OFF, IL6_e=OFF, IL10_e=OFF, IL12_e=OFF, IL15_e=OFF, IL21_e =OFF, IL23_e=OFF, and IL27_e=OFF). The helper T cell differentiation network with only the considered input signals is shown in Fig. 5. For simplicity, the node states are represented by the node names. For the nodes that have three states (0, 1 and 2), we created an extra node (denoted by Nodename_2) that represents the third state (2) and adapted the rules accordingly. The interested reader is referred to the work of Naldi et al. [48] for a detailed justification of the logical rules.

f_{CD28} = APC
f_{IFNBR} = IFNB_e
f_{IFNGR} = IFNGR1 AND IFNGR2 AND (IFNG OR IFNG_e)
f_{IL2R} = CGC AND IL2RB AND (NOT IL2RA) AND (IL2 OR IL2_e)
f_{IL2R_2} =CGC AND IL2RB AND IL2RA AND (IL2 OR IL2_e)
f_{IL4R} = CGC AND NOT IL4RA AND (IL4 OR IL4_e)
f_{IL4R_2} =CGC AND IL4RA AND (IL4 OR IL4_e)
f_{IL6R}= GP130 AND IL6RA AND IL6_e
f_{IL10R} = IL10RA AND IL10RB AND (IL10 OR IL10_e)
f_{IL12R} = IL12RB1 AND IL12RB2 AND IL12_e
f_{IL15R} = CGC AND IL15RA AND IL2RB AND IL15_e
f_{IL21R} = GP130 AND CGC AND (IL21 OR IL21_e)
f_{IL23R} = GP130 AND (IL23 OR IL23_e) AND STAT3 AND RORGT
f_{IL27R} = GP130 AND IL27RA AND IL27_e
f_{TCR} = APC
f_{TGFBR} = TGFB OR TGFB_e
$f_{IL12RB1}$ = IRF1
f_{IL4RA} = STAT5_2
f_{IL2RA} =(SMAD3 OR FOXP3 OR (STAT5 OR STAT5_2) OR NFKB) AND NFAT
f_{IFNG} = Proliferation AND NOT FOXP3 AND NOT STAT3 AND NFAT AND ((TBET AND RUNX3) OR STAT4)
f_{IL2} = ((NFAT AND NOT FOXP3) OR NFKB) AND (NOT (STAT5 OR STAT5_2) OR NOT STAT6) AND (NOT NFKB OR NOT TBET)
f_{IL4} = NFAT AND Proliferation AND GATA3 AND NOT FOXP3 AND NOT ((TBET AND RUNX3) OR IRF1)
f_{IL10} = (GATA3 OR STAT3) AND NFAT AND Proliferation
f_{IL21} = NFAT AND Proliferation AND STAT3
f_{IL23} = NFAT AND Proliferation AND STAT3
f_{TGFB} = NFAT AND Proliferation AND FOXP3
f_{TBET} = (TBET OR STAT1) AND NOT GATA3
f_{GATA3} = (GATA3 OR STAT6) AND NOT TBET
f_{FOXP3} = (STAT5 OR STAT5_2) AND NFAT AND (FOXP3 OR (SMAD3 AND NOT STAT1 AND NOT (STAT3 AND RORGT)))
f_{NFAT} = CD28 AND TCR
f_{STAT1} = IFNBR OR IFNGR OR IL27R
f_{STAT3} = IL6R OR IL10R OR IL21R OR IL23R OR IL27R
f_{STAT4} = IL12R AND NOT GATA3
f_{STAT5} = (IL4R OR IL2R OR IL15R) AND NOT IL2R_2 AND NOT IL4R_2
f_{STAT5_2} = IL2R_2 OR IL4R_2
f_{STAT6} = IL4R OR IL4R_2
f_{SMAD3} = TGFBR
f_{IRF1} = STAT1

f_{RUNX3} = TBET
$f_{Proliferation}$ = STAT5_2 OR Proliferation
f_{NFKB} = NOT IKB AND NOT FOXP3
f_{IKB} = NOT TCR
f_{RORGT} = (TGFBR AND STAT3) OR (RORGT AND (TGFBR OR STAT3))
f_{IL17} = NFAT AND Proliferation AND RORGT AND NOT FOXP3 AND NFKB AND STAT3 AND NOT ((STAT5 OR STAT5_2) OR STAT1 OR STAT6)
f_{IFNGR1} = ON
f_{IFNGR2} = ON
f_{GP130} = ON
f_{IL6RA} = ON
f_{CGC} = ON
$f_{IL12RB2}$ = ON
f_{IL10RB} = ON
f_{IL10RA} = ON
f_{IL15RA} = ON
f_{IL2RB} = ON
f_{IL27RA} = ON

B. Classification of attractors in the helper T cell differentiation network model

To classify the attractors in the helper T cell differentiation network we use the same criteria used by Naldi et al. [48]: TBET=ON for Th1; TBET=OFF and GATA3=ON for Th2; TBET=OFF, GATA3=OFF, and FOXP3=ON for Treg; and TBET=OFF, GATA3=OFF, FOXP3=OFF, and RORGT=ON for Th17. These criteria group several attractors into each attractor class. Consequently, stable motif blocking is not successful by default.

As explained in the work by Naldi et al., the attractor states in an attractor class share the expression of many nodes apart from their master regulator (TBET, GATA3, FOXP3, and/or RORGT), but can also be very similar to attractor states of other attractor classes. This gives rise to hybrid cells types co-expressing markers of more than one canonical cell type which are classified according to the above criteria. These criteria are used because they are consistent with the differentiation of naive helper T cells into the different helper T cell subtypes under the appropriate environmental signals.

C. Analysis of the stable motif decision diagram for helper T cell differentiation network

Using the attractor-finding method on the helper T cell differentiation network we obtain 17 stable motifs and a stable motif decision diagram composed of 697 sequences. Despite the size of the decision diagram, a closer look at it suggests a simple explanation: the stable motifs associated with each attractor regulate the characteristic transcription factor of each helper T cell subtype. To check this, we look at the minimal subsets of stable motifs that are sufficient for a sequence to lead to a single differentiated helper T cell subtype. Each subset is minimal because removing any stable motif allows sequences with that subset to lead to more than one helper T cell subtype.

The minimal subsets of stable motifs associated to each helper T cell subtype are shown in Fig. 6. Most of these subsets contain a motif with the defining transcription factor of each helper T cell subtype: TBET=ON for Th1, GATA3=ON for Th2, RORGT=ON for Th17, and FOXP3=ON for Treg. The motif subsets that do not contain a subtype's characteristic transcription factor can be shown to depend on the stabilization of a motif that includes this transcription factor (e.g., the motif IFNGR=IFNG=STAT=ON, FOXP3=OFF in Fig. 6(a) requires a stable motif with TBET=ON to stabilize) or that causes the differential expression of this transcription factor (e.g., the stable motif subsets in Fig. 6(c) are sufficient to cause RORGT=ON and a Th17 subtype when taken together with the motifs they depend on, despite RORGT not being part of any of the motifs). This shows that the minimal subsets of stable motifs in the decision diagram regulate the characteristic transcription factor of each helper T cell subtype.
