# Supplementary material for: Cell Fate Reprogramming by Control of Intracellular Network Dynamics
Source: PLoS Comput Biol. 2015 Apr 7;11(4):e1004193. doi: 10.1371/journal.pcbi.1004193 (PMC4388852; doi:10.1371/journal.pcbi.1004193)
Supplement: S6 Text — (PDF) [file pcbi.1004193.s006.pdf]

Supporting Information

S6 TEXT. TRANSLATING THE LOGICAL NETWORK MODELS INTO ORDINARY DIFFERENTIAL EQUATION MODELS, AND INTERVENTION TARGET VALIDATION FOR THE ORDINARY DIFFERENTIAL EQUATION MODELS

A. Translating the logical network models into ordinary differential equation models

We use the method described by Wittmann et al. [49] and its MATLAB implementation [58] to translate the studied Boolean network models into ordinary differential equation (ODE) models. In the ODE models, the node state variables $\widetilde{\sigma}_i$ take continuous values in the range $[0,1]$ and their time evolution is given by

$$\frac{d\widetilde{\sigma}_i}{dt} = \frac{1}{\tau_i}\left[\widetilde{f}_i(\widetilde{\sigma}_{i_1}, \widetilde{\sigma}_{i_2}, \ldots, \widetilde{\sigma}_{i_{k_i}}) - \widetilde{\sigma}_i\right],\ i = 1, 2, \ldots, N, \tag{1}$$

where $\widetilde{\sigma}_{i_1}, \ldots, \widetilde{\sigma}_{i_{k_i}}$ are the state variables of the inputs of node i, $\widetilde{f}_i$ is a smooth Hill-type function parameterized by a set of Hill coefficient $\{n_{i_1}, \ldots, n_{i_{k_i}}\}$ and threshold parameters $\{\theta_{i_1}, \ldots, \theta_{i_{k_i}}\}$ for each input, and τ_i is a time-scale parameter. The function $\widetilde{f}_i$, a so-called normalized Hillcube [49, 58], is a continuous analogue of the Boolean function f_i and is such that it matches f_i whenever all its input variables are either 0 or 1.

This type of logical-to-ODE model conversion is far from trivial, as evidenced by the great number of studies in this topic [32, 34, S13] and the fact that some aspects of this type of conversion are still not fully understood [S14, S15, S16, S17]. For example, even though the fixed point attractors of the Boolean model are guaranteed to be preserved in the ODE model, this is not necessarily the case for complex attractors. The ODE model may also have attractors that have no Boolean equivalent. Some of these not fully understood aspects, namely, the appearance of attractors of the ODE system with no Boolean equivalent and the mapping of Boolean complex attractors to the ODE system, play a role on our test cases; for example, the T-LGL leukemia network model has an ODE attractor with no Boolean equivalent, and the T-LGL leukemia attractor is a complex attractor (albeit with only two oscillating nodes). These types of ODE attractors can still be classified using the criteria explained in section B of S4 Text and section B of S5 Text. Despite this, we find that the control interventions are remarkably effective in the ODE version of the model, though not always 100% effective.

B. Intervention target validation for the ordinary differential equation models

To validate an intervention target in the ordinary differential equation model, we fix the node states in the continuous equivalent of the states in the logical model interventions (1 for ON and 0 for OFF), choose a random uniformly chosen initial condition in the continuous interval $[0,1]^N$, and evolve the system using Eq. 1. The system of ordinary differential equations is solved using MATLAB's ode45 function, based on the Runge-Kutta method by Dormand and Prince [S18]. The error tolerances in the ode45 function are chosen between 10^{-2} and 10^{-3}, while the rest of the function's parameters are left at their default value.

Each initial condition is evolved for a long time to ensure that the system reaches an attractor. We find that, for our test cases, evolving from $t = 0$ to $t = 10$ is enough to ensure reaching an attractor from any initial condition considered with stable motif control intervention or without an intervention; to be safe, we choose to evolve from $t = 0$ to $t = 15$ in all cases, and for twice the time for the few initial conditions in which the state at $t = 15$ did not match any previously found attractor. We repeat this for a large number of initial conditions (25,000, unless otherwise specified) and calculate the probability of reaching each attractor from a uniformly chosen initial condition. For the case when the intervention is not permanent (i.e. it is transient), we evolve the system with the intervention for a long period of time, remove the intervention, and then evolve the system for another long period of time. For our test cases, we find that using 10 units of time for each evolution stage (with and then without prescribed node states) is enough to preserve the first three digits of the estimated probabilities p_{Attr} of reaching the attractor of interest, consistent with what is expected from the standard deviation of the estimated probability p_{Attr}. To be safe, we choose to evolve for 15 units of time for each evolution stage.

The number of initial conditions we use is chosen to give three significant figures in the estimated probabilities p_{Attr}. For our test cases, we find that 25,000 initial conditions are enough to estimate the probabilities p_{Attr} of reaching the attractor of interest with an error (standard deviation of the estimated probability p_{Attr}) of $6{\cdot}10^{-3}\,[p_{Attr}(1 - p_{Attr})]^{1/2}$. Equivalently, if p_{Attr} is expressed as a percentage (which we denote as $\%p_{Attr}$ for clarity), the error in it is estimated

as $6 \cdot 10^{-3} \left[\%p_{Attr}(100\% - \%p_{Attr})\right]^{1/2}\%$ (e.g. 0.06% for a $\%p_{Attr}$ of 1%, and 0.3% for a $\%p_{Attr}$ of 50%). The number of time steps we use is enough to show no changes in p_{Attr} beyond what is expected from the standard deviation of the estimated probability p_{Attr}, and is also found to be enough for the initial conditions to reach the attractors when no interventions are applied.

For the results in S3 Table and S4 Table we use the default parameters: a Hill coefficient of $n = 3$, a threshold parameter of $\theta = 0.5$, and a time-scale parameter $\tau = 1$ for all nodes. For S5 Table we use the Hill coefficient specified in the table ($n = 1, 1.5, 2,$ or 2.5), a threshold parameter of $\theta = 0.5$, and a time-scale parameter $\tau = 1$ for all nodes. For S7 Table we fix the intervention at the values specified (0.9, 0.8, 0.7 or 0.6 if the Boolean intervention was 1, or 0.1, 0.2, 0.3, or 0.4 if the Boolean intervention was 0), and use a Hill coefficient of $n = 3$, a threshold parameter of $\theta = 0.5$, and a time-scale parameter $\tau = 1$ for all nodes. Finally, for the results in S6 Table we use 1,000 initial conditions and 40 different networks in which the Hill coefficients take the values specified in the table ($n = 1, 1.5, 2, 2.5,$ or 3), and the thresholds θ_i and time-scale parameters τ_i for each node are chosen uniformly from the interval $[0, 1]$.
