# Supplementary material for: Cell Fate Reprogramming by Control of Intracellular Network Dynamics
Source: PLoS Comput Biol. 2015 Apr 7;11(4):e1004193. doi: 10.1371/journal.pcbi.1004193 (PMC4388852; doi:10.1371/journal.pcbi.1004193)
Supplement: S7 Text — (PDF) [file pcbi.1004193.s007.pdf]

Supporting Information

S7 TEXT. PSEUDOCODE FOR THE STABLE MOTIF CONTROL ALGORITHM AND THE STABLE MOTIF BLOCKING ALGORITHM

For the pseudocodes we assume that one starts with a target attractor $\mathcal{A}$, the logical functions $F = (f_1, f_2, \ldots, f_N)$ for the logical network model of interest, and the stable motif succession diagram for the logical network model of interest (see Fig. 2). A stable motif succession diagram can be represented as a directed graph $G_{diag} = (V_{diag}, E_{diag})$ together with a dictionary L. The nodes $V_{diag} = (v_{diag,1}, v_{diag,2}, \ldots, v_{diag,n})$ denote either stable motifs $\mathcal{M}_i$ (if the node has at least one outgoing edge) or attractors $\mathcal{A}_i$ (if the node has no outgoing edges). The dictionary L stores the type of object (stable motif or attractor) each node in V_{diag} denotes. Each edge in E_{diag} connects a stable motif with the stable motifs or attractors that can be obtained from the reduced network associated to it; if network reduction leads to a simplified network with at least one stable motif, then the edges points from the stable motif being considered to the stable motifs of the simplified network, otherwise, the edges point towards an attractor. It should be noted that stable motifs/attractors may be assigned to more than one node in V_{diag}. For example, in Fig. 2 there are three nodes that denote the motif $\{A = 0\}$, and two nodes that denote the attractor $\mathcal{A}_2$.

A. Pseudocode for the stable motif control algorithm

Step 1: Identify the sequences of stable motifs that lead to $\mathcal{A}$. These can be obtained from the stable motif succession diagram (see Fig. 2) by choosing the attractor of interest in the right-most part and selecting all of the attractor's predecessors in the succession diagram. The stable motif diagram is represented by the directed graph $G_{diag} = (V_{diag}, E_{diag})$ together with the list L.

Algorithm 1: GetSequences($G, L, \mathcal{A}$)

```
comment: Sequences, SequencesLeft, and NewSequences are sets.
         S is a sequence (ordered list).
Sequences ← empty set
SequencesLeft ← empty set
for each v ∈ sink nodes of G
  do { comment: L(v) gives the motif or attractor denoted by v.
       if L(v) equals A
         then { S ← empty sequence
                add v to the beginning of S
                add S to SequencesLeft
repeat
  { NewSequences ← empty set
    for each S ∈ SequencesLeft
      do { v ← first item of S
           if v has input nodes
             then { for each v' ∈ input nodes of v
                      do { S' ← copy S
                           add v' to the beginning of S'
                           add S' to NewSequences
             else add S to Sequences
           remove S from SequencesLeft
    for each S' ∈ NewSequences
      do add S' to SequencesLeft
until NewSequences is empty
return (Sequences)
```

Step 2: Shorten each sequence $\mathcal{S} \in Sequences$ by identifying the minimum number of motifs in $\mathcal{S}$ required for reaching $\mathcal{A}$ and removing the remaining motifs from the sequence. This minimum number of motifs can be identified from the stable motif succession diagram (Fig. 2); they are the motifs after which all consequent motif choices lead to the same attractor $\mathcal{A}$.

Algorithm 2: SHORTENSEQUENCES1($G, L, \mathcal{A}, Sequences$)

```
comment: ShortenedSequences1 is a set.
         S' is a sequence (ordered list).
         pathFound is a Boolean variable
ShortenedSequences1 ← empty set
for each S ∈ Sequences
  do {
    S' ← copy S
    for each v ∈ S in reverse order
      do {
        pathFound ← false
        for v' ∈ sink nodes of G
          do {
            comment: L(v') gives the motif or attractor denoted by v'.
            if L(v') is not A
              then {
                if there exists a directed path from v to v'
                  then {
                    pathFound ← true
                    exit for loop
                  }
              }
          }
        if pathFound
          then exit for loop
          else remove v from S'
      }
    if ShortenedSequences1 does not contain S'
      then add S' to ShortenedSequences1
  }
return (ShortenedSequences1)
```

Step 3: For each stable motif state $\mathcal{M} = (\sigma_{m_1} = b_{m_1}, \sigma_{m_2} = b_{m_2}, \ldots, \sigma_{m_l} = b_{m_l})$ corresponding to node v, find the subsets of stable motif's states $O = \{M_i\}, M_i \subseteq \mathcal{M}$ that, when fixed in the logical model, are enough to force the state of the whole motif into $\mathcal{M}$. At worst, there will only be one subset, which will equal the whole stable motif state $\mathcal{M}$. If any of these subsets is fully contained in another subset, remove the larger of the subsets. In each stable motif sequence $\mathcal{S} = (\mathcal{M}_1, \ldots, \mathcal{M}_L)$, substitute every stable motif $\mathcal{M}_j$ with the subsets of the stable motif states obtained, that is, $\mathcal{S} = (O_1, \ldots, O_L)$.

Algorithm 3: SequencesWithMotifControlSets($ShortenedSequences1, SequenceDictionary, F, L$)

```
comment: F = (f_1, f_2, ..., f_N) contains the Boolean functions of the logical model.
         ShortenedSequences2 is a set.
         O and Subsequence are sequences (ordered lists).
ShortenedSequences2 ← empty set
for each S ∈ ShortenedSequences1
  do {
    comment: index is an integer. It stores the index of the first element of S'
             that will be visited in the for loop below.
             S' and S'' are sequences (ordered lists).
             F' is a sequence (ordered list) of Boolean functions.
    index ← 0
    S' ← sequence assigned to S in SequenceDictionary
    S'' ← empty sequence
    F' ← copy F
    for each v ∈ S
      do {
        comment: S' has more motifs than S,
                 we need the extra motifs to find the reduced network from which the motif
                 L(v) was obtained. These extra motifs are stored in Subsequence
        Subsequence ← empty sequence
        for i ← index to length of list S' − 1
          do {
            v' ← get element of S' in position i
            if v' equals v
              then { index ← i + 1
                     exit for loop
            add v' to the end of Subsequence
          }
        comment: DownstreamEffect(L(v'), F') is described in Algorithm 4.
                 DownstreamEffect(L(v'), F') evaluates the states of motif L(v') into F'.
                 If any f ∈ F' becomes a constant Boolean function after the evaluation,
                 it evaluates the resulting Boolean state of the node corresponding to
                 f in every F'. This is done iteratively until no new constant Boolean
                 functions are found, at which point the resulting F' is returned.
        for each v' ∈ Subsequence
          do F' ← DownstreamEffect(L(v'), F')
        comment: MotifControlSet(L(v), F') is described in Algorithm 5
                 MotifControlSet(L(v), F') finds the subsets of stable motif's states
                 of L(v) that, when fixed, are enough to force the state of the whole motif
                 into L(v).
        O ← MotifControlSet(L(v), F')
        add O to end of S''
        F' ← DownstreamEffect(L(v), F')
      }
    add S'' to ShortenedSequences2
  }
return (ShortenedSequences2)
```

Algorithm 4: DOWNSTREAMEFFECT($\mathcal{M}, F'$)

```
comment: DOWNSTREAMEFFECT(M, F') evaluates the states of motif M into F'.
         If any f ∈ F' becomes a constant Boolean function after the evaluation,
         it evaluates the resulting Boolean state of the node corresponding to
         f in every F'. This is done iteratively until no new constant Boolean
         functions are found, at which point the resulting F' is returned.
         M' and M'' are sets containing nodes in the logical model together with a
         Boolean variable with their state.
         F'' is a sequence (ordered lists) of Boolean functions.
M' ← empty set; M'' ← copy M; F'' ← copy F'
repeat
  for each f ∈ F''
    do if f is not a constant Boolean function
         then f ← f with the states in M' evaluated on it
              if f is a constant Boolean function
                then comment: σ is a node in the logical model together
                              with a Boolean variable with its state.
                     σ ← node in the logical model whose function is f and
                         the value of f as its state.
                     add σ to M''
  M' ← copy M''
  M'' ← empty set
until M' is empty
return (F'')
```

Algorithm 5: MOTIFCONTROLSET($\mathcal{M}, F'$)

```
comment: MOTIFCONTROLSET(M, F') finds the subsets of stable motif's states of M that,
         when fixed, are enough to force the state of the whole motif into M.
         F' and F'' are sequences (ordered lists) of Boolean functions.
         F' are the logical functions of the nodes in the model whose states are specified in M.
         O is a sequence (ordered list).
         isMotifControlSet and validSubset are Boolean variables.
O ← empty sequence
for subsetSize ← 1 to length of list M − 1
  do for each M ∈ subsets of size subsetSize in M
       do validSubset ← true
          for each M' ∈ O
            do if M' is a subset of M
                 then validSubset ← false
                      exit for loop
          if not validSubset
            then exit for loop
          comment: DOWNSTREAMEFFECT(M, F') is described in Algorithm 4.
          F'' ← DOWNSTREAMEFFECT(M, F')
          isMotifControlSet ← true
          for each f ∈ F''
            do if f is not a constant Boolean function
                 then isMotifControlSet ← false
                      exit for loop
          if isMotifControlSet
            then add M to O
if O is empty
  then add M to O
return (O)
```

Step 4: For each sequence $\mathcal{S} = (O_1, \ldots, O_L)$ create a set of states $\mathcal{C}$ by choosing one of the subsets of stable motif states M_{k_j} in each O_j and taking their union, that is, $\mathcal{C} = M_{k_1} \cup \cdots \cup M_{k_L}, M_{k_j} \in O_j$. The network control set for attractor $\mathcal{A}$ is the set of states $C_{\mathcal{A}} = \{\mathcal{C}_i\}$ obtained from all possible combinations of M_{k_j}'s for every sequence $\mathcal{S}$. To avoid any redundancy, we additionally prune $C_{\mathcal{A}}$ of duplicates and remove the states $\mathcal{C}_i$ which are supersets of any of the other states $\mathcal{C}_j$ (i.e. $\mathcal{C}_j \subset \mathcal{C}_i$).

Algorithm 6: StableMotifControlSets(*ShortenedSequences2*)

```
comment: ControlSets, ControlSet, and M are sets
         O is a sequence (ordered list).
         L and index are integers.
         countArray and countArrayMax are arrays of integers.
ControlSets ← empty set
for each S ∈ ShortenedSequences2
  do
    L ← length of list S
    comment: countArray and countArrayMax keep track of the combinations of motifs
             in S that we have tried and that we have left.
    countArray ← array of integers of length L
    countArrayMax ← array of integers of length L
    for i ← 0 to L − 1
      do
        O ← get element of S in position i
        countArrayMax[i] ← length of list O
        countArray[i] ← 0
    repeat
      ControlSet ← empty set
      for i ← 0 to L − 1
        do
          O ← get element of S in position i
          M ← get element of O in position countArray[i]
          for each σ ∈ M
            do add σ to ControlSet
      add ControlSets to ControlSets
      comment: index gets increased whenever countArray[index] reaches its
               max value, countArrayMax[index].
      index ← 0
      repeat
        comment: increasedIndex breaks the repeat loop.
        increasedIndex ← false
        countArray[index] ← countArray[index] + 1
        if countArray[index] equals countArrayMax[index]
          then
            countArray[index] ← 0
            index ← index + 1
            increasedIndex ← true
        if index equals L
          then exit repeat loop
      until not increasedIndex
    until index equals L
return (ControlSets)
```

Algorithm 7: PruneControlSets(*ControlSets*)

```
comment: PrunedControlSets is a set
PrunedControlSets ← copy ControlSets
for each ControlSet ∈ ControlSets
  do { for each ControlSet′ ∈ ControlSets
         do { if ControlSet′ is not ControlSet
                then { if ControlSet′ is a subset of ControlSet
                         then { remove ControlSet from PrunedControlSets
                                exit for loop
return (PrunedControlSets)
```

B. Pseudocode for the stable motif blocking algorithm

Step 1: Identify the sequences of stable motifs that lead to $\mathcal{A}$. This step is the same as the first step in the stable motif control algorithm (Algorithm 1), and can be obtained from the stable motif succession diagram (Fig. 2).

Step 2: Take each stable motif's state $\mathcal{M}_i$ in the sequences obtained in the previous step (*Sequences*). Create a new set $\mathbf{M}_{\mathcal{A}}$ with all of these stable motif states, $\mathbf{M}_{\mathcal{A}} = \{\mathcal{M}_i\}$.

Algorithm 8: MotifStates(*Sequences*, L)

```
comment: M_A and M are sets.
M_A ← empty set
for each S ∈ Sequences
  do { for each v ∈ S s.t. v is not a sink node
         do { comment: M stores the states of the motif L(v).
              M ← L(v)
              add M to M_A
return (M_A)
```

Step 3: Take each node state $\sigma_j \subset \mathcal{M}_i$ of the stable motif's states $\mathcal{M}_i$ in $\mathbf{M}_{\mathcal{A}}$. Create a new set $\mathcal{B}_{\mathcal{A}}$ with the negation of each node state, $\mathcal{B}_{\mathcal{A}} = \{\overline{\sigma}_j\}$. The node states in $\mathcal{B}_{\mathcal{A}}$ and any combination of them are identified as potential interventions to block attractor $\mathcal{A}$.

Algorithm 9: StableMotifBlocking($\mathbf{M}_{\mathcal{A}}$)

```
comment: B_A is a set.
B_A ← empty set
for each σ ∈ M_A
  do { comment: σ′ is a node in the logical model together with a Boolean variable with its state.
       σ′ ← reverse node state of σ
       add σ′ to B_A
return (B_A)
```
