# Supplementary material for: Cell Fate Reprogramming by Control of Intracellular Network Dynamics
Source: PLoS Comput Biol. 2015 Apr 7;11(4):e1004193. doi: 10.1371/journal.pcbi.1004193 (PMC4388852; doi:10.1371/journal.pcbi.1004193)
Supplement: S8 Text — (PDF) [file pcbi.1004193.s008.pdf]

Supporting Information

S8 TEXT. LIST OF REFERENCES THAT APPEAR IN THE SUPPORTING INFORMATION FILES

[S1] Wang RS and Albert R (2011) Elementary signaling modes predict the essentiality of signal transduction network components. BMC Systems Biology 5, 44.
[S2] Saadatpour A, Albert R, and Reluga T (2013) A reduction method for Boolean network models proven to conserve attractors. SIAM J. Appl. Dyn. Syst. 12(4), 19972011.
[S3] Naldi A, Monteiro PT, Müssel C, Kestler HA, Thieffry D, et al. (2014) Cooperative development of logical modelling standards and tools with CoLoMoTo. bioRxiv doi: 10.1101/010504.
[S4] Aldana-Gonzalez M, Coppersmith S, and Kadanoff LP (2003) Boolean Dynamics with Random Couplings. In Perspectives and Problems in Nonlinear Science. A celebratory volume in honor of Lawrence Sirovich, Springer Applied Mathematical Sciences Series. Ehud Kaplan, Jerrold E. Marsden, and Katepalli R. Sreenivasan Eds., 23-89.
[S5] McCluskey EJ (1956) Minimization of Boolean Functions. Bell System Technical Journal 35 (6), 14171444.
[S6] Quine WV (1952) The Problem of Simplifying Truth Functions. The American Mathematical Monthly 59 (8), 521531
[S7] Quine WV (1955) A Way to Simplify Truth Functions. The American Mathematical Monthly 62 (9), 627631.
[S8] Coudert O (1994) Two-level logic minimization: an overview. Integration, the VLSI Journal 17 (2), 97140.
[S9] Chandra AK and Markowsky G (1978) On the number of prime implicants. Discrete Mathematics 24 (1), 711.
[S10] McMullen C and Shearer J (1986) Prime Implicants, Minimum Covers, and the Complexity of Logic Simplification. IEEE Transactions on Computers archive 35 (8), 761-762.
[S11] Strzemecki T (1992) Polynomial-time algorithms for generation of prime implicants. Journal of Complexity 8 (1), 376.
[S12] Johnson DB (1975) Finding All the Elementary Circuits of a Directed Graph. SIAM J. Comput. 4 (1), 77-84.
[S13] De Jong H, Gouzé JL, Hernandez C, Page M, Sari T, and Geiselmann J (2004) Qualitative simulation of genetic regulatory networks using piecewise-linear models. Bull. Math. Biol. 66 (2), 301-340.
[S14] Snoussi EH (1989) Qualitative dynamics of piecewise-linear differential equations: a discrete mapping approach. Dynamics and Stability of Systems 4 (3-4), 189-207.
[S15] Casey R, de Jong H, and Gouzé JL (2006) Piecewise-linear models of genetic regulatory networks: equilibria and their stability. J. Math. Biol. 52 (1), 27-56.
[S16] Farcot E and Gouzé JL (2009) Periodic solutions of piecewise affine gene network models with non uniform decay rates: the case of a negative feedback loop. Acta Biotheor. 57, 429-455.
[S17] Chaves M and Preto M (2013) Hierarchy of models: From qualitative to quantitative analysis of circadian rhythms in cyanobacteria. Chaos 23 (2), 025113.
[S18] Dormand JR and Prince PJ (1980) A family of embedded Runge-Kutta formulae. J. Comp. Appl. Math. 6, 1926.
