# Supplementary material for: Cell Fate Reprogramming by Control of Intracellular Network Dynamics
Source: PLoS Comput Biol. 2015 Apr 7;11(4):e1004193. doi: 10.1371/journal.pcbi.1004193 (PMC4388852; doi:10.1371/journal.pcbi.1004193)
Supplement: S1 Fig — Read from left to right, the figure shows the logical network in Fig 1, the stable motifs of this logical network, the simplified networks obtained from tracing the downstream effect of each of the original logical network’s stable motifs, and the stable motifs obtained from these simplified networks. Nodes are colored based on their respective node state: gray for 0, black for 1, and white for nodes whose state is not yet determined. Each large arrow has an associated stable motif sharing the arrow’s color. These large arrows stand for the use of a network reduction technique on the network they start from by tracing the downstream effect of their associated stable motifs on this network. (PDF) [file pcbi.1004193.s009.pdf]

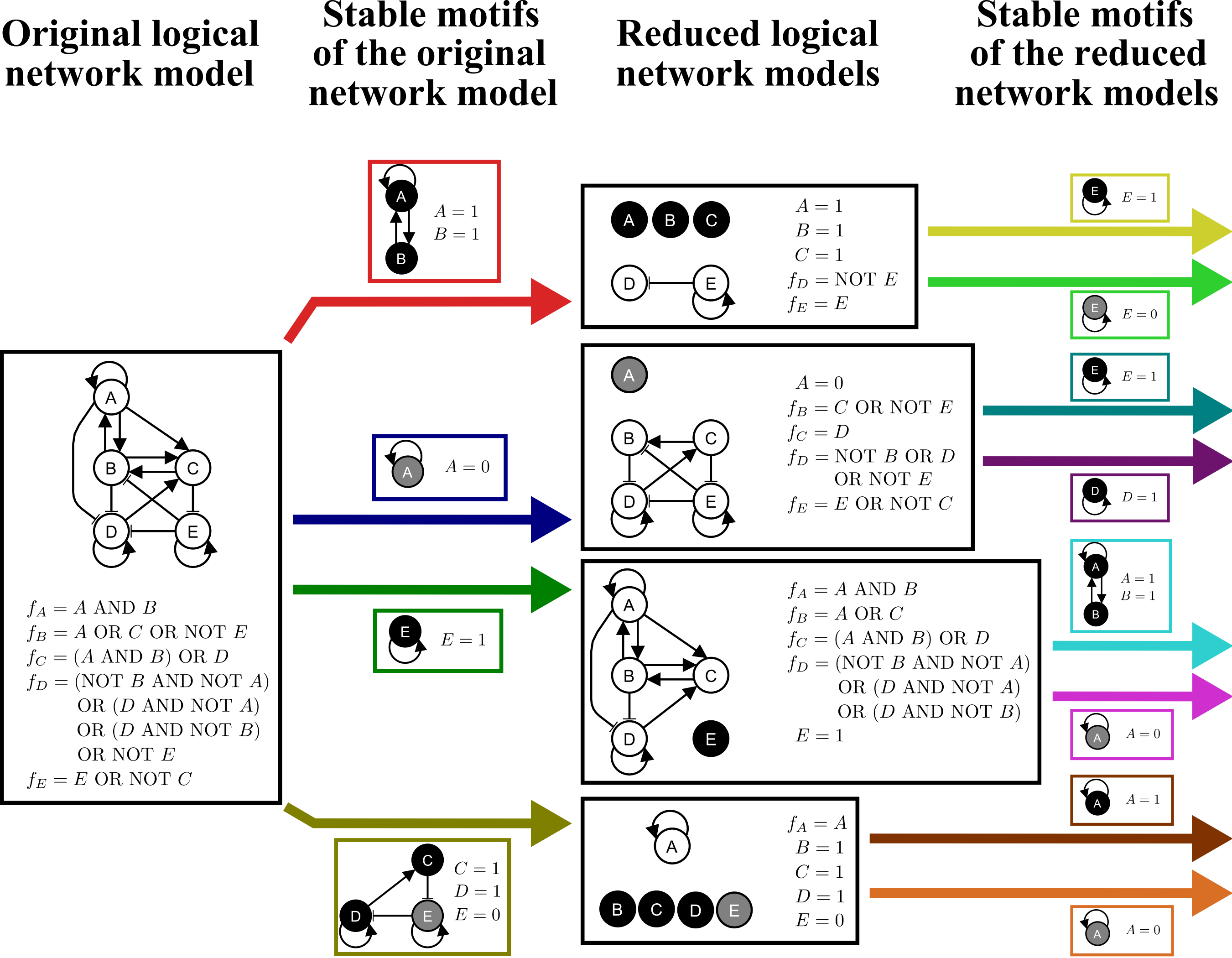
Original logical network model
Stable motifs of the original network model
Reduced logical network models
Stable motifs of the reduced network models
$f_A = A$ AND B
$f_B = A$ OR C OR NOT E
$f_C = (A$ AND $B)$ OR D
$f_D = ($NOT B AND NOT $A)$
OR $(D$ AND NOT $A)$
OR $(D$ AND NOT $B)$
OR NOT E
$f_E = E$ OR NOT C
$A = 1$
$B = 1$
$A = 0$
$E = 1$
$C = 1$
$D = 1$
$E = 0$
$A = 1$
$B = 1$
$C = 1$
$f_D =$ NOT E
$f_E = E$
$A = 0$
$f_B = C$ OR NOT E
$f_C = D$
$f_D =$ NOT B OR D
OR NOT E
$f_E = E$ OR NOT C
$f_A = A$ AND B
$f_B = A$ OR C
$f_C = (A$ AND $B)$ OR D
$f_D = ($NOT B AND NOT $A)$
OR $(D$ AND NOT $A)$
OR $(D$ AND NOT $B)$
$E = 1$
$f_A = A$
$B = 1$
$C = 1$
$D = 1$
$E = 0$
$E = 1$
$E = 0$
$E = 1$
$D = 1$
$A = 1$
$B = 1$
$A = 0$
$A = 1$
$A = 0$
