# Supplementary material for: Cell Fate Reprogramming by Control of Intracellular Network Dynamics
Source: PLoS Comput Biol. 2015 Apr 7;11(4):e1004193. doi: 10.1371/journal.pcbi.1004193 (PMC4388852; doi:10.1371/journal.pcbi.1004193)
Supplement: S2 Fig — The logical function of each example node is shown above its expanded network representation. Nodes are colored white if they denote normal nodes or complementary node (complementary nodes have a bar above their name, while normal nodes do not), and colored black if they denote composite nodes. For more details see S1 Text and S2 Text. (a) Expanded network representation for normal node C, complementary node C¯, and their inputs. (b) Expanded network representation for normal node B, complementary node B¯, and their inputs. (PDF) [file pcbi.1004193.s010.pdf]

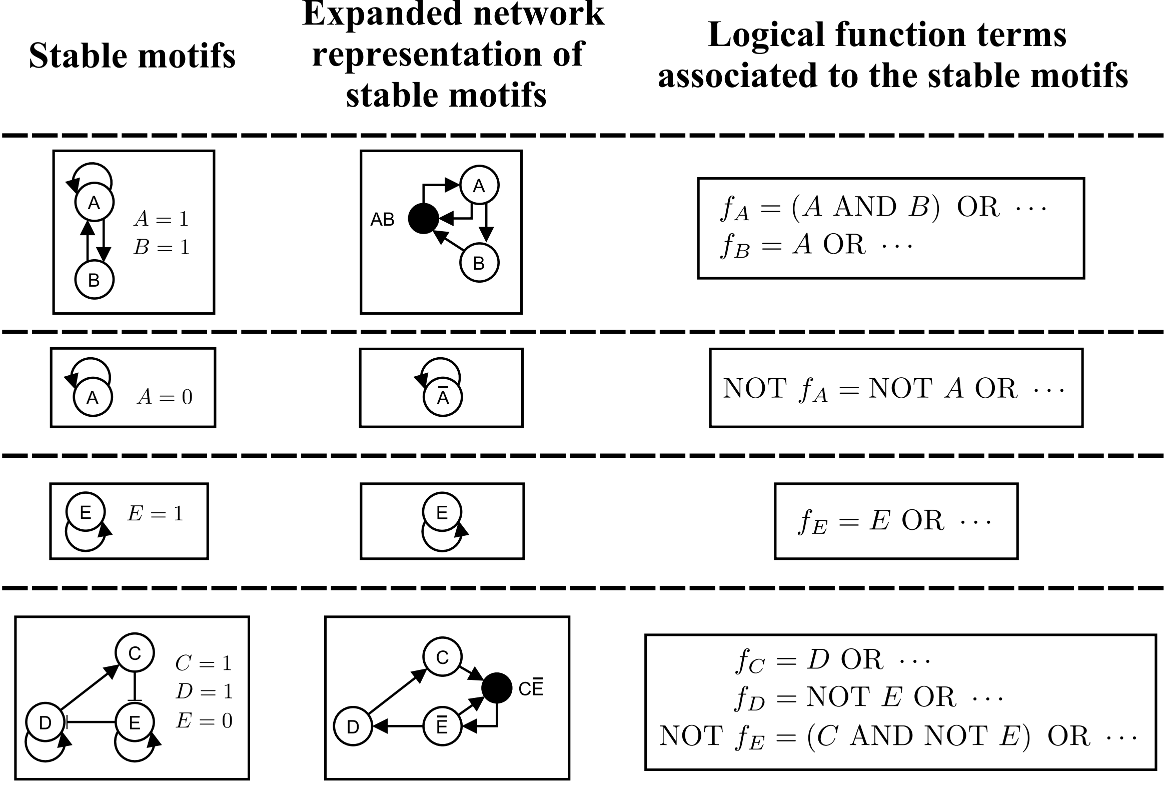
Stable motifs
Expanded network representation of stable motifs
Logical function terms associated to the stable motifs
A
B
A = 1
B = 1
AB
A
B
f_A = (A AND B) OR ⋯
f_B = A OR ⋯
A
A = 0
Ā
NOT f_A = NOT A OR ⋯
E
E = 1
E
f_E = E OR ⋯
C
D
E
C = 1
D = 1
E = 0
C
CĒ
D
Ē
f_C = D OR ⋯
f_D = NOT E OR ⋯
NOT f_E = (C AND NOT E) OR ⋯
