# Supplementary figures and images for: Cell Fate Reprogramming by Control of Intracellular Network Dynamics
Source: PLoS Comput Biol. 2015 Apr 7;11(4):e1004193. doi: 10.1371/journal.pcbi.1004193 (PMC4388852; doi:10.1371/journal.pcbi.1004193)

(a)

$$f_A = (\text{NOT } A \text{ AND NOT } B) \\ \text{OR } (A \text{ AND } B)$$

$$f_B = (\text{NOT } A \text{ AND NOT } B) \\ \text{OR } (A \text{ AND } B)$$

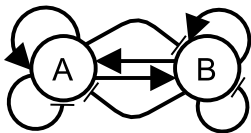

(b)

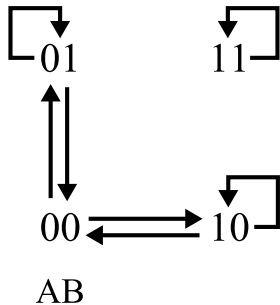

(c)

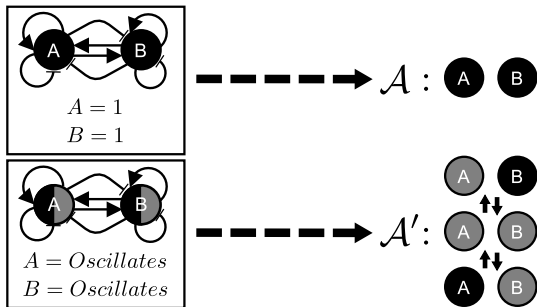

Supplement: S3 Fig — The figure shows (a) a two node Boolean network whose logical functions are given by an XOR function, (b) the network’s state transition graph, i.e., all combinations of network states and the allowed transitions between them under the general asynchronous updating scheme, and (c) the network’s stable motif succession diagram. This Boolean network is the simplest example (up to a relabeling of node states) of so-called unstable oscillations. Unstable oscillations refer to a subset of nodes whose node states oscillate in an attractor while their node states are fixed in a different attractor, even though both attractors are the same except for the state of this subset of nodes. In the example Boolean network shown in this figure, we have the states of nodes A and B oscillate between three network states in attractor ′ = {(A = 1,B = 0), (A = 0,B = 0), (A = 0,B = 1)}, while they are fixed in attractor = {A = 1,B = 1}. Unstable oscillations are treated with special care when using our attractor-finding method, since ignoring them can lead to missing attractors displaying this behavior; for more details see S1 Text and S2 Text. (PDF) [file pcbi.1004193.s011.pdf]
