# Supplementary material for: Cell Fate Reprogramming by Control of Intracellular Network Dynamics
Source: PLoS Comput Biol. 2015 Apr 7;11(4):e1004193. doi: 10.1371/journal.pcbi.1004193 (PMC4388852; doi:10.1371/journal.pcbi.1004193)
Supplement: S4 Fig — The figure shows (a) a three node Boolean network that displays incomplete oscillations, (b) the sub-state-space of nodes A and B in the network’s state transition graph (i.e., all combinations of network states and the allowed transitions between them) under the general asynchronous updating scheme, and (c) the network’s stable motif succession diagram. Incomplete oscillations refer to a subset of nodes whose node states oscillate in an attractor but do not visit all possible states of their sub-state-space in the attractor. In the example Boolean network shown in this figure, we have the states of nodes A and B oscillate between three subnetwork states {(A = 1,B = 0), (A = 0,B = 0), (A = 0,B = 1)} in the attractors and ′. Incomplete oscillations are treated with special care when using our attractor-finding method, since ignoring them can lead to missing attractors displaying this behavior; for more details see S1 Text and S2 Text. (PDF) [file pcbi.1004193.s012.pdf]

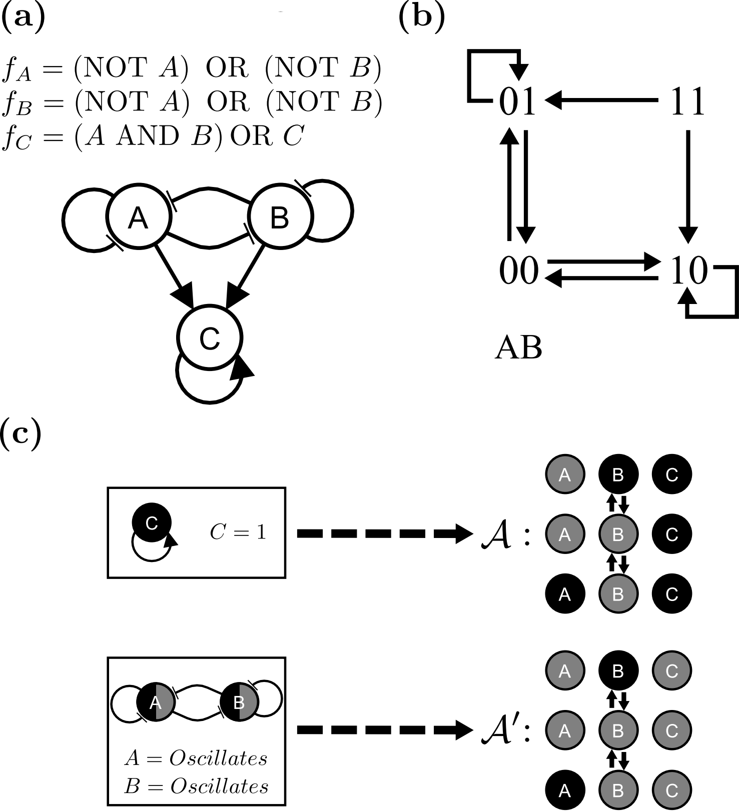
(a)
$f_A = (\text{NOT } A) \text{ OR } (\text{NOT } B)$
$f_B = (\text{NOT } A) \text{ OR } (\text{NOT } B)$
$f_C = (A \text{ AND } B) \text{ OR } C$
A
B
C
(b)
01
11
00
10
AB
(c)
C
$C = 1$
$\mathcal{A}$:
A
B
C
A
B
A
$A = Oscillates$
$B = Oscillates$
$\mathcal{A}'$:
