# Supplementary material for: Cell Fate Reprogramming by Control of Intracellular Network Dynamics
Source: PLoS Comput Biol. 2015 Apr 7;11(4):e1004193. doi: 10.1371/journal.pcbi.1004193 (PMC4388852; doi:10.1371/journal.pcbi.1004193)

(a)

$$f_C = (A \text{ AND } B) \text{ OR } D$$

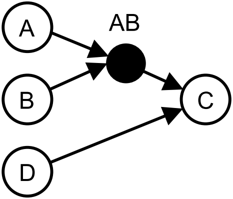

$$f_{\overline{C}} = (\text{NOT } D \text{ AND NOT } A) \text{ OR } (\text{NOT } D \text{ AND NOT } B)$$

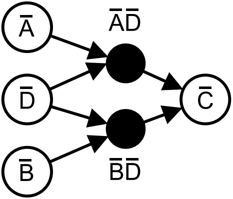

(b)

$$f_B = A \text{ OR } C \text{ OR NOT } E$$

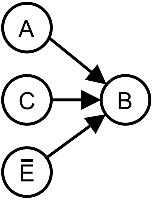

$$f_{\overline{B}} = \text{NOT } A \text{ AND NOT } C \text{ AND } E$$

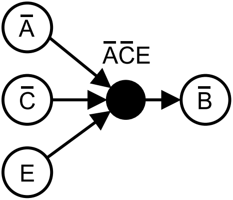

Supplement: S5 Fig — Read from left to right, the figure shows the stable motifs of the logical network in Fig 1, the expanded network representation of the stable motifs (from which stable motifs are formally defined), and the terms of the logical function associated to each stable motif. For more details on the expanded network representation see S1 Text and S2 Text. (PDF) [file pcbi.1004193.s013.pdf]
