# Supplementary material for: Cell Fate Reprogramming by Control of Intracellular Network Dynamics
Source: PLoS Comput Biol. 2015 Apr 7;11(4):e1004193. doi: 10.1371/journal.pcbi.1004193 (PMC4388852; doi:10.1371/journal.pcbi.1004193)
Supplement: S2 Table — The relative attractor % change is defined as (attractor %−normal attractor %)/(normal attractor %), where the normal attractor % is the percentage of initial conditions that go to the attractor of interest when no intervention is applied. The normal attractor percentages are 48.6%, 47.5%, 1.3%, and 2.6% for the Th1, Th2, Th17, and Treg helper T cell subtypes, respectively. Interventions marked with † appear in more than one control strategy or target attractor in Table 2. The percentages are significant in the digits shown and have an estimated absolute error (standard deviation of the mean) of 3⋅10−3[%p Attr(100%−%p Attr)]1/2 %, where %p Attr is the percentage shown (e.g. 0.03% for a %p Attr of 1%, and 0.15% for a %p Attr of 50%). (PDF) [file pcbi.1004193.s015.pdf]

S2 Table. Validation of the intervention targets in Table 2 and single interventions from control sets with more than one node in Table 2 for the helper T cell network. The relative attractor % change is defined as (attractor % − normal attractor %)/(normal attractor %), where the normal attractor % is the percentage of initial conditions that go to the attractor of interest when no intervention is applied. The normal attractor percentages are 48.6 %, 47.5 %, 1.3 %, and 2.6 % for the Th1, Th2, Th17, and Treg helper T cell subtypes, respectively. Interventions marked with † appear in more than one control strategy or target attractor in Table 2. The percentages are significant in the digits shown and have an estimated absolute error (standard deviation of the mean) of $3 \cdot 10^{-3}[\%p_{Attr}(100\% - \%p_{Attr})]^{1/2}$ %, where $\%p_{Attr}$ is the percentage shown (e.g. 0.03% for a $\%p_{Attr}$ of 1%, and 0.15% for a $\%p_{Attr}$ of 50%).

Intervention	Successful?	Long-term?	Attractor % (permanent intervention)	Relative attractor % change (permanent intervention)	Attractor % (nonpermanent intervention)	Relative attractor % change (nonpermanent intervention)
Th1 stable motif control interventions (C_{Th1})						
{TBET=ON}	Yes	Yes	100.0	106	100.0	106
Th2 stable motif control interventions (C_{Th2})						
{GATA3=ON}	Yes	Yes	100.0	111	100.0	111
Th17 stable motif control interventions (C_{Th17})						
{GATA3=OFF, FOXP3=OFF, TBET=OFF, STAT3=ON}	Yes	Yes	100.0	7357	100.0	7357
{GATA3=OFF, FOXP3=OFF, TBET=OFF, IL10=ON}	Yes	Yes	100.0	7357	100.0	7357
{GATA3=OFF, FOXP3=OFF, TBET=OFF, IL10R=ON}	Yes	Yes	100.0	7357	100.0	7357
{GATA3=OFF, FOXP3=OFF, TBET=OFF, IL21=ON}	Yes	Yes	100.0	7357	100.0	7357
{GATA3=OFF, FOXP3=OFF, TBET=OFF, IL21R=ON}	Yes	Yes	100.0	7357	100.0	7357
{GATA3=OFF, FOXP3=OFF, TBET=OFF, IL23R=ON, RORGT=ON}	Yes	Yes	100.0	7357	100.0	7357
Treg stable motif control interventions (C_{Treg})						
{GATA3=OFF, FOXP3=ON, TBET=OFF}	Yes	Yes	100.0	3706	100.0	3706
{GATA3=OFF, TBET=OFF, STAT3=OFF}	Yes	Yes	100.0	3706	100.0	3706
{GATA3=OFF, TBET=OFF, IL23R=OFF, IL10R=OFF, IL21R=OFF}	Yes	Yes	100.0	3706	100.0	3706
{GATA3=OFF, TBET=OFF, IL23R=OFF, IL10=OFF, IL21R=OFF}	Yes	Yes	100.0	3706	100.0	3706
{GATA3=OFF, TBET=OFF, IL23R=OFF, IL10R=OFF, IL21=OFF}	Yes	Yes	100.0	3706	100.0	3706

Intervention	Successful?	Long-term?	Attractor % (permanent intervention)	Relative attractor % change (permanent intervention)	Attractor % (nonpermanent intervention)	Relative attractor % change (nonpermanent intervention)
Treg stable motif control interventions (C_{Treg}) (continuation)						
{GATA3=OFF, TBET=OFF, IL23R=OFF, IL10=OFF, IL21=OFF}	Yes	Yes	100.0	3706	100.0	3706
Th1 stable motif blocking interventions (B_{Th1})						
{GATA3=ON}†	Yes	Yes	0.0	-100	0.0	-100
{IL4=ON}†	No	No	48.2	-1	48.1	-1
{IL4R 2=ON}†	No	No	47.2	-3	47.4	-2
{STAT6=ON}†	No	No	45.3	-7	45.0	-7
{STAT1=OFF}	Yes	Yes	37.2	-23	37.5	-23
{IFNG=OFF}	No	No	48.2	-1	48.0	-1
{IFNGR=OFF}	No	No	47.0	-3	46.8	-4
{IL23=OFF}†	No	No	48.7	0	48.9	1
{IL10=ON}†	No	No	48.6	0	48.8	1
{IL10=OFF}†	No	No	48.9	1	48.7	0
{IL10R=ON}†	No	No	48.8	1	48.6	0
{IL10R=OFF}†	No	No	48.6	0	48.9	1
{IL21=ON}†	No	No	48.0	-1	48.8	0
{IL21=OFF}†	No	No	48.7	0	48.4	0
{IL21R=ON}†	No	No	48.8	0	48.6	0
{IL21R=OFF}†	No	No	48.6	0	48.7	0
{STAT3=ON}†	No	No	48.8	1	48.8	0
{IL23R=ON}†	No	No	48.6	0	48.6	0
{IL23R=OFF}†	No	No	48.7	0	49.1	1
{RORGT=ON}†	No	No	48.7	0	48.9	1
{RORGT=OFF}†	No	No	48.7	0	48.6	0
{FOXP3=ON}†	No	No	48.6	0	48.4	0
{FOXP3=OFF}†	No	No	48.7	0	48.7	0
Th2 stable motif blocking interventions (B_{Th2})						
{TBET=ON}†	Yes	Yes	0.0	-100	0.0	-100
{GATA3=OFF}	Yes	Yes	0.0	-100	0.0	-100
{STAT1=ON}†	No	No	44.7	-6	44.7	-6
{IFNG=ON}†	No	No	47.3	0	47.0	-1
{IFNGR=ON}†	No	No	46.6	-2	46.6	-2
{IL23=OFF}†	No	No	47.5	0	47.3	0
{IL23R=OFF}†	No	No	47.5	0	47.1	-1
{STAT3=OFF}†	No	No	47.3	0	47.3	0
{IL10=OFF}†	No	No	47.3	0	47.5	0
{IL10R=OFF}†	No	No	47.6	0	47.3	0
{RORGT=ON}†	No	No	47.5	0	47.3	0
{FOXP3=ON}†	No	No	47.5	0	47.7	0
{FOXP3=OFF}†	No	No	47.6	0	47.5	0

Intervention	Successful?	Long-term?	Attractor % (permanent intervention)	Relative attractor % change (permanent intervention)	Attractor % (nonpermanent intervention)	Relative attractor % change (nonpermanent intervention)
Th17 stable motif blocking interventions (B_{Th17})						
{GATA3=ON}†	Yes	Yes	0.0	-100	0.0	-100
{TBET=ON}†	Yes	Yes	0.0	-100	0.0	-100
{IL4=ON}†	Yes	Yes	0.0	-100	0.0	-100
{IL4R_2=ON}†	Yes	Yes	0.0	-100	0.0	-100
{STAT6=ON}†	Yes	Yes	0.0	-100	0.0	-100
{STAT1=ON}†	Yes	Yes	0.0	-100	0.0	-100
{IFNG=ON}†	Yes	Yes	0.0	-100	0.0	-100
{IFNGR=ON}†	Yes	Yes	0.0	-100	0.0	-100
{STAT3=OFF}†	Yes	Yes	0.0	-100	0.0	-100
{FOXP3=ON}†	Yes	Yes	0.0	-100	0.0	-100
{RORGT=OFF}†	Yes	Yes	0.0	-100	0.0	-100
{IL21=OFF}†	Yes	Yes	1.1	-20	1.1	-20
{IL21R=OFF}†	Yes	Yes	1.0	-28	1.0	-23
{IL23=OFF}†	Partial	Partial	1.2	-11	1.2	-12
{IL23R=OFF}†	Yes	Yes	1.1	-19	1.1	-19
{IL10=OFF}†	Yes	Yes	1.1	-20	1.1	-20
{IL10R=OFF}†	Yes	Yes	1.0	-28	1.0	-29
Treg stable motif blocking interventions (B_{Treg})						
{GATA3=ON}†	Yes	Yes	0.0	-100	0.0	-100
{TBET=ON}†	Yes	Yes	0.0	-100	0.0	-100
{IL4=ON}†	Yes	Yes	0.0	-100	0.0	-100
{IL4R_2=ON}†	Yes	Yes	0.0	-100	0.0	-100
{STAT6=ON}†	Yes	Yes	0.0	-100	0.0	-100
{STAT1=ON}†	Yes	Yes	0.0	-100	0.0	-100
{IFNG=ON}†	Yes	Yes	0.0	-100	0.0	-100
{IFNGR=ON}†	Yes	Yes	0.0	-100	0.0	-100
{STAT3=ON}†	Yes	Yes	0.6	-76	0.6	-76
{STAT3=OFF}†	No	No	3.7	41	3.7	42
{FOXP3=OFF}†	Yes	Yes	0.0	-100	2.0	-23
{RORGT=ON}†	No	No	2.4	-10	2.4	-9
{RORGT=OFF}†	No	No	3.9	48	3.9	50
{IL21=ON}†	Yes	Yes	1.1	-60	1.0	-61
{IL21=OFF}†	No	No	2.7	2	2.7	3
{IL21R=ON}†	Yes	Yes	0.8	-70	0.8	-71
{IL21R=OFF}†	No	No	2.9	12	2.8	7
{IL23=OFF}†	No	No	2.6	-2	2.7	2
{IL23R=ON}†	Yes	Yes	0.8	-70	0.8	-69
{IL23R=OFF}†	No	No	2.8	6	2.7	3
{IL10=ON}†	Yes	Yes	1.1	-60	1.0	-60
{IL10=OFF}†	No	No	2.7	4	2.7	4
{IL10R=ON}†	Yes	Yes	0.7	-72	0.8	-70
{IL10R=OFF}†	No	No	2.9	10	2.8	8

Intervention	Successful?	Long-term?	Attractor % (permanent intervention)	Relative attractor % change (permanent intervention)	Attractor % (nonpermanent intervention)	Relative attractor % change (nonpermanent intervention)
Single interventions of Th17 stable motif control sets						
{GATA3=OFF}	Yes	Yes	6.3	369	6.2	359
{FOXP3=OFF}	Partial	Partial	1.7	25	1.8	31
{TBET=OFF}	Yes	Yes	7.5	461	7.6	468
{STAT3=ON}	Yes	Yes	3.3	146	3.2	142
{IL10=ON}	Yes	Yes	2.8	110	2.9	114
{IL10R=ON}	Yes	Yes	3.1	132	3.1	130
{IL21=ON}	Yes	Yes	3.0	120	2.8	107
{IL21R=ON}	Yes	Yes	3.1	127	3.1	133
{IL23R=ON}	Yes	Yes	3.1	134	3.1	130
{RORGT=ON}	No	No	1.5	9	1.4	6
Single interventions of Treg stable motif control sets						
{GATA3=OFF}	Yes	Yes	12.0	358	11.9	354
{FOXP3=ON}	Partial	Partial	3.9	49	3.9	49
{TBET=OFF}	Yes	Yes	13.5	415	13.7	423
{STAT3=OFF}	Partial	Partial	3.7	41	3.7	42
{IL21=OFF}	No	No	2.3	-13	2.7	3
{IL21R=OFF}	No	No	2.6	-2	2.8	7
{IL23R=OFF}	No	No	2.8	6	2.7	3
