# Supplementary material for: Cell Fate Reprogramming by Control of Intracellular Network Dynamics
Source: PLoS Comput Biol. 2015 Apr 7;11(4):e1004193. doi: 10.1371/journal.pcbi.1004193 (PMC4388852; doi:10.1371/journal.pcbi.1004193)
Supplement: S3 Table — The relative apoptosis % change is defined as (Apoptosis %−Normal apoptosis %)/(Normal apoptosis %), where Normal apoptosis % = 54.7% is the percentage of initial conditions that go to apoptosis when no intervention is applied. Interventions marked with † appear in more than one control strategy or target attractor in Table 1. The percentages are significant in the digits shown and have an estimated absolute error (standard deviation of the mean) of 6⋅10−3[%p Attr(100%−%p Attr)]1/2 %, where %p Attr is the percentage shown (e.g. 0.06% for a %p Attr of 1%, and 0.3% for a %p Attr of 50%). (PDF) [file pcbi.1004193.s016.pdf]

S3 Table. Validation of the intervention targets in Table 1 for the T-LGL leukemia differential equation network model and single interventions from control sets with more than one node in Table 1 for the T-LGL leukemia differential equation network model. The relative apoptosis % change is defined as (Apoptosis % − Normal apoptosis %)/(Normal apoptosis %), where Normal apoptosis % = 54.7 % is the percentage of initial conditions that go to apoptosis when no intervention is applied. Interventions marked with † appear in more than one control strategy or target attractor in Table 1. The percentages are significant in the digits shown and have an estimated absolute error (standard deviation of the mean) of $6 \cdot 10^{-3}[\%p_{Attr}(100\% - \%p_{Attr})]^{1/2}$ %, where $\%p_{Attr}$ is the percentage shown (e.g. 0.06% for a $\%p_{Attr}$ of 1%, and 0.3% for a $\%p_{Attr}$ of 50%).

Intervention	Successful?	Long-term?	Apoptosis % (permanent intervention)	Relative apoptosis % change (permanent intervention)	Apoptosis % (nonpermanent intervention)	Relative apoptosis % change (nonpermanent intervention)
T-LGL stable motif control interventions (C_{TLGL})						
{S1P=ON}†	Yes	Yes	0.0	-100	0.0	-100
{Ceramide=OFF, SPHK1=ON}	Yes	Yes	0.0	-100	0.0	-100
{Ceramide=OFF, PDGFR=ON}	Yes	Yes	0.0	-100	0.0	-100
Apoptosis stable motif control interventions ($C_{Apoptosis}$)						
{S1P=OFF}†	Yes	Yes	99.9	83	99.9	83
{SPHK1=OFF}†	Yes	Yes	99.9	83	99.9	83
{PDGFR=OFF}†	Yes	Yes	99.8	83	99.8	83
{TBET=ON, Ceramide=ON, RAS=ON}	Yes	Yes	100.0	83	100.0	83
{TBET=ON, Ceramide=ON, GRB2=ON}	Yes	Yes	100.0	83	100.0	83
{TBET=ON, Ceramide=ON, IL2RB=ON}	Yes	Yes	100.0	83	100.0	83
{TBET=ON, Ceramide=ON, IL2RBT=ON}	Yes	Yes	100.0	83	100.0	83
{TBET=ON, Ceramide=ON, ERK=ON}	Yes	Yes	100.0	83	100.0	83
{TBET=ON, Ceramide=ON, MEK=ON, PI3K=ON}	Yes	Yes	100.0	83	100.0	83
T-LGL stable motif blocking interventions (B_{TLGL})						
{Ceramide=ON}	Yes	Yes	99.9	83	99.9	83
{PI3K=OFF}†	Yes	No	98.0	79	50.5	-8
{RAS=OFF}†	Yes	No	99.6	82	53.7	-2
{GRB2=OFF}†	No	No	54.6	0	54.3	-1
{MEK=OFF}†	Yes	No	100.0	83	54.4	0
{ERK=OFF}†	Yes	No	100.0	83	54.3	-1
{IL2RBT=OFF}†	Yes	No	99.9	83	54.4	-1
{IL2RB=OFF}†	Yes	No	99.9	83	54.3	-1
Apoptosis stable motif blocking interventions ($B_{Apoptosis}$)						
{SPHK1=ON}	Yes	Yes	8.8	-84	7.7	-86
{PDGFR=ON}	Yes	Yes	13.9	-75	13.7	-75
{Ceramide=OFF}	Yes	Partial	11.2	-79	33.5	-39
{sFas=ON}	Yes	No	11.3	-79	48.5	-11
{Fas=OFF}	Yes	No	10.1	-81	43.4	-21
{TBET=OFF}†	Yes	Yes	0.0	-100	0.5	-99

Intervention	Successful?	Long-term?	Apoptosis % (permanent intervention)	Relative apoptosis % change (permanent intervention)	Apoptosis % (nonpermanent intervention)	Relative apoptosis % change (nonpermanent intervention)
Single interventions of T-LGL stable motif control sets						
{SPHK1=ON}	Yes	Yes	8.8	-84	7.7	-86
{PDGFR=ON}	Yes	Yes	13.9	-75	13.7	-75
{Ceramide=OFF}	Yes	Partial	11.2	-79	33.5	-39
Single interventions of apoptosis stable motif control sets						
{TBET=ON}	No	No	54.9	0	54.4	0
{Ceramide=ON}	Yes	Yes	99.9	83	99.9	83
{RAS=ON}	No	No	55.0	1	54.6	0
{GRB2=ON}	No	No	54.9	0	54.5	0
{IL2RB=ON}	No	No	54.8	0	54.4	0
{IL2RBT=ON}	No	No	54.8	0	54.4	0
{ERK=ON}	No	No	54.9	0	54.5	0
{MEK=ON}	No	No	54.8	0	54.4	0
{PI3K=ON}	No	No	55.3	1	54.9	0
