# Supplementary material for: Cell Fate Reprogramming by Control of Intracellular Network Dynamics
Source: PLoS Comput Biol. 2015 Apr 7;11(4):e1004193. doi: 10.1371/journal.pcbi.1004193 (PMC4388852; doi:10.1371/journal.pcbi.1004193)
Supplement: S4 Table — The relative attractor % change is defined as (attractor %−normal attractor %)/(normal attractor %), where the normal attractor % is the percentage of initial conditions that go to the attractor of interest when no intervention is applied. The normal attractor percentages are 50.0%, 45.4%, 2.8%, and 1.8% for the Th1, Th2, Th17, and Treg helper T cell subtypes, respectively. Interventions marked with † appear in more than one control strategy or target attractor in Table 2. The percentages are significant in the digits shown and have an estimated absolute error (standard deviation of the mean) of 6⋅10−3[%p Attr(100%−%p Attr)]1/2 %, where %p Attr is the percentage shown (e.g. 0.06% for a %p Attr of 1%, and 0.3% for a %p Attr of 50%). (PDF) [file pcbi.1004193.s017.pdf]

S4 Table. Validation of the stable motif control intervention targets in Table 2 for the helper T cell differential equation network model. The relative attractor % change is defined as (attractor % − normal attractor%)/(normal attractor %), where the normal attractor % is the percentage of initial conditions that go to the attractor of interest when no intervention is applied. The normal attractor percentages are 50.0 %, 45.4 %, 2.8 %, and 1.8 % for the Th1, Th2, Th17, and Treg helper T cell subtypes, respectively. Interventions marked with † appear in more than one control strategy or target attractor in Table 2. The percentages are significant in the digits shown and have an estimated absolute error (standard deviation of the mean) of $6 \cdot 10^{-3}[\%p_{Attr}(100\% - \%p_{Attr})]^{1/2}$ %, where $\%p_{Attr}$ is the percentage shown (e.g. 0.06% for a $\%p_{Attr}$ of 1%, and 0.3% for a $\%p_{Attr}$ of 50%).

Intervention	Successful?	Long-term?	Attractor % (permanent intervention)	Relative attractor % change (permanent intervention)	Attractor % (nonpermanent intervention)	Relative attractor % change (nonpermanent intervention)
Th1 stable motif control interventions (C_{Th1})						
{TBET=ON}	Yes	Yes	100.0	100	100.0	100
Th2 stable motif control interventions (C_{Th2})						
{GATA3=ON}	Yes	Yes	100.0	120	100.0	120
Th17 stable motif control interventions (C_{Th17})						
{GATA3=OFF, FOXP3=OFF, TBET=OFF, STAT3=ON}	Yes	Yes	100.0	3437	100.0	3437
{GATA3=OFF, FOXP3=OFF, TBET=OFF, IL10=ON}	Yes	Yes	100.0	3437	100.0	3437
{GATA3=OFF, FOXP3=OFF, TBET=OFF, IL10R=ON}	Yes	Yes	100.0	3437	100.0	3437
{GATA3=OFF, FOXP3=OFF, TBET=OFF, IL21=ON}	Yes	Yes	100.0	3437	100.0	3437
{GATA3=OFF, FOXP3=OFF, TBET=OFF, IL21R=ON}	Yes	Yes	100.0	3437	100.0	3437
{GATA3=OFF, FOXP3=OFF, TBET=OFF, IL23R=ON, RORGT=ON}	Yes	Yes	100	3437	100	3437
Treg stable motif control interventions (C_{Treg})						
{GATA3=OFF, FOXP3=ON, TBET=OFF}	Yes	Yes	100.0	5613	100.0	5613
{GATA3=OFF, TBET=OFF, STAT3=OFF}	Yes	Yes	100.0	5613	100.0	5613
{GATA3=OFF, TBET=OFF, IL23R=OFF, IL10R=OFF, IL21R=OFF}	Yes	Yes	100.0	5613	100.0	5613
{GATA3=OFF, TBET=OFF, IL23R=OFF, IL10=OFF, IL21R=OFF}	Yes	Yes	100.0	5613	100.0	5613
{GATA3=OFF, TBET=OFF, IL23R=OFF, IL10R=OFF, IL21=OFF}	Yes	Yes	100.0	5613	100.0	5613

Intervention	Successful?	Long-term?	Attractor % (permanent intervention)	Relative attractor % change (permanent intervention)	Attractor % (nonpermanent intervention)	Relative attractor % change (nonpermanent intervention)
Treg stable motif control interventions (C_{Treg}) (continuation)						
{GATA3=OFF, TBET=OFF, IL23R=OFF, IL10=OFF, IL21=OFF}	Yes	Yes	100.0	5613	100.0	5613
Single interventions of Th17 stable motif control sets						
GATA3=OFF}	No	No	1.7	-41	1.7	-40
{FOXP3=OFF}	Partial	Partial	4.3	54	3.7	30
{TBET=OFF}	Partial	Partial	3.8	35	3.8	34
{STAT3=ON}	Partial	Partial	4.0	41	4.0	41
{IL10=ON}	Partial	Partial	3.8	33	3.8	34
{IL10R=ON}	Partial	Partial	3.8	34	3.8	35
{IL21=ON}	Partial	Partial	3.8	33	3.8	34
{IL21R=ON}	Partial	Partial	3.8	34	3.8	35
{IL23R=ON}	Partial	Partial	3.8	34	3.8	35
{RORGT=ON}	No	No	3.0	7	3.0	6
Single interventions of Treg stable motif control sets						
{GATA3=OFF}	No	No	1.4	-18	1.6	-10
{FOXP3=ON}	Yes	Yes	4.8	172	4.7	167
{TBET=OFF}	Partial	Partial	2.6	47	2.6	49
{STAT3=OFF}	Yes	Yes	4.1	137	4.2	137
{IL21=OFF}	Partial	Partial	2.4	35	2.4	39
{IL21R=OFF}	Partial	Partial	2.5	40	2.6	46
{IL23R=OFF}	Yes	Yes	2.0	14	2.0	13
