# Supplementary material for: Cell Fate Reprogramming by Control of Intracellular Network Dynamics
Source: PLoS Comput Biol. 2015 Apr 7;11(4):e1004193. doi: 10.1371/journal.pcbi.1004193 (PMC4388852; doi:10.1371/journal.pcbi.1004193)
Supplement: S6 Table — The percentages are significant in the digits shown and have an estimated absolute error (standard deviation of the mean) of 5⋅10−3[%p Attr(100%−%p Attr)]1/2 %, where %p Attr is the percentage shown (e.g. 0.05% for a %p Attr of 1%, and 0.25% for a %p Attr of 50%). (PDF) [file pcbi.1004193.s019.pdf]

S6 Table. Validation of some stable motif control intervention targets in Table 1 for different Hill coefficients (n) in the T-LGL leukemia differential equation network model with randomly chosen τ_i and θ_i. The percentages are significant in the digits shown and have an estimated absolute error (standard deviation of the mean) of $5 \cdot 10^{-3}[\%p_{Attr}(100\% - \%p_{Attr})]^{1/2}$ %, where $\%p_{Attr}$ is the percentage shown (e.g. 0.05% for a $\%p_{Attr}$ of 1%, and 0.25% for a $\%p_{Attr}$ of 50%).

Intervention	Apoptosis % (permanent intervention)					Apoptosis % (nonpermanent intervention)				
	n									
	1	1.5	2	2.5	3	1	1.5	2	2.5	3
T-LGL stable motif control interventions (C_{TLGL})										
{S1P=ON}	0.0	0.0	0.0	0.0	0.0	0.0	0.0	0.0	0.0	0.0
{Ceramide=OFF, PDGFR=ON}	0.0	0.0	0.0	0.0	0.0	0.0	0.0	0.0	0.0	0.0
Apoptosis stable motif control interventions ($C_{Apoptosis}$)										
{SPHK1=OFF}	100.0	100.0	100.0	99.9	99.5	100.0	100.0	100.0	99.9	99.5
{TBET=ON, Ceramide=ON, IL2RB=ON}	100.0	100.0	100.0	100.0	100.0	100.0	100.0	100.0	100.0	100.0
